# What do we know about the effects of exposure to ‘Low alcohol’ and equivalent product labelling on the amounts of alcohol, food and tobacco people select and consume? A systematic review

**DOI:** 10.1186/s12889-016-3956-2

**Published:** 2017-01-12

**Authors:** Ian Shemilt, Vivien Hendry, Theresa M. Marteau

**Affiliations:** Behaviour and Health Research Unit, Institute of Public Health, University of Cambridge, Cambridge, CB2 0SR UK

**Keywords:** Alcohol, Labelling, Public health, Alcohol policy, Systematic review, Meta-analysis

## Abstract

**Background:**

Explicit labelling of lower strength alcohol products could reduce alcohol consumption by attracting more people to buy and drink such products instead of higher strength ones. Alternatively, it may lead to more consumption due to a ‘self-licensing’ mechanism. Equivalent labelling of food or tobacco (for example “Low fat” or “Low tar”) could influence consumption of those products by similar mechanisms. This systematic review examined the effects of ‘Low alcohol’ and equivalent labelling of alcohol, food and tobacco products on selection, consumption, and perceptions of products among adults.

**Methods:**

A systematic review was conducted based on Cochrane methods. Electronic and snowball searches identified 26 eligible studies. Evidence from 12 randomised controlled trials (all on food) was assessed for risk of bias, synthesised using random effects meta-analysis, and interpreted in conjunction with evidence from 14 non-randomised studies (one on alcohol, seven on food and six on tobacco). Outcomes assessed were: quantities of the product (i) selected or (ii) consumed (primary outcomes - behaviours), (iii) intentions to select or consume the product, (iv) beliefs associated with it consumption, (v) product appeal, and (vi) understanding of the label (secondary outcomes – cognitions).

**Results:**

Evidence for impacts on the primary outcomes (i.e. amounts selected or consumed) was overall of very low quality, showing mixed effects, likely to vary by specific label descriptors, products and population characteristics. Overall very low quality evidence suggested that exposure to ‘Low alcohol’ and equivalent labelling on alcohol, food and tobacco products can shift consumer perceptions of products, with the potential to ‘self-licence’ excess consumption.

**Conclusions:**

Considerable uncertainty remains about the effects of labels denoting low alcohol, and equivalent labels, on alcohol, food and tobacco selection and consumption. Independent, high-quality studies are urgently needed to inform policies on labelling regulations.

**Electronic supplementary material:**

The online version of this article (doi:10.1186/s12889-016-3956-2) contains supplementary material, which is available to authorized users.

## Background

‘Low alcohol’ and equivalent labels are those that incorporate descriptors (terms) such as ‘Low’ ‘Lighter’ , ‘Dealcoholised’ , or ‘Free’ to denote low, reduced or zero alcohol strength in beverages. Current UK legislation limiting use of the specific label descriptors ‘low alcohol’ , ‘dealcoholised’ and ‘alcohol free’ to beverages of ≤1.2%, ≤0.5% (if subjected to a dealcoholizing process) and ≤0.05% alcohol by volume (ABV) respectively, is subject to a sunset clause until the end of 2018, at which point it will be revoked [1]. This provides an opportunity for policy makers to consider whether amending the definitions and number of regulated terms – and thereby extending the range of products to which ‘low alcohol’ and equivalent labels can be applied – could contribute to better consumer understanding and policies aiming to reduce alcohol consumption and related harm at a population level.

The use of different descriptors or amended definitions for ‘Low alcohol’ labelling on beverages could reduce the number of alcohol units consumed by enabling drinkers to select lower strength alcohol products instead of regular strength products (substitution, with no change in the volume of drinks consumed) [2]. Alternatively, a wider range of descriptors for low alcohol products could increase the amount of alcohol units consumed by increasing the number of opportunities perceived suitable for consuming alcohol, or engendering a ‘self-licensing effect’ [3] (i.e. acting indulgently following a virtuous choice), such that people overconsume lower strength products, resulting in more units of alcohol consumed overall. A similar ‘self-licensing effect’ has been observed in relation to food product labelling, with products labeled with ‘low fat’ and equivalent descriptors consumed in unhealthily large quantities [4], and has also been a key motivating factor behind the introduction of legislation banning use of ‘mild’ , ‘light’ and other similar label descriptors on tobacco packaging in several countries [5]. Moreover, when food product label descriptors denote the amount of a single nutrient, such as ‘low fat’ , this can lead to a ‘health halo’ , whereby people generalize that the labelled food is healthier in all nutrition aspects, whether or not this is actually the case [6–8].

This systematic review examined evidence for the effects of ‘Low alcohol’ and equivalent labelling of alcohol, food and tobacco products on product selection and consumption among adults. Its aims were to synthesise the available research evidence to inform current policy debate concerning potential changes to alcohol labelling regulations, and to highlight gaps and key uncertainties in the current evidence base. The review was extended to encompass studies of equivalent labels on food and tobacco products when preliminary scoping searches, conducted in conjunction with developing the protocol for this review [9], indicated a likely paucity of evidence from studies of labels denoting lower strength alcohol products. The decision to investigate the effects of this kind of labelling in three product categories was based on an assumption that, while effect modifiers may differ between alcohol, food and tobacco products (e.g. alcohol-induced disinhibition may influence selection and consumption decisions in the case of alcohol), the proposed theory of change about how exposure to labelling of this kind might influence health behaviour is broadly similar. First, exposed people become aware of a label descriptor on a product. Second, their perception of the product is altered by this awareness; that is, the label descriptor acts as a prompt or cue, activating semantic associations that influence people – correctly or incorrectly – to perceive the product to be healthier, or less harmful to health, relative to the same product labelled with a descriptor denoting higher amounts of the referent substance, or with no equivalent labelling. Third, this altered perception motivates people to initiate a behaviour; that is, to select and consume either the low alcohol, fat, or calorie product, or the stronger, or higher fat or calorie, version, and thereby consume more or less of the referent health harming substance. Finally, through repeated exposure to these kinds of label descriptors and repeated activation of the same semantic associations, the associations are learnt (internalised) and the initiated behaviour becomes habitual.

## Methods

The systematic review protocol was prospectively registered on the PROSPERO database: CRD42014013008 [9]. A Preferred Reporting Items for Systematic Reviews and Meta-Analyses statement [10] is provided in Additional file 1.

### Eligibility criteria

Broad inclusion criteria were applied due to the relative paucity of available literature in this area. Comparative studies of any design (including randomised and non-randomised studies) and length of follow-up were included if they assessed exposure to labels that incorporated descriptors such as ‘low’ , ‘light’ , ‘mild’ (absolute), ‘lower’ , ‘lighter’ , ‘reduced’ , ‘x% less’ , ‘half’ (relative), ‘no’ , ‘no added’ , ‘zero’ or ‘free’ (absent) to denote either a low strength of – or the presence of a small or zero amount of a substance in – alcoholic beverage, food (including non-alcoholic beverages) or tobacco products, among participants aged ≥16 years. Studies investigating food allergen labels (e.g. ‘gluten free’) or similar warning labels were excluded. Eligible comparators were: (a) exposure to labels denoting either a higher strength version of the same product or the presence of a larger amount of the same substance in the same product; or (b) exposure to the same product with no equivalent labelling. Eligible studies also had to assess the effects of (or associations between) exposure in terms of one of the following outcome constructs: (i) quantities of the product selected (ii) quantities of the product consumed (primary outcomes – behaviours), (iii) intentions to select, purchase or consume the product, (iv) beliefs associated with its consumption, (v) product appeal, or (vi) understanding of the label (secondary outcomes – cognitions). As such, outcomes assessed in this systematic review encompassed intermediate endpoints along the length of the causal chain proposed in the theory of change outlined above. There were no eligibility restrictions for study publication status, date or language.

### Search methods and study selection procedures

Eligible studies were located using electronic searches of PubMed, Google Scholar™ and Google™ combined with extensive snowball searches [11] comprising both forward and backward citation tracking. Search terms were derived from PubMed MeSH headings and keywords including: ‘food labeling’ , ‘product labeling’ , ‘alcohol deterrents’ , ‘alcohol consumption’ , ‘drinking’ , ‘drinking behaviour’ , ‘food and beverages’ , ‘diet’ , ‘eating’ , ‘food supply’ , ‘food habit’ , ‘smoking’ , or ‘tobacco use’. Searches were conducted between 6 October and 19 December 2014. Searches of the Database of Abstracts of Reviews of Effects (DARE) and PubMed were concurrently conducted to identify relevant systematic reviews or traditional narrative literature reviews citing reports of potentially eligible primary studies. Provisional eligibility decisions based on title-abstract screening were made by one reviewer (VH). Final eligibility decisions, based on examination of full-text study reports, were made by one reviewer (VH) and checked by a second (IS). Multiple full-text reports of the same study were identified, linked and treated as a single study. Full-text reports comprising multiple eligible studies were identified and each study was processed separately.

### Data collection, risk of bias assessment and analysis

A data extraction form was developed from an established template [12] and piloted on five included studies. Data on the characteristics and results of included studies were extracted by one reviewer (VH) and checked by a second (IS), consulting with a third (TMM) to classify each outcome measured in each study into one of six pre-specified primary or secondary outcome constructs (i-vi). For included studies comprising multiple eligible comparisons, data were extracted for each comparison. Included studies were classified as randomised controlled trials (RCTs) (experimental studies) or non-randomised studies (NRSs) (quasi-experimental or non-experimental studies) and then further classified based on study design features [13]. These study design classifications were based on features of the specific within-study comparison(s) of interest in this review, which in some cases led to classifications that differed from ‘study design labels’ (e.g. cluster-randomised controlled trial) ascribed by study authors (who may have designed their studies to address different (or variant) objectives to those of this review). If an included RCT reported data needed to compute effect sizes for one or more outcome measures in a figure but not in main text or tables, data were extracted by manually taking measurements from enlarged, printed images of original figures. One reviewer (IS) assessed included RCTs using the Cochrane Risk of Bias Tool [14] and recorded issues concerning the validity of included NRSs. Study-level effect sizes were computed for each eligible primary or secondary outcome measure as the standardised mean difference (SMD) between comparison groups.

Sample sizes used to compute study-level effect sizes (both intervention and comparator) were halved for eligible comparisons derived from the same RCT that comprised non-independent samples. If an RCT reported multiple measures of an outcome construct, the study-level effect size was computed with respect to the measure judged to most comprehensively capture that construct. For example, if an RCT measured the appeal of a food product using participant ratings of ‘visual appeal’ , ‘flavour’ , and ‘overall liking’ , the effect size was computed using data for ‘overall liking’. Study-level effect sizes from RCTs were next combined for each outcome using a series of generic inverse-variance random-effects meta-analyses [15], conducted using Review Manager 5.3. In practice, sufficient useable data were available from included RCTs to allow us to conduct three of the six planned, separate meta-analyses: namely, on consumption, product appeal, and understanding of the label (see ‘Results’).

Statistical heterogeneity was assessed by inspection of graphical displays of each SMD and its 95% confidence interval, and a formal statistical test of homogeneity (I^2^) [16]. Funnel plots were drawn to inform assessment of reporting biases [17] but planned statistical tests to formally investigate asymmetry were not conducted due to small sample sizes. Planned sub-group analyses were next conducted, subject to available data, to explore potential differential effects by characteristics of study design, label descriptors, or participants. Overall quality of evidence for each summary effect size was assessed using the GRADE approach [18]. Finally, all study-level data were tabulated and summarised using an overarching narrative synthesis.

## Results

### Results of the search

**Fig. 1:**
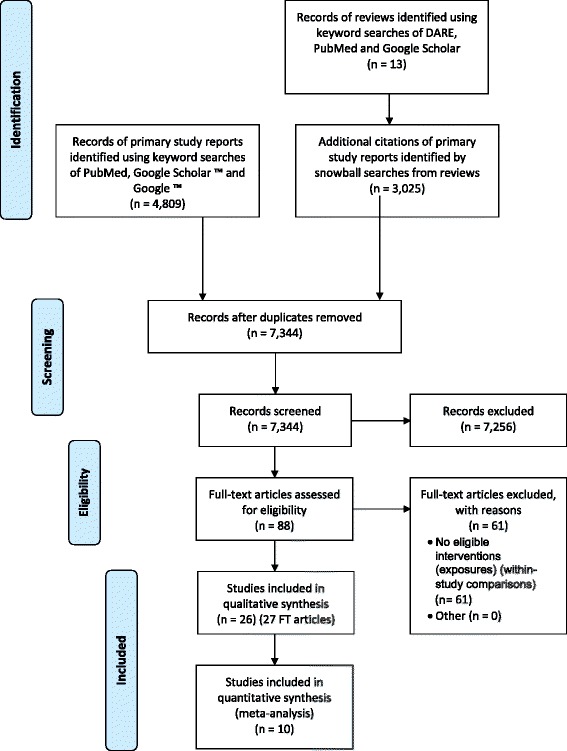
PRISMA study flow diagram

Figure 1 shows the flow of studies through the systematic review process. Bibliographic details of 13 relevant reviews identified by searches of DARE and PubMed are provided in Additional file 2. All sources yielded a total of 7834 primary study records, of which 490 were duplicates and the remaining 7344 were screened. Eighty-eight study records were selected as provisionally eligible, of which 61 were excluded based on full-text study screening. Twenty-six studies, reported in 27 full-text articles published in English between 1984 and 2014, were accepted into the review [19–45] (see also Additional file 2).

### Description of included randomised controlled trials (RCTs)

Twelve of 26 included studies were classified as RCTs [19, 24–27, 31, 32, 34, 36, 38, 40–43]. All 12 RCTs investigated food labelling. Their key characteristics and results are summarised in Additional file 3: Table S1, with additional details provided in Additional file 4. Eleven RCTs were individually randomised – of which seven had a parallel group (between-subjects) design and four had a crossover (within-subjects) design – and the other was a cluster RCT with a crossover design. Five RCTs were conducted in the United Kingdom, five in the United States of America, and two in Finland.

### Description of included non-randomised studies

Fourteen of 26 included studies were classified as non-randomised studies (NRSs) (quasi-experimental or non-experimental studies) [20–23, 25, 28–31, 33, 35, 37, 39, 44, 45]. Key characteristics and results of these 14 NRSs are summarised in Additional file 5: Table S2, with further details in Additional file 4. Only one NRS investigated alcohol labelling [22], seven investigated food labelling [21, 25, 30, 31, 33, 35, 37, 44] and six investigated tobacco labelling [19, 23, 28, 29, 39, 45]. These NRSs investigated exposure to labelling among adults living in the USA (five studies), Finland (two studies), Canada (two studies), the UK (two studies), The Netherlands (one study), Thailand (one study), and Australia, Canada and the UK (one study).

### Risk of bias in included studies

**Fig. 2:**
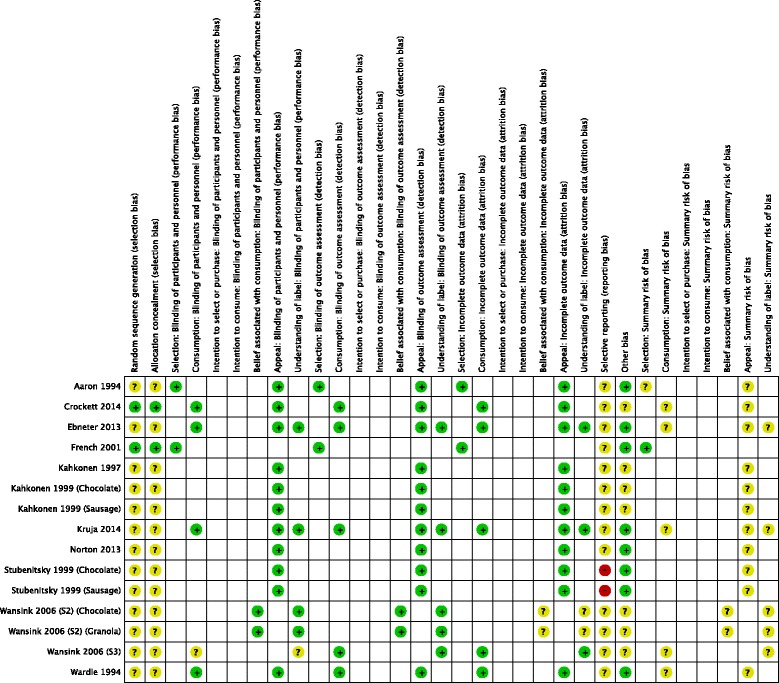
Risk of bias summary: review authors’ judgements about each risk of bias item for each included RCT

Figure 2 summarises risk of bias assessments for the 12 included RCTs of food labelling (study-level risk of bias tables are presented in Additional file 6). Summary risk of bias judgements reflected risk of bias for each primary or secondary outcome within each study. Summary judgements were determined by judgements in three domains pre-specified as most important in the current review: selection bias, attrition bias and baseline comparability of participants between groups (classified as ‘Other bias’ in Fig. 2). The principal result of this assessment was that 11 of 12 included RCTs were judged to be at overall unclear risk of bias, due primarily to incomplete or unclear reporting of study methods and procedures. One RCT was judged to be at overall low risk of bias (selection outcome) [27].

Some general issues concerning the internal validity of included NRSs warrant consideration. Compared with RCTs, NRSs rely on more stringent, sometimes non-verifiable, assumptions to be met in order to confer confidence that the risk of confounding is sufficiently low to allow inferences to be made about causal effects. Two of the seven included NRSs of food product labels were classified as quasi-experimental (QE) studies. The first QE study [21] was designed and implemented as an RCT, but since the within-study comparison eligible for consideration in this review did not reflect the random assignment, this study was reclassified as a controlled before-and-after (CBA) comparison for the current analysis. In CBAs, observations are made before and after an intervention, both in a group that receives the intervention and in a control group that does not. The second QE study [35] was a difference-in-differences (DID) study, with supermarkets as the units of observation. DID studies compare the change in outcomes over time between a group that receives the intervention and a control group. CBAs and DIDs aim to exploit variation in the exposure of interest both within and between observational units to control for all unobserved time-constant confounders. They are examples of QE studies that rely on covariate adjustment to control for confounding, which distinguishes them from QEs that utilise design-based approaches to control for confounding by exploiting exogenous variation in the exposure of interest [46]. Design-based approaches are considered *a priori* more credible or ‘stronger’ for causal inference than approaches that rely on covariate adjustment [47, 48]. The other five NRSs of food labelling, and all seven NRSs of tobacco labelling, were classified as various types of non-randomised studies (see Additional file 5: Table S2), from which valid causal inferences could not confidently be drawn.

### Effects of exposure to ‘low alcohol’ and equivalent labels

#### Alcohol labelling

Data collected from 230 adult participants in a single, USA-based non-randomised controlled trial with a crossover design [22], suggested participants correctly perceived the calorie and carbohydrate content of the alcohol product to be lower when exposed to beer bottles labelled as ‘Light’ than when exposed to beer bottles labelled or denoted as ‘Regular’. It was not possible to derive a clear result for any effect of exposure to beer bottles labelled as ‘Light’ (versus ‘Regular’) on participants’ intention to consume (measured by asking ‘Given the information shown on the front and the back of the bottle, would the available information increase or decrease the amount you would drink, that is, your consumption level?’). This was because outcome data were not reported for this specific comparison, which was not the main focus of the study. No other eligible studies of alcohol labelling were identified (see ‘Discussion’).

#### Food labelling

##### Product selection

Two RCTs assessed the effect of exposure to ‘low fat’ or equivalent labels on quantities of food selected. One was an individual RCT with a crossover design [19], which exposed 101 consumers to a piece of bread and a glass bowl of margarine of 39.6% fat, and a label containing *either* the descriptor ‘Reduced-fat margarine (40% fat)’ , *or* the descriptor ‘Full-fat margarine (80% fat)’ attached. Participants selected more reduced-fat spread when exposed to the ‘Reduced-fat’ label (SMD: 0.31, 95% CI: 0.04 to 0.58) (Table 1). The second was a cluster randomised controlled trial with a crossover design [27], conducted in 55 vending machines located in 12 schools or worksites, which included a comparison of exposures to vending machines that included low-fat snack products and *either* had labels placed on the vending machines labelling the products as low-fat (precise wording was not reported), *or* had no equivalent labelling. This study reported finding no difference between these conditions in the proportion of low-fat snack items sold or average sales of low-fat snack items per site (reported data did not enable estimation of a study-level effect size in this case).

**Table 1:** Results of statistical analyses of outcome data from RCTs that investigated food product labels

Outcome or subgroup	Independent comparisons	Total participants	Statistical method	Effect estimate: SMD (95% CI)	Test for overall effect or subgroup differences
Product selection (with or without purchase)	1	202	SMD (IV, Random, 95% CI)	0.31 (0.04 to 0.58)	N/A
Product consumption	5	680	SMD (IV, Random, 95% CI)	0.27 (−0.12 to 0.66)^a^	*Z* = 1.36, *P* = 0.17
- Intervention label denotes absence (“Fat-free)	1	256	SMD (IV, Random, 95% CI)	0.42 (−0.08 to 0.91)	
- Intervention label denotes absolute amount (“Low-fat”)	4	424	SMD (IV, Random, 95% CI)	0.38 (−0.21 to 0.98)^a^	Chi^2^ = 5.15, df = 1, *P* = 0.02
- Female participants only	2	343	SMD (IV, Random, 95% CI)	0.45 (−0.02 to 0.92)^a^	
- Male and female participants	3	337	SMD (IV, Random, 95% CI)	0.15 (−0.35 to 0.65)^a^	Chi^2^ = 0.75, df = 1, *P* = 0.39
- Parallel group (between-subjects) design	3	344	SMD (IV, Random, 95% CI)	0.05 (−0.16 to 0.26)^a^	
- Crossover (within-subjects) design	2	336	SMD (IV, Random, 95% CI)	0.51 (0.29 to 0.72)^a^	Chi^2^ = 8.76, df = 1, *P* = 0.003
Intention to select or purchase product	0	0	N/A	Not estimable	N/A
Intention to consume product	0	0	N/A	Not estimable	N/A
Belief associated with product consumption	1	34	SMD (IV, Random, 95% CI)	−0.66 (−1.32 to 0.00)	N/A
	1	34	SMD (IV, Random, 95% CI)	−0.14 (−0.79 to 0.51)	N/A
Product appeal	8	1013	SMD (IV, Random, 95% CI)	−0.01 (−0.14 to 0.13)^a^	*Z* = 0.09, *P* = 0.93.
- Intervention label denotes absence (“Fat-free)	2	412	SMD (IV, Random, 95% CI)	−0.01 (−0.21 to 0.18)^a^	
- Intervention label denotes absolute amount (“Low-fat”)	2	167	SMD (IV, Random, 95% CI)	−0.09 (−0.28 to 0.11)^a^	
- Intervention label denotes relative amount (“Reduced-fat)	4	434	SMD (IV, Random, 95% CI)	0.03 (−0.19 to 0.26)^a^	Chi^2^ = 0.23, df = 2, *P* = 0.89
- Female participants only	2	343	SMD (IV, Random, 95% CI)	0.09 (−0.12 to 0.30)^a^	
- Male and female participants	6	670	SMD (IV, Random, 95% CI)	−0.06 (−0.24 to 0.12)^a^	Chi^2^ = 1.10, df = 1, *P* = 0.30
- Parallel group (between-subjects) design	4	301	SMD (IV, Random, 95% CI)	0.00 (−0.22 to 0.23)^a^	
- Crossover (within-subjects) design	4	712	SMD (IV, Random, 95% CI)	−0.03 (−0.23 to 0.17)^a^	Chi^2^ = 0.04, df = 1, *P* = 0.84
Understanding of label	3	155	SMD (IV, Random, 95% CI)	−0.57 (−0.89 to −0.26)^a^	*Z* = 3.57, *P* = 0.0004

*SMD* standardised mean difference, *IV* generic inverse variance, *random* Random effects model, *95% CI* 95% confidence interval, ^a^Summary effect size (pooled estimate), *N/A* not applicable

Four comparisons identified from 3 included NRSs of food product labels assessed quantities selected by participants. A CBA comparison [21] exposed students or staff of a large, urban university to 12 non-alcoholic beverage product lines (including water, diet beverages, and sugar-sweetened beverages) in vending machines located in the main campus building that *either* had bright-coloured ‘0 Calorie, 0 Carbs’ labels placed on the selection panels of water and non-energy containing products, *or* had no equivalent labelling, and found no differences between conditions in terms of average numbers of bottles of water, diet beverages, or sugar-sweetened beverages purchased each week. A DID study [35] exposed supermarket customers to a microwave popcorn product line on a shelf with *either* a label containing the descriptor ‘Low calorie’, *or* a label containing the descriptor ‘Low fat’ placed adjacent to the price tag on the shelf, *or* no equivalent labelling. Average sales over a 4 week period were substantially higher during the ‘Low calorie’ label intervention period, but lower during the ‘Low fat’ label intervention period, compared with no equivalent labelling. A study with one eligible comparison classified as a prospective cohort study [25], which exposed customers to *either* a cafeteria line with labels displaying the descriptor ‘lower calorie’ placed adjacent to an incrementally increasing number of food items (three vegetable dishes; three vegetable dishes and three salads; three vegetable dishes, three salads and three entrées) over 9 weeks, or to a cafeteria line offering the same products with no equivalent labelling, found that: labelling three, six or nine food items was associated with an increase in the probability of participants purchasing lower calorie vegetable items; labelling six and nine food items (but not three – vegetable dishes only) was associated with an increase in the probability of participants purchasing lower calorie salad items; labelling three, six or nine food items was not associated with a change in the probability of participants purchasing lower calorie entrées.

##### Product consumption

**Fig. 3:**
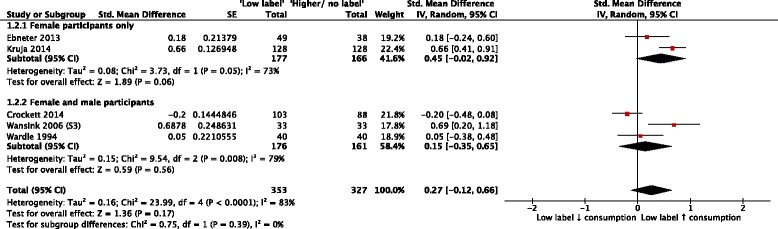
Forest plot of comparison: food products - exposure to ‘low fat’ or equivalent labels versus exposure to ‘higher fat’ or equivalent labels or no equivalent labels; outcome – product consumption

**Fig. 4:**
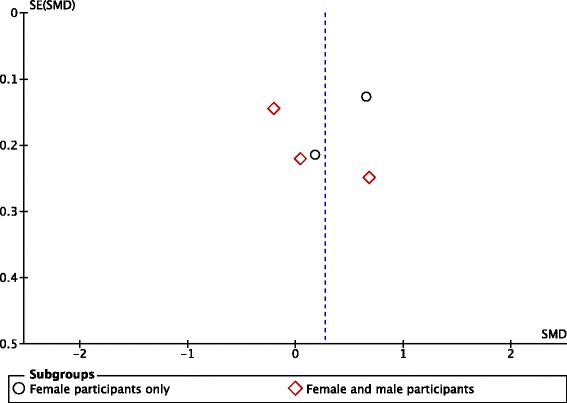
Funnel plot of comparison: food products - exposure to ‘low fat’ or equivalent labels versus exposure to ‘higher fat’ or equivalent labels or no equivalent labels; outcome – product consumption

Five comparisons from five included RCTs assessed the effect of exposure to ‘low fat’ or equivalent labels on quantities of food or food energy consumed [24, 26, 36, 41–43]. Random effects meta-analysis of these outcome data, collected from a total of 680 participants, showed a summary effect size (SMD) of 0.27 (95% CI: −0.12 to 0.66, *p* = 0.17, *I* ^2^ = 83%), consistent with evidence for no effect of ‘low fat’ or equivalent labels on consumption (Fig. 3). Visual inspection of the corresponding funnel plot (Fig. 4) did not identify a pattern consistent with publication bias. The overall quality of this body of evidence from RCTs was rated as very low, due to concerns about study limitations (unclear risk of bias), inconsistency (substantial unexplained heterogeneity) and indirectness (the majority of outcome data were from student and/or staff of higher education institutions, so may not be generalisable to a general adult population).

Planned subgroup analyses explored differential effects by study design, label descriptor (‘absence’ versus ‘absolute’), and participants’ gender (see Table 1). Tests for subgroup differences indicated differential effects by label descriptor (Chi^2^ = 5.15, df = 1, *P* = 0.02) and by study design (Chi2 = 8.76, df = 1, *P* = 0.003) but not by gender (Chi2 = 0.75, df = 1, *P* = 0.39) (Table 1). Regarding label descriptors, no difference in consumption was observed in either subgroup (Intervention label denotes absence (‘Fat-free’) SMD: 0.42, 95% CI: −0.08 to 0.91; Intervention label denotes absolute amount (‘Low-fat’) SMD: 0.38, 95% CI: −0.21 to 0.98). Regarding study designs, no difference was observed within the ‘parallel group design’ subgroup (SMD: 0.05, 95% CI: −0.16 to 0.26). However, a difference in effect was observed within the ‘crossover design’ subgroup (SMD: 0.51, 95% CI: 0.29 to 0.72), suggesting participants in these studies consumed more food when exposed to ‘low fat’ or equivalent labels than when exposed to ‘higher fat’ labels or no equivalent labelling. The results of subgroup analyses should be viewed with caution due to the small numbers of studies and participants within each subgroup, and the observational nature of the analyses. No included NRSs of food product labels assessed participants’ consumption.

##### Intentions to select or purchase and consume product

No included RCTs of food product labels measured participants’ intentions to select, purchase or consume the product. One UK-based non-randomised controlled trial with a crossover design [44] exposed 36 normal weight adults to foods labelled with *either* a ‘Lower fat’ , *or* a ‘Normal fat’ , *or* a ‘Higher fat’ descriptor in a laboratory setting, and found participants’ ratings of the likelihood of purchasing the foods were higher when exposed to foods labelled as ‘Lower fat’ than when exposed *either* to foods labelled as ‘Normal fat’ *or* foods labelled as ‘Higher fat’. Also, one cross-sectional study [37] exposed 46 adult participants to a box containing one pouch of chicken ‘Cup a Soup’ and *either* labelled ‘Now with reduced salt’ , *or* with no equivalent labelling, and found no differences between conditions on two measures of intention to consume the product.

##### Belief associated with product consumption

Four comparisons from two included RCTs assessed the effect of exposure to ‘low fat’ or equivalent labels on participants’ beliefs associated with consumption of the product [40–42]. Useable outcome data were reported in one of these [42], which had a parallel group design and exposed participants to two transparent measuring cups (20-ounce capacity), one containing 10 ounces of M&M’s (1380 calories) and one containing 10 ounces of regular granola (1330 calories), either labelled as ‘Low-Fat M&M’s’ and ‘Low-Fat Granola’ , *or* labelled as ‘Regular M&M’s’ and ‘Regular Granola’. Study-level effect sizes for participants’ self-ratings of ‘anticipated guilt’ associated with consumption of M&M’s and granola were SMD: −0.14 (95% CI: −0.79 to 0.51) and SMD: −0.66 (95%CI: −1.32 to 0.00), consistent with no differences between groups. A second RCT with a parallel group design [40], exposed 71 adult consumers to packs of reduced fat pork sausages and reduced fat milk chocolate bars that were labelled with *either* the generic product name and the descriptor ‘Reduced-fat’ *or* the generic product name only, and measured ‘expected fillingness’ but reported no useable outcome data, results or conclusions specific to these randomised comparisons (which were not the focus of the study).

Three comparisons from two non-randomised laboratory studies of food labelling, described above, assessed beliefs associated with product consumption [37, 43]. The first found no difference between ratings of expected healthiness of the product when participants were exposed to boxes of soup labelled with the descriptor ‘Now with reduced salt’ and when they were exposed to boxes of soup with no equivalent labelling [37]. The second found participants’ self-rated belief that the foods were healthy was higher when exposed to foods labelled as ‘Lower fat’ than when exposed either to foods labelled as ‘Normal fat’ or foods labelled as ‘Higher fat’; whilst belief that foods were ‘fatty’ was lower when exposed to foods labelled as ‘Lower fat’ than when exposed to foods labelled as ‘Normal fat’ and to foods labelled as ‘Higher fat’ [43].

##### Product appeal

**Fig. 5:**
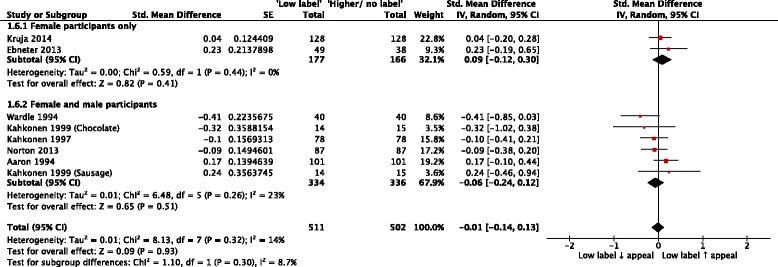
Forest plot of comparison: Food products - Exposure to ‘Low fat’ or equivalent labels versus exposure to ‘Higher fat’ or equivalent labels or no equivalent labels; Outcome – Product appeal

**Fig. 6:**
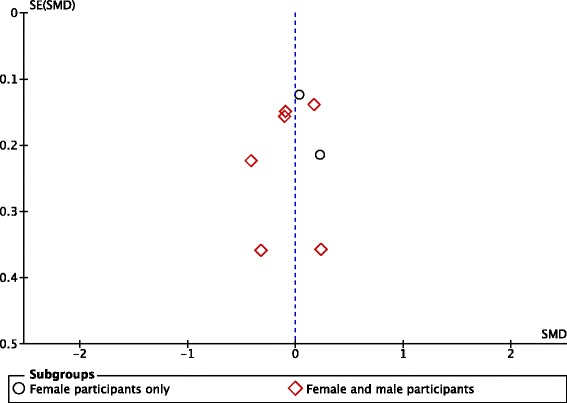
Funnel plot of comparison: food products - exposure to ‘low fat’ or equivalent labels versus exposure to ‘higher fat’ or equivalent labels or no equivalent labels; outcome – product appeal

Ten comparisons identified from eight included RCTs assessed the effect of exposure to eligible labels on product appeal. Useable outcome data were available for meta-analysis from eight independent comparisons, from seven RCTs [19, 26, 31, 32, 34, 36, 38, 43]. Random effects meta-analysis of these outcome data (Fig. 5), collected from a total of 1013 participants, showed a summary effect size (SMD) of −0.01 (95% CI: −0.14 to 0.13, *p* = 0.93, *I* ^2^ = 14%), consistent with evidence for no effect of ‘Low fat’ or equivalent labels on appeal. Visual inspection of the corresponding funnel plot (Fig. 6) did not identify evidence consistent with publication bias. Planned subgroup analyses to explore differential effects by study design, label descriptor (‘absence’ versus ‘absolute’), and participants’ gender did not indicate any differences (see Table 1). The overall quality of this evidence was rated as very low due to concerns about study limitations (unclear risk of bias), indirectness (a substantive proportion of data were from student and/or staff bodies of higher education institutions) and imprecision (the total number of participants included in the meta-analysis did not exceed the optimal information size with respect to the point estimate summary effect size [49]). The other RCT [35], described above, reported finding “a small negative effect of exposure to [‘Reduced fat’ labels on participants’] hedonic ratings [of ‘pleasantness’ , ‘overall idealness’ and ‘boredom’] over time, but only for milk chocolate snack bars [and not for pork sausages]”.

Five comparisons from four included NRSs of food labelling assessed ratings of product appeal [30, 31, 33, 37, 44]. Tabulated results indicated that participants exposed to foods labelled as ‘Low-fat, low salt’ , ‘Light’ , ‘Now with reduced salt’ , or ‘Lower fat’ typically (but not universally) rated products as less appealing than those exposed to foods labelled with descriptors denoting the presence of larger amounts of the same substances in the same product (see Additional file 5: Table S2).

##### Understanding of the label

**Fig. 7:**
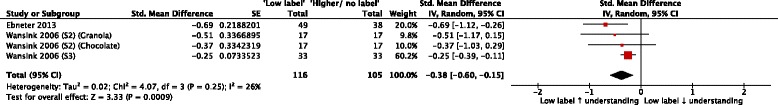
Forest plot of comparison: Food products - Exposure to ‘Low fat’ or equivalent labels versus exposure to ‘Higher fat’ or equivalent labels or no equivalent labels; Outcome – Understanding of the label

Five comparisons from four included RCTs assessed the effect of exposure to eligible labels on participants’ understanding of the label [26, 36, 41, 42]. Useable outcome data were available for meta-analysis from four independent comparisons from three of these RCTs [26, 41, 42]. Random effects meta-analysis of outcome data from these comparisons (Fig. 7), involving 211 participants, showed a summary effect size (SMD) of −0.38 (95% CI: −0.60 to −0.15, *p* = 0.001, *I* ^2^ = 27%), consistent with evidence that exposure to ‘low fat’ or equivalent labels increased understanding of the content of the product (low fat food products are invariably lower in calories than their higher fat equivalents, assuming the same portion size, given fats are the most energy-dense macronutrients). No planned subgroup analyses were feasible due to limited data. The overall quality of this evidence was rated as very low, due to concerns about study limitations (unclear risk of bias), indirectness (a substantive proportion of outcome data were from student and/or staff bodies of higher education institutions) and imprecision (the total number of participants included in the meta-analysis did not exceed the optimal information size).

Results of the other RCT [36], which did not provide useable data for this meta-analysis, indirectly suggested that ‘Low fat’ and equivalent labels might have the potential to licence increased food consumption. This study, in which participants were exposed to a red Solo cup® containing 275 g Nesquick® calcium fortified low-fat chocolate milk labelled with *either* a ‘Fat Free’ descriptor or a ‘Full fat’ descriptor in a laboratory setting, found that participants (on average) overestimated the volume of one serving of this product by a larger amount when it was labelled ‘Fat Free’ than when it was labelled ‘Full fat’. No included NRSs of food product labels assessed participants’ understanding of the label.

#### Tobacco labelling

##### Product selection

No included studies of tobacco product labels assessed product selection (with or without purchase).

##### Product consumption

Two of six NRSs of tobacco labelling (both case series studies) investigated cigarette consumption among adult smokers before and after implementation of national bans on the use of descriptors such as ‘Light’ and ‘Mild’ on cigarette packs. The first [23] found no difference in levels of cigarette consumption or quitting before and after implementation of a nationwide ban on the use of such descriptors. The second [39] found that the proportion of smokers consuming ‘Light’ cigarettes was higher before implementation of a nationwide ban on the use of such descriptors than after. However, there was also a comparable increase in proportion of smokers consuming ‘regular’ cigarettes after the ban had been implemented.

##### Intentions to select, purchase or consume product

No included NRSs of tobacco product labels assessed intention to consume the product and only one assessed intention to select or purchase. This cross-sectional study [20] included a comparison in which 397 adult smokers were exposed to a pair of *Mayfair* branded cigarette packs, one incorporating the label descriptor ‘Light’ , and the other incorporating the label descriptor ‘Full flavour’. Sixty-two per cent of participants selected the pack incorporating the label descriptor ‘Light’ when asked ‘Which one would you buy?’ while the other 38% selected the ‘Full flavour’ option.

##### Beliefs associated with product consumption

Seven comparisons from five included NRSs of tobacco product labels assessed beliefs associated with consumption. Tabulated results from the three cross-sectional studies [20, 28, 29] showed a consistent pattern that the large majority of smokers selected packs incorporating the label descriptor ‘Light’ , ‘Mild’ or ‘Ultra light’ , rather than those incorporating label descriptors ‘Full flavour’ or ‘Regular’ , or no equivalent labelling, when asked which pack they would buy if they were trying to reduce the risk to their health, and which would make it easier to quit smoking (see Additional file 5: Table S2). The other two were case series studies [39, 45] that measured beliefs about light cigarettes among adult smokers’ before and after implementation of national bans on the use of descriptors such as ‘Light’ and ‘Mild’ on cigarette packs. Both studies found that agreement with incorrect beliefs (e.g. ‘Light cigarettes are less harmful’) was higher before implementation of a nationwide ban than after. However, one study also found that initial lower post-ban levels of agreement subsequently recovered to levels observed before the ban [45].

##### Product appeal

Five comparisons from three NRSs – all cross-sectional studies – of tobacco product labels assessed product appeal [20, 28, 29]. There was a consistent pattern of results that the large majority of smokers selected packs incorporating the label descriptor ‘Light’ , ‘Mild’ or ‘Ultra light’ , rather than those incorporating label descriptors ‘Full flavour’ or ‘Regular’ , or no equivalent labelling, when asked which pack of cigarettes they would expect to have the smoothest taste if smoked (or similar question variants) (see Additional file 5: Table S2).

##### Understanding of the label

The same five within-study comparisons from the same three cross-sectional studies also included assessments of participants’ understanding of the label and consistently found that the large majority of smokers selected packs incorporating the label descriptor ‘Light’ , ‘Mild’ or ‘Ultra light’ , rather than those incorporating label descriptors ‘Full flavour’ or ‘Regular’ , or no equivalent labelling, when asked which pack of cigarettes they would expect to deliver the least tar (or similar question variants) (see Additional file 5: Table S2), suggesting that exposure to ‘light’ , ‘mild’ and ‘ultra-light’ label descriptors decreased understanding of the content of the product (because ‘light’ or ‘mild’ cigarettes do not necessarily deliver less tar) [50].

## Discussion

### Summary of main results

This systematic review addressed the question “What do we know about the effects of exposure to ‘Low alcohol’ and equivalent product labelling on the amounts of alcohol, food and tobacco people select and consume?”. Little evidence was found that directly addressed this question (for example, only one study on alcohol labelling) – and the evidence that was available was overall of very low quality and equivocal. Nonetheless, the results of this review give some credence to the proposal that exposure to ‘Low alcohol’ and equivalent labelling on alcohol, food and tobacco products can shift consumer perceptions of labelled products.

This inferential claim is based on triangulation between three findings: (1) the results of a single, non-randomised controlled trial of alcohol product labels, that participants correctly understood the calorie and carbohydrate contents of the product to be lower when exposed to beer bottles labelled as ‘Light’; (2) evidence from randomised controlled trials of food product labels that participants understood products labelled with ‘Low fat’ or equivalent descriptors to contain fewer calories; and (3) evidence from non-randomised studies of tobacco product labels that participants believed cigarettes in packs displaying label descriptors such as ‘Light’ and ‘Mild’ to be less harmful to health and to deliver less tar (albeit incorrectly). Collectively, these findings indicate that labels of these kinds can alter adults’ perceptions concerning the content of products, and (with respect to food) what they judge to be an appropriate serving, with the potential to licence consumption of the labelled product.

However, the evidence in this review did not elucidate the likely sizes of these effects on consumers’ perceptions of the products. Moreover, equivocal results for behavioural endpoints (i.e. amounts of potentially health-harming substances selected and consumed) mean that the extent to which altered perceptions of products may result in behaviour change that protects or harms health remains uncertain. As such, the reviewed evidence sheds little light on this key public health question.

To compound the paucity of evidence for the effects of exposure to ‘Low alcohol’ and equivalent labels on alcohol products, the quality of evidence from studies of food and tobacco labelling was overall very low. For example, meta-analyses of outcome data from RCTs of food labelling ostensibly indicated that exposure to ‘Low fat’ or equivalent labels on food products increased participants’ understanding of the content of the product, but had no effect on quantities consumed, beliefs about consumption, or appeal. However, these results should be viewed with caution due to concerns about study limitations (unclear risk of bias), inconsistency, indirectness and imprecision.

### Implications for policy on lower strength alcohol labels

In the context of using label descriptors to denote lower strength alcohol products, consumers would understand a ‘Low alcohol’ label correctly if they perceived the labelled product to contain less alcohol and consequently to be less harmful compared with higher strength alternatives (assuming an equal portion size). Although evidence from studies of food and tobacco product labels differed in terms of whether exposure to ‘Low fat’ (food) or ‘Light’ (tobacco) labels was associated with correct or incorrect perceptions of this kind, included studies consistently found that adults exposed to such descriptors believed the labelled products to be *less* harmful to health, and/or to contain *less* of something (e.g. energy, fat, tar). Whilst the transferability of this finding to alcohol labelling has not been established by this review. it is at least plausible that people may apply similar mental shortcuts (heuristics and biases) when they see ‘Low alcohol’ and equivalent labels on lower strength alcohol products to those they apply upon seeing equivalent labels on food or tobacco products. However, consuming alcohol is also distinct from food or tobacco consumption in terms of its disinhibiting effect, which could moderate the ways product labels of this kind influence consumers’ awareness, perception and behaviour at the point of purchase and/or consumption. Also, the satiating effects experienced in the consumption of food and tobacco products do not apply in the same way to alcohol, and inebriation could interfere with information processing in ways that are not relevant to other products.

Whilst findings about the influences of these kinds of labels on consumers’ perceptions may represent a justifiable basis for policy development, there is currently insufficient evidence from research available in the public domain to inform choices between alternative descriptors, or about the types or strengths of alcoholic beverage products to which they should be applied; choices that clearly need to be addressed in formulating policy in this area. This limits the applicability of findings of this review to address policy makers’ current questions concerning potential differential impacts between specific labelling descriptors – both those currently in use and the wider range currently under consideration for alcohol labelling. Evidence from studies of ‘Low fat’ and equivalent labels on food products indicated that any effects of exposure to such labelling are likely to be inconsistent between label descriptors and specific products (albeit there were insufficient data to unpack differential effects using subgroup analyses). This suggests that the specific label descriptors used, and the specific products to which they are applied, would likely be critical determinants of the success of any moves to extend or restrict the use of such labels as a means of ‘nudging’ consumers to purchase and consume healthier food products, or less calories from food overall [51].

### Strengths and limitations of this review

A strength of this review is that it was conducted using systematic, prospective methods and procedures that aimed to minimise bias, so far as possible. There were, however, some limitations to the review. The search methods applied relied primarily on electronic keyword searches combined with snowball search techniques, as opposed to formal, protocol-driven searches of secondary electronic literature databases that have become standard in systematic reviews of interventions [5]. Formal, protocol-driven database searches were not judged feasible for this systematic review on pragmatic grounds. Specifically, search terms based on target intervention concepts (e.g. ‘label’ , ‘low’ , ‘light’ ‘reduced’ , ‘zero’ etc.) were not specific to titles, abstracts or index terms of the target set of eligible studies. Protocol-driven searches would therefore have had high sensitivity and low specificity, resulting in impractical screening workload, beyond available time and other resources. There is emerging evidence that less formal approaches, combined with snowball search techniques, can perform effectively as an alternative to protocol-driven searches in reviews of non-clinical interventions [52, 53]. We sought to mitigate any risk of selective sampling that may be introduced by omitting protocol-driven searches by conducting extensive backward and forward citation tracking from relevant reviews and eligible primary studies, which appeared to achieve saturation (with no new eligible studies being identified by the end of the second round of snowball searches). A second limitation is that contacting primary study authors for data missing from study reports was beyond the scope of available resources. Whilst it is possible that contacting authors may have helped assemble a more complete data set for analysis, it is highly unlikely that integration of these data would change the principal finding of this review – that current evidence is insufficient to reliably inform policy in this area.

## Conclusions

Considerable uncertainty remains about whether and how much amending or extending the range of alcohol strengths to which label descriptors denoting low strength can be applied might contribute to reducing alcohol consumption at population level. Whilst there is some indirect evidence suggesting that exposure to labelling of this kind on food and tobacco products can shift consumers’ perceptions of alcohol, food and tobacco products, corollary impacts on behaviour (amounts purchased, selected or consumed) were mixed and may vary between specific label descriptors, products and populations.

Appropriate labelling at the point of purchase, selection or consumption is perceived by policy-makers to be an important tool to facilitate more informed consumer choices, and looks set to continue to be a key component of public health strategies worldwide. Given the current lack of useful evidence to inform policy in this area, well designed and implemented, independent studies are urgently needed to inform potential changes to current labelling regulations, to help ensure future polices are based on high quality evidence. Such studies should be conducted in general adult samples, simulate real purchasing and consumption contexts (or use publicly accessible ‘real-time’ purchasing data), and aim to clarify the extent to which effects are modified by characteristics of products, label descriptors, and participants. They should also be designed to investigate the potential for such labels to increase, as well as to decrease, selection and consumption of alcohol and other potentially health-harming substances in alcohol, food and tobacco products.

## Additional files

Additional file 1: PRISMA statement. (DOC 62 kb)
Additional file 2: Bibliographies of studies. (DOCX 106 kb)
Additional file 3: Table S1. Characteristics and results of included randomised controlled trials. (DOCX 92 kb)
Additional file 4: Narrative description of key characteristics of included studies. (DOCX 118 kb)
Additional file 5: Table S2. Characteristics and results of included non-randomised studies. (DOCX 93 kb)
Additional file 6: Study-level risk of bias table. (DOCX 187 kb)
