# Supplementary material for: What do we know about the effects of exposure to ‘Low alcohol’ and equivalent product labelling on the amounts of alcohol, food and tobacco people select and consume? A systematic review
Source: BMC Public Health. 2017 Jan 12;17:29. doi: 10.1186/s12889-016-3956-2 (PMC5228109; doi:10.1186/s12889-016-3956-2)
Supplement: Additional file 2: — Bibliographies of studies. (DOCX 106 kb) [file 12889_2016_3956_MOESM2_ESM.docx]

**Additional file 2. Bibliographies of studies**

***Included studies***

*Alcohol product labels*

Bui, Burton, Howlett, & Kozup, 2008 [*Published data only*].

Bui M, Burton S, Howlett E, Kozup J. What am I drinking? The effects of serving facts information on alcohol beverage containers. J Consum Aff. 2008;42(1):81-99.

*Food product labels*

Aaron, Mela, & Evans, 1994 [*Published data only*].

Aaron JI, Mela DJ, Evans RE. The influences of attitudes, beliefs and label information on perceptions of reduced-fat spread. Appetite. 1994;22(1);25-37.

Bergen & Yeh, 2006 [*Published data only*].

Bergen D, Yeh MC. Effects of energy-content labels and motivational posters on sales of sugar-sweetened beverages: stimulating sales of diet drinks among adults study. J Am Diet Assoc. 2006;106(11):1866-69.

Crockett, Jebb, Hankins, & Marteau, 2014 [*Published data only*].

Crockett RA, Jebb SA, Hankins M, Marteau TM. The impact of nutritional labels and socioeconomic status on energy intake: an experimental field study. Appetite. 2014;81:12-19.

Dubbert, Johnson, Schlundt, & Montague, 1984 [*Published data only*].

Dubbert PM, Johnson WG, Schlundt DG, Montague NW. The influence of caloric information on cafeteria food choices. J Appl Behav Anal. 1984;17(1):85-92.

Ebneter, Latner, & Nigg, 2013 [*Published data only*].

Ebneter DS, Latner JD, Nigg CR. Is less always more? The effects of low-fat labeling and caloric information on food intake, calorie estimates, taste preference, and health attributions. Appetite. 2013;68:92-7.

French et al., 2001 [*Published data only*].

French SA, Jeffery RW, Story M, Breitlow KK, Baxter JS, Hannan P, et al. Pricing and promotion effects on low-fat vending snack purchases: the CHIPS Study. Am J Pub Health. 2001;91(1):112-7.

Kähkönen, Tuorila, & Rita, 1996 [*Published data only*].

Kähkönen P, Tuorila H, Rita H. How information enhances acceptability of a low-fat spread. Food Qual Prefer. 1996;7(2):87-94. [*Primary reference*]

Kähkönen P. Consumer acceptance of reduced-fat foods – the effects of product information. Helsinki: University of Helsinki; 2000.

Kähkönen, Tuorila, & Lawless, 1997 [*Published data only*].

Kähkönen P, Tuorila H, Lawless H. Lack of effect of taste and nutrition claims on sensory and hedonic responses to a fat-free yogurt. Food Qual Prefer. 1997;8(2):125-30. [*Primary reference*]

Kähkönen P. Consumer acceptance of reduced-fat foods – the effects of product information. Helsinki: University of Helsinki; 2000.

Kähkönen & Tuorila, 1998 [*Published data only*].

Kähkönen P, Tuorila H. Effect of reduced-fat information on expected and actual hedonic and sensory ratings of sausage. Appetite. 1998;30(1):13-23. [*Primary reference*]

Kähkönen P. Consumer acceptance of reduced-fat foods – the effects of product information. Helsinki: University of Helsinki; 2000.

Kähkönen, Hakanpää, & Tuorila, 1999a [*Published data only*].

Kähkönen P, Hakanpää P, Tuorila H. The effect of information related to fat content and taste on consumer responses to a reduced-fat frankfurter and a reduced-fat chocolate bar. *J Sens Stud*. 1999a;14(1):35-46. [*Primary reference*]

Kähkönen P. Consumer acceptance of reduced-fat foods – the effects of product information. Helsinki: University of Helsinki; 2000.

Kiesel & Villas-Boas, 2013 [*Published data only*].

Kiesel K, Villas-Boas SB. Can information costs affect consumer choice? Nutritional labels in a supermarket experiment. Int J Ind Organ. 2013;31(2):153-63.

Kruja, 2014 [*Published data only*].

Kruja B. I’ll drink to that: Differential effect of fat labeling, weight salience and dietary restraint on consumption. Honors Theses, Paper 97. Williamsburg VA: College of William and Mary; 2014.

Liem, Toraman Aydin, & Zandstra, 2012 [*Published data only*].

Liem DG, Toraman Aydin N, Zandstra EH. Effects of health labels on expected and actual taste perception of soup. Food Qual Prefer. 2012;25(2):192-97.

Norton, Fryer, & Parkinson, 2013 [*Published data only*].

Norton JE, Fryer PJ, Parkinson JA. The effect of reduced-fat labelling on chocolate expectations. Food Qual Prefer. 2013;28(1):101-5.

Stubenitsky, Aaron, Catt, & Mela, 1999 [*Published data only*].

Stubenitsky K, Aaron JI, Catt SL, Mela DJ. Effect of information and extended use on the acceptance of reduced-fat products. Food Qual Prefer. 1999;10(4–5):367.

Wansink & Chandon 2006 (S2) [*Published data only*].

Wansink B, Chandon P. Can 'low-fat' nutrition labels lead to obesity? J Marketing Res. 2006;43(4):605-17. [*Primary reference*]

Wansink B, Chandon P. Health halos: How nutrition claims influence food consumption for overweight and normal weight people. INSTEAD Working Paper Series: 2005/73/MKT*.* Fontainebleau: INSTEAD; 2005.

Wansink & Chandon 2006 (S3) [*Published data only*].

Wansink B, Chandon P. Can 'low-fat' nutrition labels lead to obesity? J Marketing Res. 2006;43(4):605-17. [*Primary reference*]

B, Chandon P. Health halos: How nutrition claims influence food consumption for overweight and normal weight people. INSTEAD Working Paper Series: 2005/73/MKT*.* Fontainebleau: INSTEAD; 2005.

Wardle & Solomons, 1994 [*Published data only*].

Wardle J, Solomons W. Naughty but nice: a laboratory study of health information and food preferences in a community sample. Health Psychol, 1994;13(2):180-3.

Westcombe & Wardle, 1997 [*Published data only*].

Westcombe A, Wardle J. Influence of relative fat content information on responses to three foods. Appetite. 1997;28(1):49-62.

*Tobacco product labels*

Bansal-Travers, Hammond, Smith, & Cummings, 2011 [*Published data only*].

Bansal-Travers M, Hammond D, Smith P, Cummings KM. The impact of cigarette pack design, descriptors, and warning labels on risk perception in the U.S. Am J Prev Med. 2011;40(6):674-82.

Cohen, Yang, & Donaldson, 2014 [*Published data only*].

Cohen JE, Yang J, Donaldson EA. Impact of the removal of light and mild descriptors from cigarette packages in Ontario, Canada: switching to 'light replacement' brand variants. Prev Med. 2014;69(0):120-5.

Hammond, Dockrell, Arnott, Lee, & McNeill, 2009 [*Published data only*].

Hammond D, Dockrell M, Arnott D, Lee A, McNeill A. Cigarette pack design and perceptions of risk among UK adults and youth. Eur J Public Health, 2009;19(6):631-7.

Hammond & Parkinson, 2009 [*Published data only*].

Hammond D, Parkinson C. The impact of cigarette package design on perceptions of risk. J Public Health. 2009;31(3):345-53.

Siahpush et al., 2011 [*Published data only*].

Siahpush M, Borland R, Fong GT, Elton-Marshall T, Yong HH, Holumyong C. Socioeconomic differences in the effectiveness of the removal of the 'light' descriptor on cigarette packs: findings from the International Tobacco Control (ITC) Thailand Survey. Int J Environ Res Public Health, 2011;8(6):2170-80.

Yong et al., 2011 [*Published data only*].

Yong HH, Borland R, Cummings KM, Hammond D, O'Connor RJ, Hastings, G, King B. Impact of the removal of misleading terms on cigarette pack on smokers' beliefs about 'light/mild' cigarettes: cross-country comparisons. Addiction. 2011;106(12):2204-13.

***Excluded full-text study reports***

Albright CL, Flora JA, Fortmann SP. Restaurant menu labeling: impact of nutrition information on entree sales and patron attitudes. Health Educ Quart. 1990;17(2):157-67.

Ashley MJ, Cohen J, Ferrence R. 'Light' and 'mild' cigarettes: who smokes them? Are they being misled? Can J Public Health. 2001;92(6):407-11.

Balasubramanian SK. Consumers’ search and use of nutrition information: The challenge and promise of the Nutrition Labeling and Education Act. J Marketing. 2002;66(3):112-27.

Berning JP, Chouinard HH, McCluskey JJ. Do positive nutrition shelf labels affect consumer behavior? Findings from a field experiment with scanner data. Am J Agr Econ. 2010;93(2):364-9.

Bowen D, Green P, Vizenor N, Vu C, Kreuter P, Rolls B. Effects of fat content on fat hedonics: cognition or taste? Physiol Behav. 2003;78(2):247-53.

Bowen DJ, Tomoyasu N, Anderson M, Carney M, Kristal A. Effects of expectancies and personalized feedback on fat consumption, taste, and preference. J Appl Soc Psychol. 1992;22(13):1061-79.

Brown A, McNeill A, Mons U, Guignard R. Do smokers in Europe think all cigarettes are equally harmful? Eur J Public Health. 2012;22(S1):35-40.

Buscher LA, Martin KA, Crocker S. Point-of-purchase messages framed in terms of cost, convenience, taste, and energy improve healthful snack selection in a college foodservice setting. J Am Diet Assoc. 2001;101(8):909-13.

Bushman BJ. Effects of warning and information labels on consumption of full-fat, reduced-fat, and no-fat products. J Appl Psychol. 1998;83(1):97-101.

Chaudhary R. Determinants influencing consumer purchase decision with reference to 'lite' products. International Proceedings of Economics Development and Research. 2013;57.

Chrysochou P. Drink to get drunk or stay healthy? Exploring consumers’ perceptions, motives and preferences for light beer. Food Qual Prefer. 2014;31(0):156-63.

Chu YH, Jones SJ, Frongillo EA, DiPietro RB, Thrasher JF. Investigating the impact of menu labeling on revenue and profit in a foodservice operation. Journal of Foodservice Business Research. 2014;17(3):215-27.

Cinciripini PM. Changing food selections in a public cafeteria an applied behavior analysis. Behav Modifi. 1984;8(4):520-39.

Connolly GN, Alpert HR. (Has the tobacco industry evaded the FDA's ban on 'light' cigarette descriptors? Tob Control. 2014;23(2):140-45.

Daillant-Spinnler B, Issanchou S. Influence of label and location of testing on acceptability of cream cheese varying in fat content. Appetite. 1995;24(2):101-5.

Davis-Chervin D, Rogers T, Clark M. Influencing food selection with point-of-choice nutrition information. J Nutr Educ. 1985;17(1):18-22.

Dean M, Shepherd R, Arvola A, Vassallo M, Winkelmann M, Claupein E, et al. Consumer perceptions of healthy cereal products and production methods. J Cereal Sci. 2007;46(3):188-96.

Di Monaco R, Ollila S, Tuorila H. Effect of price on pleasantness ratings and use intentions for a chocolate bar in the presence and absence of a health claim. J Sens Stud. 2005;20(1):1-16.

Doxey J, Hammond D. Deadly in pink: the impact of cigarette packaging among young women. Tob Control. 2011;20(5):353-60.

Elton-Marshall T, Fong GT, Yong H-H, Borland R, Xu SS, Quah ACK, et al. Smokers’ sensory beliefs mediate the relation between smoking a ‘light/low tar’ cigarette and perceptions of harm. Tob Control. 2014; doi:10.1136/tobaccocontrol-2014-051977.

Elton-Marshall T, Fong GT, Zanna MP, Jiang Y, Hammond D, O'Connor RJ, et al. Beliefs about the relative harm of 'light' and 'low tar' cigarettes: findings from the International Tobacco Control (ITC) China Survey. Tob Control. 2010;19(S2):i54-i62.

Engell D, Bordi P, Borja M, Lambert C, Rolls B. Effects of information about fat content on food preferences in pre-adolescent children. Appetite. 1998;30(3):269-82.

Ernst N, Wu M, Frommer P, Katz E, Matthews O, Moskowitz J, et al. Nutrition education at the point of purchase: the foods for health project evaluated. Prev Med. 1986;15(1):60-73.

Faulkner GP, Pourshahidi LK, Wallace JMW, Kerr MA, McCaffrey TA, Livingstone MBE. Perceived 'healthiness' of foods can influence consumers' estimations of energy density and appropriate portion size. Int J Obesity. 2014;38(1):106-12.

Finkelstein SR, Fishbach A. When healthy food makes you hungry. J Consum Res. 2010;37(3):357-67.

Fiske A, Cullen KW. Effects of promotional materials on vending sales of low-fat items in teachers' lounges. J Am Diet Assoc. 2004;104(1):90-3.

Fitzpatrick MP, Chapman GE, Barr SI. Lower-fat menu items in restaurants satisfy customers. J Am Diet Assoc. 1997;97(5):510-14.

Girz L, Polivy J, Herman CP, Lee H. The effects of calorie information on food selection and intake. Int J Obesity. 2012;36(10):1340-5.

Gorton D, Mhurchu CN, Bramley D, Dixon R. Interpretation of two nutrition content claims: a New Zealand survey. Aust NZ J Publ Heal. 2010;34(1):57-62.

Gravel K, Doucet E, Herman CP, Pomerleau S, Bourlaud AS, Provencher V. 'Healthy', 'diet', or 'hedonic'. How nutrition claims affect food-related perceptions and intake? Appetite. 2012;59(3):877-84.

Gray JP, Karnon J, Blackwell L. Sugar consumption from beverages and the potential effects of a text-based information label. Aust NZ J Publ Heal. 2011;35(1):88-9.

Guinard J-X, Marty C. Acceptability of fat-modified foods to children, adolescents and their parents: effect of sensory properties, nutritional information and price. Food Qual Prefer. 1997;8(3):223-31.

Guinard J-X, Smiciklas-Wright H, Marty C, Sabha RA, Soucy I, Taylor-Davis S, et al. Acceptability of fat-modified foods in a population of older adults: contrast between sensory preference and purchase intent. Food Qual Prefer. 1996;7(1):21-8.

Hammond D, Daniel S, White CM. The effect of cigarette branding and plain packaging on female youth in the United Kingdom. J Adolescent Health. 2013;52(2):151-7.

Hoek J, Wong C, Gendall P, Louviere J, Cong K. Effects of dissuasive packaging on young adult smokers. Tob Control. 2011;20(3):183-8.

Hoerr SM, Louden VA. Can nutrition information increase sales of healthful vended snacks? J School Health.1993;63(9):386-90.

Horgen KB, Brownell KD. Comparison of price change and health message interventions in promoting healthy food choices. Health Psychol. 2002;21(5):505-12.

Jeffery RW, Pirie PL, Rosenthal BS, Gerber WM, Murray DM. Nutrition education in supermarkets: an unsuccessful attempt to influence knowledge and product sales. J Behav Med. 1982;5(2):189-200.

Johansen SB, Næs T, Øyaas J, Hersleth M. Acceptance of calorie-reduced yoghurt: effects of sensory characteristics and product information. Food Qual Prefer. 2010;21(1):13-21.

Kähkönen P, Tuorila H. Consumer responses to reduced and regular fat content in different products: effects of gender, involvement and health concern. Food Qual Prefer. 1999b;10(2):83-91.

Kocken PL, Eeuwijk J, Van Kesteren NM, Dusseldorp E, Buijs G, Bassa-Dafesh Z, et al. Promoting the purchase of low-calorie foods from school vending machines: a cluster-randomized controlled study. J School Health. 2012;82(3):115-22.

Liem DG, Miremadi, F, Zandstra EH, Keast RS. Health labelling can influence taste perception and use of table salt for reduced-sodium products. Public Health Nutr. 2012;15(12):2340-7.

Masson J, Aurier P. Effects of non-sensory cues on perceived quality: the case of low-alcohol wine. Int J Wine Bus Res. 2008;20(3):215-29.

Mayer JA, Brown TP, Heins JM, Bishop DB. A multi-component intervention for modifying food selections in a worksite cafeteria. J Nutr Educ. 1987;19(6):277-80.

Mayer JA, Heins JM, Vogel JM, Morrison DC, Lankester LD, Jacobs AL. Promoting low‐fat entree choices in a public cafeteria. J Appl Behav Anal. 1986;19(4):397-402.

McCann MT, Wallace JM, Robson PJ, Rennie KL, McCaffrey TA, Welch RW, et al. Influence of nutrition labelling on food portion size consumption. Appetite. 2013;65:153-8.

Mela DJ, Trunck F, Aaron JI. No effect of extended home use on liking for sensory characteristics of reduced-fat foods. Appetite. 1993;21(2):117-29.

Miller DL, Castellanos VH, Shide DJ, Peters JC, Rolls BJ. Effect of fat-free potato chips with and without nutrition labels on fat and energy intakes. Am J Clin Nutr. 1996;68(2):282-90.

Ng J, Stice E, Yokum S, Bohon C. An fMRI study of obesity, food reward, and perceived caloric density. Does a low-fat label make food less appealing? Appetite. 2011;57(1):65-72.

Northup T. Truth, lies, and packaging: how food marketing creates a false sense of health. Food Studies. 2014;9.

Perlmutter CA, Canter DD, Gregoire MB. Profitability and acceptability of fat- and sodium-modified hot entrees in a worksite cafeteria. J Am Diet Assoc. 1997;97(4):391-395.

Provencher V, Polivy J, Herman CP. Perceived healthiness of food. If it's healthy, you can eat more! Appetite. 2009;52(2):340-4.

Roefs, A, Jansen A. The effect of information about fat content on food consumption in overweight/obese and lean people. Appetite. 2004;43(3):319-322.

Russo JE, Staelin R, Nolan C A, Russell GJ, Metcalf BL. Nutrition information in the supermarket. J Consum Res. 1986;13(1):48-70.

Shide DJ, Rolls BJ. Information about the fat content of preloads influences energy intake in healthy women. J Am Diet Assoc.1995;95(9):993-8.

Stubenitsky K, Aaron J, Catt S, Mela D. The influence of recipe modification and nutritional information on restaurant food acceptance and macronutrient intakes. Public Health Nutr. 2000;3(02):201-9.

Wagner JL, Winett RA. Prompting one low‐fat, high‐fiber selection in a fast‐food restaurant. J Appl Behav Anal. 1988;21(2):179-85.

Wansink B, van Ittersum K, Painter JE. How diet and health labels influence taste and satiation. J Food Sci. 2004;69(9):S340-S346.

Webb KL, Solomon LS, Sanders J, Akiyama C, Crawford PB. Menu labeling responsive to consumer concerns and shows promise for changing patron purchases. J Hunger Environ Nutr. 2011;6(2):166-78.

Wilbur CS, Zifferblatt SM, Pinsky JL, Zifferblatt S. Healthy vending: a cooperative pilot research program to stimulate good health in the marketplace. Prev Med. 1981;10(1):85-93.

Yeomans MR, Lartamo S, Procter EL, Lee MD, Gray RW. The actual, but not labelled, fat content of a soup preload alters short-term appetite in healthy men. Physiol Behav. 2001;73(4):533-40.

***Identified relevant reviews***

Crockett RA, Hollands GJ, Jebb SA, Marteau TM. Nutritional labelling for promoting healthier food purchasing and consumption. Cochrane Database of Systematic Reviews. 2011;9.

Escaron AL, Meinen AM, Nitzke SA, Martinez-Donate AP. Supermarket and Grocery Store–Based Interventions to Promote Healthful Food Choices and Eating Practices: A Systematic Review. Preventing Chronic Disease. 2013;10:120-56.

Glanz K, Hewitt AM, Rudd J. Consumer behavior and nutrition education: An integrative review. J Nutr Educ. 1992;24(5):267-77.

Hammond D. Tobacco packaging and labelling: a review of evidence. Waterloo, ON: Department of Health Studies, University of Waterloo; 2007.

Jones L, Bellis MA. Can promotion of lower strength alcohol products help reduce alcohol consumption? A rapid literature review. Liverpool: North West Public Health Observatory, Liverpool John Moores University; 2012.

Kozlowski LT, Pillitteri JL. Beliefs about 'Light' and 'Ultra Light' cigarettes and efforts to change those beliefs: an overview of early efforts and published research. Tob Control. 2001;10(S1):i12-i16.

Liberato S, Bailie R, Brimblecombe J. Nutrition interventions at point-of-sale to encourage healthier food purchasing: a systematic review. BMC Public Health. 2014;4(1):919.

Mayer JA, Dubbert PM, Elder JP. Promoting Nutrition at the Point of Choice: A Review. Health Educ Behav. 1989;16(1):31-43.

McNeill A, Bauld, L, Birken M, Hammond D, Moodie C, Stead M, et al. Tobacco packaging design for preventing tobacco uptake. Cochrane Database of Systematic Reviews. 2014;8.

Spence C, Piqueras-Fiszman B. The perfect meal: the multisensory science of food and dining. Oxford: Wiley-Blackwell; 2014.

Stead M, Moodie C, Angus K, Bauld L, McNeill A, Thomas J, et al. Is consumer response to plain/standardised tobacco packaging consistent with framework convention on tobacco control guidelines? A systematic review of quantitative studies. PLoS One. 2013;8(10):e75919.

van 't Riet J. Sales effects of product health information at points of purchase: a systematic review. Public Health Nutr. 2013;16(3):418-29.

Wilkinson C, Room R. Warnings on alcohol containers and advertisements: international experience and evidence on effects. Drug Alcohol Rev. 2009;28(4):426-35.
