# Supplementary material for: What do we know about the effects of exposure to ‘Low alcohol’ and equivalent product labelling on the amounts of alcohol, food and tobacco people select and consume? A systematic review
Source: BMC Public Health. 2017 Jan 12;17:29. doi: 10.1186/s12889-016-3956-2 (PMC5228109; doi:10.1186/s12889-016-3956-2)
Supplement: Additional file 3: Table S1. — Characteristics and results of included randomised controlled trials. (DOCX 92 kb) [file 12889_2016_3956_MOESM3_ESM.docx]

**Additional file 3: Table S1 Characteristics and results of included randomised controlled trials (N=12).**

| *Study* | *Funding source* | *Design* | *Country, setting* | *Participants that completed study* | *Intervention(s)* | *Comparator(s)* | *Outcome measures* | *Effect of exposure to low label* | *Study-level effect size (SMD and 95% CI)* |
| --- | --- | --- | --- | --- | --- | --- | --- | --- | --- |
| Aaron, Mela, & Evans, 1994 [19] | Funding source (if any) is not reported. | I-RCT-C | UK, Laboratory (a purpose-built sensory evaluation laboratory, under dim red-light). | 101 local consumers; age (M=40.5yrs, SD=NR);  69% female; 41% restrained eaters (DEBQ). | Exposure to a tray containing a china plate, a piece of medium sliced white bread, a pre-weighed stainless steel knife and a clear glass bowl of reduced-fat spread of 39.6% fat∱ and a label containing the descriptor ‘Reduced-fat margarine (40% fat)’ attached to the tray¶. | Exposure to a tray containing a china plate, a piece of medium sliced white bread, a pre-weighed stainless steel knife and a clear glass bowl of reduced-fat spread of 39.6% fat∱ and a label containing the descriptor ‘Full-fat margarine (80% fat)’ attached to the tray¶. | *Selection:-*  Amount of spread selected (grams).  *Appeal:-*  Flavour (100mm VAS anchored ‘poor’ and ‘excellent’*).  Texture*.  Smoothness*.  Mouthfeel*.  Overall liking (Classic pleasantness scale, 9-point scale ‘dislike extremely’ to ‘like extremely’).  Overall liking (Food Action Scale, 9-point scale ‘I would eat this if I were forced to’ to ‘I would eat this every opportunity I had’). | ↑  →←  →←  →←  →←  →←  →← | 0.31 (0.04 to 0.58)  0.17 (-0.10 to 0.44) |
| Crockett, Jebb, Hankins, & Marteau, 2014 [24] | National Institute for Health Research, UK, Postdoctoral Fellowship award (PDF-2009-02-14). | I-RCT-PG | UK, Real-world micro-environment (a cinema auditorium). | 287 adults§; aged ≥ 18 years; 64% female. | Exposure to a tub of salted or toffee popcorn∱ labelled with the descriptor ‘Low fat’.  [+ *Exposure to green colour coding on the label*] | Exposure to a tub of salted or toffee popcorn∱ with no equivalent labelling. | *Consumption:-*  Total energy consumed from popcorn (kcals).  *Appeal:-*  Taste of the snack (5 items assessed the extent to which participants rated the popcorn as good-tasting, strong tasting and unpleasant tasting). | →←  NR | -0.20 (-0.48 to 0.08)  No useable data. |
| Ebneter, Latner, & Nigg, 2013 [26] | Funding source (if any) is not reported. | I-RCT-PG | USA, Unclear (not described). | 87 undergraduate students;  age (M=20.5yrs, SD=3.4);  100% female. | Exposure to a glass pitcher containing 2,530g of M&M’s∱ (teal, silver, and gold coloured) and a 6.5x4.5 inch place card containing the text ‘‘New Colors of Low-Fat M&M’s’¶. | Exposure to a glass pitcher containing 2,530g of M&M’s∱ (teal, silver, and gold coloured) and a 6.5x4.5 inch place card containing the text ‘‘New Colors of Regular M&M’s’¶. | *Consumption:-*  Amount of M&Ms consumed (kcals).  *Appeal:-*  ‘‘How good did this snack taste to you?’’ (5-point scale ‘Not at all’ to ‘Very’).  *Understanding of Label:-*  ‘‘How many calories do you think are in one serving size (1.69 oz) of the M&M’s that you tried? One serving size equals 55 M&M’s.’’  ‘‘How healthy is this snack that you tried for you?’’ (5-point scale ‘very unhealthy’ to ‘very healthy’).  ‘‘If you were eating this snack regularly, how would it affect your weight?’’ (5-point scale ‘I would lose a lot of weight’ to ‘I would gain a lot of weight’).  ‘‘Do you think this snack would belong in a healthy diet?’’ (5-point scale ‘would belong very well’ to ‘would not belong at all’). | →←  →←  ↑  ↑  →←  →← | -0.18 (-0.24 to 0.60)  0.23 (-0.19 to 0.65)  -0.69 (-1.12 to -0.26) |
| French et al., 2001 [27] | National Institutes of Health, USA, Grant (RO1 HL56577). | C-RCT-C | USA, Real-world micro-environment (55 vending machines in 12 schools and worksites). | NR consumers;  age NR;  NR% female. | Exposure to vending machines containing snack products that included low-fat snack products∱ with labels placed on the vending machines labelling the products as low fat (precise wording NR)¶.  [+ *Exposure to price information on the labels*] | Exposure to vending machines containing snack products that included low-fat snack products∱ with no equivalent labelling.  [+ *Exposure to price information on the labels*] | *Selection:-*  Proportion of snack items sold classified as low-fat items (% based on average sales per site per experimental period).  Absolute number of low-fat snack items sold (N based on average sales per site per experimental period). | →←  →← | No useable data. |
| Kähkönen, Tuorila, & Lawless, 1997 [31-32] | Funding source (if any) is not reported. | I-RCT-PG | Finland, Real-world micro-environment (3 worksite cafeterias). | 156 adult employees;  age NR;  56% female; 44% lower SES (Education: Low – unspecified). | Exposure to a white 125 ml plastic cup covered with an aluminium lid, containing 0.5% fat strawberry yogurt∱, labelled with a cover sheet over the question form printed with large font text ‘The sample you are going to rate is a fat-free strawberry yogurt’¶. | Exposure to a white 125 ml plastic cup covered with an aluminium lid, containing 0.5% fat strawberry yogurt∱, labelled with a cover sheet over the question form printed with large font text ‘The sample you are going to rate is a strawberry yogurt’¶. | *Appeal:-*  Pleasantness (9-point scale ‘extremely unpleasant’ to ‘extremely pleasant’).  Sweetness (9-point scale ‘not at all sweet’ to ‘extremely sweet’).  Sourness (9-point scale ‘not at all sour' to ‘extremely sour’)  Thickness (9-point scale ‘thin’ to ‘thick’).  Smoothness of texture (9-point scale ‘not at all smooth’ to ‘smooth’). | →← | -1.0 (-0.41 to 0.21) |
| Kähkönen, Hakanpää, & Tuorila, 1999a. [31, 34] | Academy of Finland (Project 33251) and the EU (FAIR CT95-0574). | I-RCT-PG | Finland, Laboratory (individual tasting booths). | 91 university employees or students§; age (M=31.0yrs, SD=NR);  68% female. | Exposure to a bar of chocolate∱ and a photograph of the same bar of chocolate in its packaging with the label descriptor ‘Chocolate - Reduced-fat’¶.  Exposure to a frankfurter sausage∱ and a photograph of its packaging with the label descriptor ‘Frankfurter - Reduced-fat’¶. | Exposure to a bar of chocolate∱ and a photograph of the same bar of chocolate in its packaging with the label descriptor ‘Chocolate’¶.  Exposure to a frankfurter sausage∱ and a photograph of its packaging with the label descriptor ‘Frankfurter’¶. | *Appeal:-*  Pleasantness (9-point scale ‘extremely unpleasant’ to ‘extremely pleasant’).  Flavour intensity (9-point scale ‘mild chocolate flavor’ to ‘strong chocolate flavor’).  Rate of melting in the mouth (9-point scale ‘melts slowly’ to ‘melts quickly’).  Fattiness (9-point scale ‘not at all fatty’ to ‘extremely fatty’).  *Appeal:-*  Pleasantness (9-point scale ‘extremely unpleasant’ to ‘extremely pleasant’).  Flavour intensity (9-point scale ‘mild flavour’ to ‘strong flavour’).  Juiciness (9-point scale ‘not at all juicy’ to ‘extremely juicy’  Fattiness (9-point scale ‘not at all fatty’ to ‘extremely fatty’). | →←  NRA  NRA  NRA  →←  NRA  NRA  NRA | -0.32 (-1.02 to 0.38)  0.24 (-0.46 to 0.94) |
| Kruja, 2014 [36]. | Funding source (if any) is not reported. | I-RCT-C | USA, Laboratory. | 128 undergraduate students; age (M=19.5yrs, SD=1.7);  100% female; 54% restrained eaters (RS). | Exposure to a red Solo cup® containing 275 g Nesquick® calcium fortified low-fat chocolate milk∱ labelled with a ‘Fat Free’ descriptor. | Exposure to a red Solo cup® containing 275 g Nesquick® calcium fortified low-fat chocolate milk∱ labelled with a ‘Full fat’ descriptor. | *Consumption:-*  Amount of drink consumed (grams).  *Appeal:-*  Overall liking (7-point scale ‘Strongly Dislike’ to ‘Strongly Like’*).  Taste*.  Smell*.  Flavor*.  Visual appeal*.  *Understanding of the label:-*  Number of calories by which content of one serving of chocolate milk was under- or overestimated (kcals).  Volume by which content of one serving of chocolate milk was under- or overestimated (millilitres). | ↑  →←  NRA  NRA  NRA  NRA  →←  →← | 0.66 (0.41 to 0.91)  0.04 (-0.20 to 0.28)  No useable data  No useable data |
| Norton, Fryer, & Parkinson, 2013 [38]. | Engineering and Physical Sciences Research Council, UK. | I-RCT-C | UK, Laboratory. | 87 university postgraduate or undergraduate students or staff; age (M=24.3yrs, SD=9.6);  72% female. | Exposure to a plain white ceramic plate containing three unbroken squares of supermarket own-brand milk chocolate∱ (6.59 g, SD = 0.57) and a 18x6cm label containing the text ‘Sample 276: Reduced-Fat Milk Chocolate’ placed on the plate.¶ | Exposure to a plain white ceramic plate containing three unbroken squares of supermarket own-brand milk chocolate∱ (6.59 g, SD = 0.57) and a 18x6cm label containing the text ‘Sample 530: Milk Chocolate’ placed on the plate.¶ | *Product appeal:-*  Overall liking, ‘How much do you like the chocolate overall?’ (VAS, anchored ‘Like not at all’ and ‘Like Extremely’*).  Anticipated liking, ‘How much do you think you’ll like this chocolate?’*. | →←  ↓ | -0.09 (-0.38 to 0.20) |
| Stubenitsky, Aaron, Catt, & Mela, 1999 [40] | European Commission (Project FAIR-CT95-0574) and the Bio-technology & Biological Sciences Research Council. | I-RCT-PG | UK, Real-world micro-environment (participants’ homes). | 71 consumers; age (M= 38.6yrs, SD=11.3);  75% female. | Exposure to packs of reduced fat pork sausages∱ for at-home consumption that were labelled with the generic product name and the descriptor ‘Reduced-fat’ (i.e. ‘Reduced-fat pork sausages’).  Exposure to packs of reduced fat milk chocolate snack bars∱  for at-home consumption that were labelled with the generic product name and the descriptor ‘Reduced-fat’ (i.e. ‘Reduced-fat milk chocolate snack bar’). | Exposure to packs of reduced fat pork sausages∱ for at-home consumption that were labelled with the generic product name only (i.e. ‘Pork sausages’).  Exposure to packs of reduced fat milk chocolate snack bars∱ for at-home consumption that were labelled with the generic product name only (i.e. ‘Milk chocolate snack bar’). | *Belief associated with consumption:-*  Fillingness (100mm VAS anchored ‘not at all filling' and ‘extremely filling').  *Appeal:-*  Pleasantness (100mm VAS anchored ‘extremely unpleasant' and ‘extremely pleasant').  Overall idealness (100mm VAS anchored ‘not at all my ideal sausage' and ‘exactly my ideal sausage').  Boredom (100mm VAS anchored ‘not at all bored' and ‘extremely bored').  *Belief associated with consumption:-*  Fillingness (100mm VAS anchored ‘not at all filling' and ‘extremely filling').  *Appeal:-*  Pleasantness (100mm VAS anchored ‘extremely unpleasant' and ‘extremely pleasant').  Overall idealness (100mm VAS anchored ‘not at all my ideal sausage' and ‘exactly my ideal sausage').  Boredom (100mm VAS anchored ‘not at all bored' and ‘extremely bored'). | NR  →←  →←  →←  NR  ↓  ↓  ↓ | No useable data.  No useable data.  No useable data.  No useable data. |
| Wansink & Chandon 2006 (S2) [41, 42] | Funding source (if any) is not reported. | I-RCT-PG | USA, Laboratory. | 74 adults§;  age (M= 38.8yrs, SD=NR); 56% female; 24% overweight or obese. | Exposure to a transparent measuring cup containing 10 ounces of M&M’s and a label with the text ‘Low-Fat M&M’s’.  Exposure to a transparent measuring cup containing 10 ounces of regular granola and a label with the text ‘Low-Fat Granola’. | Exposure to a transparent measuring cup containing 10 ounces of M&M’s and a label with the text ‘Regular M&M’s’.  Exposure to a transparent measuring cup containing 10 ounces of regular granola and a label with the text ‘Regular Granola’. | *Belief associated with consumption:-*  Anticipated consumption guilt ‘How guilty would you feel after consuming  2oz of this snack?’ (9-point scale, NR).  *Understanding of label:-*  Perceived calorie density (Participants ‘asked to estimate the total number of calories contained in each measuring cup’).  Perceived serving size (Participants were ‘asked to evaluate serving sizes by estimating (1) the number of ounces of the snack that would be appropriate for a typical person to eat during a 90-minute movie and (2) the number of ounces that would be appropriate for them to eat in the same situation. To help them calibrate their estimates, there were ounce markings on the side of each measuring cup.’).  *Belief associated with consumption:-*  Anticipated consumption guilt ‘How guilty would you feel after consuming  2oz of this snack?’ (9-point scale, NR).  *Understanding of label:-*  Perceived calorie density (Participants ‘asked to estimate the total number of calories contained in each measuring cup’).  Perceived serving size (Participants were ‘asked to evaluate serving sizes by estimating (1) the number of ounces of the snack that would be appropriate for a typical person to eat during a 90-minute movie and (2) the number of ounces that would be appropriate for them to eat in the same situation. To help them calibrate their estimates, there were ounce markings on the side of each measuring cup.’). | →←  ↑  ↑  →←  ↑  ↑ | -0.14 (-0.79 to 0.51)  -0.37 (-1.03 to 0.29)  -0.66 (-1.32 to 0.00)  -0.51 (-1.17 to 0.15) |
| Wansink & Chandon 2006 (S3) [41, 42]. | Funding source (if any) is not reported. | I-RCT-PG | USA, Real-world micro-environment (a cinema auditorium). | 179 adults§;  age NR; NR% female; 39% overweight or obese. | 640 calories of granola∱ in a zip-lock bag with a 3.25” × 4” colour label attached containing the text ‘Low-Fat Rocky Mountain Granola’. | 640 calories of granola∱ in a zip-lock bag with a 3.25” × 4” colour label attached containing the text ‘Regular Rocky Mountain Granola’. | *Consumption:-*  Total energy consumed from granola (kcals).  *Understanding of the label:-*  Perceived number of servings contained in the bag (number). | ↑  ↓ | 0.69 (0.20 to 1.18)  -0.25 (-0.39 to -0.11) |
| Wardle & Solomons, 1994 [43]. | Funding source (if any) is not reported. | I-RCT-C | UK, Laboratory. | 40 university technical, administrative or research staff;  age (M= 35.5yrs, SD=9.8); 50% female. | Exposure to cheese spread sandwiches∱ presented on a plate labelled with the descriptor ‘Low-fat’¶.  [+ *Exposure to a leaflet containing information about heart disease prior to exposure to label condition*] | Exposure to cheese spread sandwiches∱ presented on a plate labelled with the descriptor ‘Full-fat’¶.  [+ *Exposure to a leaflet containing information about heart disease prior to exposure to label condition*] | *Consumption:-*  Number of sandwiches eaten (number).  *Appeal:-*  Overall liking of foods (10-point scale ‘strong dislike’ to ‘strong liking’). | →←  →← | 0.05 (-0.38 to 0.48)  -0.41 (-0.85 to 0.03) |

I-RCT-C: Individually randomised controlled trial with a crossover (within-subjects) design.

I-RCT-PG: Individually randomised controlled trial with a parallel group (between-subjects) design.

C-RCT-C: Cluster randomised controlled trial with a crossover (within-subjects) design.

NR: Not reported.

NRA: Not readily accessible.

DEBQ: Dutch Eating Behaviour Questionnaire [49].

RS: Restraint Scale [50]

VAS: Visual analogue scale.
§ Data are not specific to eligible within-study comparison(s).

¶ Label placed adjacent to the product or its packaging rather than placed on, or incorporated into, the product or its packaging.

∱ Exposed participants tasted or consumed product as part of the study procedure.

[+ *Exposure to…*]: Description of concurrent intervention.

* Measured using same instrument.

→← Estimated effect of ‘low fat’ or equivalent label is consistent with no difference (based on study-level effect size if estimable or else on study report results/discussion).

↑ Estimated effect of ‘low fat’ or equivalent label is consistent with an increase, e.g. more consumption, higher appeal (based on study-level effect size if estimable or else on study report results/discussion).

↓ Estimated effect of ‘low fat’ or equivalent label is consistent with a decrease, e.g. less consumption, lower appeal (based on study-level effect size if estimable or else on study report results/discussion).
