# Supplementary material for: What do we know about the effects of exposure to ‘Low alcohol’ and equivalent product labelling on the amounts of alcohol, food and tobacco people select and consume? A systematic review
Source: BMC Public Health. 2017 Jan 12;17:29. doi: 10.1186/s12889-016-3956-2 (PMC5228109; doi:10.1186/s12889-016-3956-2)
Supplement: Additional file 4: — Narrative description of key characteristics of included studies. (DOCX 118 kb) [file 12889_2016_3956_MOESM4_ESM.docx]

**Additional file 4. Narrative description of key characteristics of included studies.**

***Description of included randomised controlled trials***

Twelve of 26 included studies were classified as RCTs (Aaron, Mela, & Evans, 1994; Crockett, Jebb, Hankins, & Marteau, 2014; Ebneter, Latner, & Nigg, 2013; French et al., 2001; Kähkönen, Tuorila, & Lawless, 1997; Kähkönen, Hakanpää, & Tuorila, 1999a; Kruja, 2014; Norton, Fryer, & Parkinson, 2013; Stubenitsky, Aaron, Catt, & Mela, 1999; Wansink & Chandon, 2006 (S2); Wansink & Chandon, 2006 (S3); Wardle & Solomons, 1994 – see also Appendix B). All 12 RCTs investigated food labelling and their key characteristics and results are summarised in Supplemental Data Table 1.

Eleven of these RCTs were individually randomised – of which 7 had a parallel group (between-subjects) design (Crockett, Jebb, Hankins, & Marteau, 2014; Ebneter, Latner, & Nigg, 2013; Kähkönen, Tuorila, & Lawless, 1997; Kähkönen, Hakanpää, & Tuorila, 1999a; Stubenitsky, Aaron, Catt, & Mela, 1999; Wansink & Chandon, 2006 (S2); Wansink & Chandon, 2006 (S3)) and 4 had a crossover (within-subjects) design (Aaron, Mela, & Evans, 1994; Kruja, 2014; Norton, Fryer, & Parkinson, 2013; Wardle & Solomons, 1994) – and the other was a cluster RCT with a crossover design (French et al., 2001). Five RCTs were conducted in the United Kingdom (Aaron, Mela, & Evans, 1994; Crockett, Jebb, Hankins, & Marteau, 2014; Norton, Fryer, & Parkinson, 2013; Stubenitsky, Aaron, Catt, & Mela, 1999; Wardle & Solomons, 1994), 5 in the United States of America (Ebneter, Latner, & Nigg, 2013; French et al., 2001; Kruja, 2014; Wansink & Chandon, 2006 (S2); Wansink & Chandon, 2006 (S3)) and 2 in Finland (Kähkönen, Tuorila, & Lawless, 1997; Kähkönen, Hakanpää, & Tuorila, 1999a). Seven were conducted in controlled laboratory settings (e.g. ‘individual tasting booths’ (Kähkönen, Hakanpää, & Tuorila, 1999a), or ‘a purpose-built sensory evaluation laboratory, under dim red-light’ (Aaron, Mela, & Evans, 1994)) while 5 were conducted in real world environments (e.g. in school or worksite cafeterias (Kähkönen, Tuorila, & Lawless, 1997), cinema auditoria (Crockett, Jebb, Hankins, & Marteau, 2014; Wansink & Chandon, 2006 (S3)), or in the home (Stubenitsky, Aaron, Catt, & Mela, 1999) – setting was unclear in one RCT (Ebneter, Latner, & Nigg, 2013)).

All 12 included RCTs assessed the effects of exposure to a label that included a descriptor denoting the presence of a small or zero amount of fat in a labelled food product (a total of 15 eligible within-study comparisons). The specific label descriptor varied between studies (Supplemental Data Table 1), with 6 of 15 comparisons investigating labels incorporating ‘absolute descriptors’ (‘Low fat’ or ‘Low-Fat’), 6 investigating ‘relative descriptors’ (‘Reduced-fat’ or ‘Reduced-Fat’) and 2 investigating ‘absence descriptors’ (‘Fat-free’ or ‘Fat Free’) (precise wording was not clearly reported for one comparison). Comparators also varied between RCTs: in 8 of 15 comparisons the low fat label was compared with no equivalent labelling, whilst in the other 7 the low fat label was compared with a label that included a descriptor denoting the presence of a larger *absolute* amount of fat in the same product (‘Full-fat’, ‘Full Fat’ or ‘Regular’) (Supplemental Data Table 1). The range of labelled food products investigated among 12 included RCTs comprised: cheese spread sandwiches (Wardle & Solomons, 1994), chocolate bars (Kähkönen, Hakanpää, & Tuorila, 1999a; Norton, Fryer, & Parkinson, 2013; Stubenitsky, Aaron, Catt, & Mela, 1999), regular granola (Wansink & Chandon, 2006 (S2); Wansink & Chandon, 2006 (S3)), low-fat chocolate milk (Kruja, 2014), low-fat spread (Aaron, Mela, & Evans, 1994), low-fat snack products (various, not specified) (French et al., 2001); M&Ms (Ebneter, Latner, & Nigg, 2013; Wansink & Chandon, 2006 (S2)), salted or toffee popcorn (Crockett, Jebb, Hankins, & Marteau, 2014), frankfurter sausages (Kähkönen, Hakanpää, & Tuorila, 1999a), pork sausages (Stubenitsky, Aaron, Catt, & Mela, 1999), and low-fat strawberry yoghurt (Kähkönen, Tuorila, & Lawless, 1997). In 7 of 15 comparisons, the labels were placed on, or incorporated into, the product or its packaging, while in the other 8 the labels were placed adjacent to the product or its packaging. In 13 of 15 eligible comparisons, exposed participants tasted or consumed the food as part of the study procedure. In 4 of 15 eligible comparisons (4 eligible RCTs), participants were exposed to a concurrent intervention in addition to being exposed to the ‘low fat’ or equivalent label, the ‘higher fat’ or equivalent label, and/or no equivalent labelling condition(s). In 3 of these 4 comparisons (RCTs), the concurrent intervention (‘exposure to price information on the labels’ (French et al., 2001), ‘exposure to label information repeated at the top of the question form’ (Kähkönen, Tuorila, & Lawless, 1997) and ‘exposure to a leaflet containing information about heart disease delivered prior to exposure to labels’ (Wardle & Solomons, 1994) respectively) was provided to participants in both comparison groups (conditions). In the other of these 4 comparisons (RCTs), the concurrent intervention (‘exposure to green colour coding on the label’) was provided to participants in the intervention group only) (Crockett, Jebb, Hankins, & Marteau, 2014). In the latter case, it is not possible to disaggregate the independent study-level effects of exposure to the ‘low fat’ label from the effect of the green colour of the label (Crockett, Jebb, Hankins, & Marteau, 2014).

Two RCTs recruited female participants only (undergraduate students) (Ebneter, Latner, & Nigg, 2013; Kruja, 2014), while the other 10 recruited both female and male participants. The mean proportion of female participants among 10 of 12 RCTs reporting these data was 71% (range: 50-100%). Of note, 5 of the 12 RCTs exclusively recruited participants from staff and/or student bodies of higher education institutions (Ebneter, Latner, & Nigg, 2013; Kähkönen, Hakanpää, & Tuorila, 1999a; Kruja, 2014; Norton, Fryer, & Parkinson, 2013; Wardle & Solomons, 1994). The mean age within RCT participant samples ranged between 19.5 (Kruja, 2014) and 40.5 years (Aaron, Mela, & Evans, 1994). Few RCTs reported on the socio-economic or body weight status of their participants and few reported levels of dietary restraint (Herman & Polivy, 1980). With regards to primary outcomes, 2 of 12 included RCTs measured quantities of labelled food selected (with or without purchase) by exposed participants (Aaron, Mela, & Evans, 1994; French et al., 2001), while five measured quantities of food they consumed (Crockett, Jebb, Hankins, & Marteau, 2014; Ebneter, Latner, & Nigg, 2013; Kruja, 2014; Wansink & Chandon, 2006 (S3); Wardle & Solomons, 1994). With regards to secondary outcomes, none of the included RCTs measured intentions to select, purchase, or consume the product. Two of 12 included RCTs incorporated measures of beliefs associated with consumption (Stubenitsky, Aaron, Catt, & Mela, 1999; Wansink & Chandon, 2006 (S2)), 9 incorporated measures of product appeal (Aaron, Mela, & Evans, 1994; Crockett, Jebb, Hankins, & Marteau, 2014; Ebneter, Latner, & Nigg, 2013; Kähkönen, Tuorila, & Lawless, 1997; Kähkönen, Hakanpää, & Tuorila, 1999a; Kruja, 2014; Norton, Fryer, & Parkinson, 2013; Stubenitsky, Aaron, Catt, & Mela, 1999; Wardle & Solomons, 1994), and 4 incorporated measures of understanding of the label (Ebneter, Latner, & Nigg, 2013; Kruja, 2014; Wansink & Chandon, 2006 (S2); Wansink & Chandon, 2006 (S3)). Funding sources were reported for 5 of the 12 included RCTs (Supplemental Data Table 1) but did not raise any concerns about funding by agencies that may have commercial interests in their results.

***Description of included non-randomised studies***

Fourteen of 26 included studies were classified as non-randomised studies (NRSs) (quasi-experimental or non-experimental studies) (Bui, Burton, Howlett, & Kozup, 2008; Bergen & Yeh, 2006; Dubbert, Johnson, Schlundt, & Montague, 1984; Kähkönen, Tuorila, & Rita, 1996; Kähkönen & Tuorila, 1998; Kiesel & Villas-Boas, 2013; Liem, Toraman Aydin, & Zandstra, 2012; Westcombe & Wardle, 1997; Bansal-Travers, Hammond, Smith, & Cummings, 2011; Cohen, Yang, & Donaldson, 2014; Hammond, Dockrell, Arnott, Lee, & McNeill, 2009; Hammond & Parkinson, 2009; Siahpush et al., 2011; Yong et al., 2011). The key characteristics and results of these 14 non-randomised studies are summarised in Supplemental Data Table 2. Only one of these 14 NRSs investigated alcohol product labelling (Bui, Burton, Howlett, & Kozup, 2008). This study was a non-randomised controlled trial with a crossover (within-subjects) design, conducted in a laboratory setting, which included assessment of the effects of exposure to a 12oz bottle of beer with labelling that included the descriptor ‘Light beer’ on measures of participants’ intention to consume the product and understanding of the label. Participants in this study were 230 female and male upper-level university students (mean age: 25 years) attending two universities, one in the Eastern and one in the Southern United States. The comparator – whilst not clearly described by the study authors – consisted of exposure to a 12oz bottle of beer with labelling that either: (a) incorporated an alternative descriptor (‘Regular beer’) to denote a higher strength version of the same product; or (b) carried no equivalent descriptor but clearly denoted a higher strength version of the same product (Bui, Burton, Howlett, & Kozup, 2008).

Of the remaining 13 non-randomised studies, 7 investigated food product labels (9 eligible comparisons) (Bergen & Yeh, 2006; Dubbert, Johnson, Schlundt, & Montague, 1984; Kähkönen, Tuorila, & Rita, 1996; Kähkönen & Tuorila, 1998; Kiesel & Villas-Boas, 2013; Liem, Toraman Aydin, & Zandstra, 2012; Westcombe & Wardle, 1997) and 6 investigated tobacco product labels (8 eligible comparisons) (Bansal-Travers, Hammond, Smith, & Cummings, 2011; Cohen, Yang, & Donaldson, 2014; Hammond, Dockrell, Arnott, Lee, & McNeill, 2009; Hammond & Parkinson, 2009; Siahpush et al., 2011; Yong et al., 2011).

Among the 7 included non-randomised studies of food product labels, 3 were conducted in the USA (Bergen & Yeh, 2006; Dubbert, Johnson, Schlundt, & Montague, 1984; Kiesel & Villas-Boas, 2013), 2 in Finland (Kähkönen, Tuorila, & Rita, 1996; Kähkönen & Tuorila, 1998), 1 in the United Kingdom (Westcombe & Wardle, 1997) and 1 in The Netherlands (Liem, Toraman Aydin, & Zandstra, 2012). Two of these 7 studies were conducted in controlled laboratory settings (Liem, Toraman Aydin, & Zandstra, 2012; Westcombe & Wardle, 1997), while 4 were conducted in real world ‘micro-environments’ (e.g. ‘the main campus building in a large, urban college’ (Bergen & Yeh, 2006), or ‘supermarkets’ (Kiesel & Villas-Boas, 2013); setting was unclear in one study (Kähkönen, Tuorila, & Rita, 1996)). Two were classified as quasi-experimental studies (a controlled before-and-after study (Bergen & Yeh, 2006) and a difference-in-difference study (Kiesel & Villas-Boas, 2013)), 1 was classified as a non-randomised controlled trial with a crossover design (Westcombe & Wardle, 1997), 2 were classified as a before-and-after comparison (uncontrolled) (Kähkönen, Tuorila, & Rita, 1996; Liem, Toraman Aydin, & Zandstra, 2012), 1 was classified as a repeated cross-sectional study (Dubbert, Johnson, Schlundt, & Montague, 1984) and 1 was classified as a cross-sectional study (Kähkönen & Tuorila, 1998) (with respect to the eligible comparison(s) in each case). Three of these 7 studies (Kähkönen & Tuorila, 1998; Kiesel & Villas-Boas, 2013; Westcombe & Wardle, 1997) assessed the effects of exposure to a label that included a descriptor denoting the presence of a small or zero amount of fat in the labelled product (a total of 4 eligible comparisons), while others assessed label descriptors denoting the presence of small or zero amounts of: energy (2 studies, 2 eligible comparisons) (Dubbert, Johnson, Schlundt, & Montague, 1984; Kiesel & Villas-Boas, 2013); energy and carbohydrates (1 study, 1 eligible comparison) (Bergen & Yeh, 2006); fat and salt (1 study, 1 eligible comparison) (Kähkönen, Tuorila, & Rita, 1996); or salt (1 study, 1 eligible comparison) (Liem, Toraman Aydin, & Zandstra, 2012). The specific label descriptor varied among these 7 non-randomised studies of food product labels, with 4 of 9 comparisons investigating labels incorporating ‘absolute descriptors’ (‘Low-Fat’, ‘Low-fat, low salt’, ‘Light’ or ‘Low calorie’), 4 investigating ‘relative descriptors’ (‘Lower Fat’, ‘Lower calorie’ or ‘Reduced salt’) and 1 investigating ‘absence descriptors’ (‘0 Calorie, 0 Carbs’) (Supplemental Data Table 2). Comparators also varied: in 6 of 9 comparisons the ‘low’ label was compared with no equivalent labelling, while in 2 comparisons the ‘low’ label was compared with a label that included a descriptor denoting the presence of a larger *absolute* amount of the substance in the same product (‘Normal fat’ or ‘Regular’). The labelled food products investigated among these 7 non-randomised studies were: chicken 'Cup a Soup' (Liem, Toraman Aydin, & Zandstra, 2012), a low-fat, low salt spread (Kähkönen, Tuorila, & Rita, 1996); microwave popcorn (Kiesel & Villas-Boas, 2013); 12 non-alcoholic beverage product lines (including water, diet beverages, and sugar-sweetened soft drinks) (Bergen & Yeh, 2006); Bologna sausages (Kähkönen & Tuorila, 1998); and vegetable dishes, salads and healthier entrées (Dubbert, Johnson, Schlundt, & Montague, 1984); or cheese and yoghurt (Westcombe & Wardle, 1997). In 5 of 9 eligible comparisons, the labels were on placed on, or incorporated into, the product or its packaging, while in the other 8 comparisons the labels were placed adjacent to the product or its packaging (Supplemental Data Table 2). In 3 of 9 comparisons, exposed participants tasted or consumed the foods as part of the study procedure (Supplemental Data Table 2). In 1 of 9 comparisons, participants in the to the ‘low fat’ or equivalent label were exposed to an additional, concurrent intervention, namely ‘large posters placed at the entrance to the serving line stating “FOR YOUR INFORMATION, WE HAVE LOWERED SOME LOWER CALORIE ITEMS…Watch for these signs.” with a specimen label used to identify food items at point of display in the cafeteria line attached’ (Dubbert, Johnson, Schlundt, & Montague, 1984). In this case, it was not possible to disaggregate the independent study-level effects of exposure to the ‘low fat’ label from the effects of exposure to the large posters (Dubbert, Johnson, Schlundt, & Montague, 1984).

Six of 7 non-randomised food studies recruited both female and male adult participants; the other recruited male participants only (Kähkönen & Tuorila, 1998). The mean age within these studies’ participant samples ranged between 22.0 (Kähkönen & Tuorila, 1998) and 44.2 years (Liem, Toraman Aydin, & Zandstra, 2012). Two of these 7 studies recruited participants exclusively from staff and/or student bodies of higher education institutions (Bergen & Yeh, 2006; Kähkönen, Tuorila, & Rita, 1996). None reported on participants’ socio-economic status, body weight status or levels of dietary restraint. With regards to primary outcomes, 3 of these 7 studies assessed quantities of the labelled foods selected by exposed participants (Bergen & Yeh, 2006; Kiesel & Villas-Boas, 2013; Dubbert, Johnson, Schlundt, & Montague, 1984), but none investigated consumption. With regards to secondary outcomes, 1 of these 7 studies assessed participants’ intention to select or purchase the product (Westcombe & Wardle, 1997), 1 assessed their intention to consume the product (Liem, Toraman Aydin, & Zandstra, 2012), 2 assessed beliefs associated with consumption (Liem, Toraman Aydin, & Zandstra, 2012; Westcombe & Wardle, 1997), 4 assessed product appeal (Kähkönen, Tuorila, & Rita, 1996; Kähkönen & Tuorila, 1998; Liem, Toraman Aydin, & Zandstra, 2012; Westcombe & Wardle, 1997) and none assessed understanding of the label.

Among the 6 included non-randomised studies of tobacco product labels (Bansal-Travers, Hammond, Smith, & Cummings, 2011; Cohen, Yang, & Donaldson, 2014; Hammond, Dockrell, Arnott, Lee, & McNeill, 2009; Hammond & Parkinson, 2009; Siahpush et al., 2011; Yong et al., 2011), 2 studies involved only Canadian participants (Cohen, Yang, & Donaldson, 2014; Hammond & Parkinson, 2009), 1 involved participants from Australia, Canada and the UK (Yong et al., 2011), and individual studies involved participants from Thailand (Siahpush et al., 2011), the USA (Bansal-Travers, Hammond, Smith, & Cummings, 2011) and the UK (Hammond, Dockrell, Arnott, Lee, & McNeill, 2009). Three of these 6 studies were case series studies that investigated adult smokers’ cigarette consumption and/or their beliefs associated with consumption before and after implementation of national bans the use of descriptors such as ‘Light’ and ‘Mild’ on cigarette packs offered for sale (Cohen, Yang, & Donaldson, 2014; Siahpush et al., 2011; Yong et al., 2011). It is important to highlight that these bans were implemented in response to smokers’ incorrect beliefs that cigarettes in packs labelled with such descriptors are ‘less harmful’ than regular cigarettes, which has been proposed to discourage and/or delay cessation. However, in the context of this systematic review, the eligible comparison within each of these 3 studies is of ‘exposure to cigarette packages for sale including packages that contain ‘Light’, ‘Mild’ or equivalent descriptors’ (i.e. *pre-ban* = intervention) with ‘exposure to cigarette packages for sale *not* including packages that contain ‘Light’, ‘Mild’ or equivalent descriptors’ (i.e. *post-ban* = comparator). The other three non-randomised studies of tobacco product labels (a mall-intercept study (Hammond & Parkinson, 2009), an online study (Hammond, Dockrell, Arnott, Lee, & McNeill, 2009) and a face-to-face study in which the setting was unclear (Bansal-Travers, Hammond, Smith, & Cummings, 2011)) were classified as cross-sectional studies in which participants were asked to rate a series of (pairs of) cigarette packs with variant characteristics (including, but not limited to, variant label descriptors) in response to questions about beliefs associated with consumption, product appeal and understanding of the label. Within each of these 3 studies, only one or two pairwise comparisons among a much larger set of comparisons between (pairs of) packs were eligible for consideration in this systematic review. The brand of cigarette packs used in each of these three cross-sectional studies was, respectively, *Kent*^TM^ (Hammond & Parkinson, 2009), *Mayfair*^TM^ (Bansal-Travers, Hammond, Smith, & Cummings, 2011) or *Richmond*^TM^ (Hammond, Dockrell, Arnott, Lee, & McNeill, 2009). Two of these three studies recruited mixed samples of smokers and non-smokers (roughly evenly split samples) (Bansal-Travers, Hammond, Smith, & Cummings, 2011; Hammond & Parkinson, 2009) whilst the other exclusively recruited smokers (Hammond, Dockrell, Arnott, Lee, & McNeill, 2009). All 6 non-randomised studies of tobacco product labels recruited female and male adult participants (Supplemental Data Table 2). The mean age within these 6 studies’ participant samples ranged between 28.6 (Bansal-Travers, Hammond, Smith, & Cummings, 2011) and 44.0 years (Siahpush et al., 2011). Five of these 6 studies reported on their participants’ socio-economic status (Supplemental Data Table 2). The proportion of study participants classified as belonging to low socio-economic status groups ranged between 46.5% (Hammond & Parkinson, 2009) and 57% (Hammond, Dockrell, Arnott, Lee, & McNeill, 2009), indicating that these samples broadly reflected current social patterning of tobacco use. Overall, funding sources were reported for 10 of the 14 included non-randomised studies but did not raise any concerns about funding by agencies that may have commercial interests in their results.
