# Supplementary material for: What do we know about the effects of exposure to ‘Low alcohol’ and equivalent product labelling on the amounts of alcohol, food and tobacco people select and consume? A systematic review
Source: BMC Public Health. 2017 Jan 12;17:29. doi: 10.1186/s12889-016-3956-2 (PMC5228109; doi:10.1186/s12889-016-3956-2)
Supplement: Additional file 5: Table S2. — Characteristics and results of included non-randomised studies. (DOCX 93 kb) [file 12889_2016_3956_MOESM5_ESM.docx]

**Additional file 5: Table S2 Characteristics and results of included non-randomised studies (N=14).**

| *Study of alcohol product labels* | | | | | |  | |  | |  | |  | |
| --- | --- | --- | --- | --- | --- | --- | --- | --- | --- | --- | --- | --- | --- |
| *Study* | *Funding source* | *Design* | *Country, setting* | *Participants that completed study* | | | *Intervention(s)* | | *Comparator(s)* | | *Outcomes assessed* | | *Result(s)* |
| Bui, Burton, Howlett, & Kozup, 2008 [22]. | Funding source (if any) is not reported. | NRS-NE  NRCT-C | USA, Laboratory. | 230 upper-level university students; age (M=25.0yrs, SD=NR);  NR% female. | | | Exposure to a 12oz bottle of beer with labelling that includes the descriptor ‘Light beer’. | | Exposure to a 12oz bottle of beer with equivalent labelling that either: (a) incorporates an alternative descriptor (‘Regular beer’) to denote a higher strength version of the same product; or (b) carries no equivalent descriptor but is clearly denoted as a higher strength version of the same product. | | *Intention to consume: -*  ‘‘Given the information shown on the front and the back of the mock bottle, would the available information increase or decrease the amount you would drink, that is, your consumption level?’’ (9-point scale ‘’would decrease consumption level’ to ‘would increase consumption level’).  *Understanding of label:-*  Perceived calorie content of product - ‘Please rate the nutrient levels.’ (9-point scale from 1 ‘indicates that you think the level of the nutrient is very low’ to 9 ‘indicates that the level is very high’).  Perceived carbohydrate content of product - ‘Please rate the nutrient levels.’ (9-point scale from 1 ‘indicates that you think the level of the nutrient is very low’ to 9 ‘indicates that the level is very high’).  Perceived fat content of product - ‘Please rate the nutrient levels.’ (9-point scale from 1 ‘indicates that you think the level of the nutrient is very low’ to 9 ‘indicates that the level is very high’). | | Unclear  Negative association  Negative association  Negative association |
| *Studies of food product labels* | | | | | | |  | |  | |  | |  |
| *Study* | *Funding source* | *Design* | *Country, setting* | | *Participants that completed study* | | *Intervention(s)* | | *Comparator(s)* | | *Outcomes assessed* | | *Result(s)* |
| Bergen & Yeh, 2006 [21]. | Dorothy Epstein Nutrition Fellows award. | NRS-QE  CBA | USA, Real-world micro-environment (8 vending machines in a main campus building of a large urban college). | | NR college students, professors or staff members who could potentially purchase beverages from the vending machines. | | Exposure to12 non-alcoholic beverage product lines (including water, diet beverages, and sugar-sweetened beverages) in vending machines that had bright-coloured ‘0 Calorie, 0 Carbs’ labels placed on the selection panels of water and non-energy containing products.¶ | | Exposure to12 non-alcoholic beverage product lines (including water, diet beverages, and sugar-sweetened beverages) in vending machines with no equivalent labelling of water and non-energy containing products. | | *Selection:-*  Average (mean) quantity of water products purchased per week (number of bottles).  Average (mean) quantity of diet beverage products purchased per week (number of bottles).    Average (mean) quantity of sugar-sweetened beverage products purchased per week (number of bottles).  Average (mean) revenue from water products per week (USD).  Average (mean) revenue from diet beverage products per week (USD).  Average (mean) revenue from sugar-sweetened beverage products per week (USD). | | ND  ND  ND  ND  ND  ND |
| Dubbert, Johnson, Schlundt, & Montague, 1984 [25]. | Funding source (if any) is not reported. | NRS-NE  RXS | USA, Real-world micro-environment (cafeteria located near a large medical centre and between a business area and middle-class residential neighbourhood). | | 413 cafeteria customers;  age NR;  55% female. | | Exposure to a cafeteria line with labels containing the descriptor ‘lower calorie’ placed adjacent to an incrementally increasing number of food items (3 vegetable dishes; 3 vegetable dishes and 3 salads; 3 vegetable dishes, 3 salads and 3 entrées) over 9 weeks¶.  [+ *Exposure to large posters placed at the entrance to the serving line stating "FOR YOUR INFORMATION, WE HAVE LOWERED SOME LOWER CALORIE ITEMS…Watch for these signs.” with a specimen label used to identify food items at point of display in the cafeteria line attached*]. | | Exposure to a cafeteria line offering the same products with no equivalent labelling over 6 weeks. | | *Consumption:-*  Probability of purchasing lower calorie vegetable dishes.  Probability of purchasing lower calorie salads.  Probability of purchasing lower calorie. | | Positive association  (3, 6 and 9)  Positive association  (6 and 9); ND (3)  ND (3, 6 and 9) |
| Kähkönen, Tuorila, & Rita, 1996 [30-31]. | Funding source (if any) is not reported. | NRS-NE  BA | Finland, Unclear (not described). | | 50 university employees or students; age  (M=38.0yrs, SD=NR);  60% female. | | Exposure to a low-fat, low salt spread labelled with the descriptor ‘Low-fat spread (40% fat), low salt’. | | Exposure to a low-fat, low salt spread labelled with no equivalent labelling. | | *Appeal:-*  Pleasantness (9-point scale ‘extremely unpleasant’ to ‘extremely pleasant’).  Intensity of saltiness (9-point scale ‘saltiness, too weak’ thru ‘appropriate saltiness’ to ‘saltiness, too strong’).  Melting rate in the mouth (9-point scale ‘melts slowly in the mouth’ to ‘melts quickly in the mouth'). | | Positive association  ND  Positive association |
| Kähkönen & Tuorila, 1998 [31, 33]. | Academy of Finland (Project 33251). | NRS-NE  XS | Finland, Real-world micro-environment (worksite cafeteria -mess hall in an army training centre). | | 115 men on compulsory military service§;  age  (M=22.0yrs, SD=NR);  0% female. | | Exposure to covered plate containing sausages labelled as ‘Light Bologna (10% fat)’. | | Exposure to covered plate containing sausages labelled as ‘Regular type of Bologna (20% fat)’ | | *Appeal:-*  Expected pleasantness (9-point scale ‘extremely unpleasant’ to ‘extremely pleasant’).  Expected juciness (9-point scale ‘not at all juicy’ to ‘extremely juicy).  Expected saltiness (9-point scale ‘not at all salty' to ‘extremely salty’).  Expected fatiness (9-point scale ‘not at all fatty’ to ‘extremely fatty’). | | Negative association  Negative association  Negative association  Negative association |
| Kiesel & Villas-Boas, 2013 [35]. | Giannini Foundation. | NRS-QE  DID | USA, Real-world micro-environment (32 supermarkets). | | NR supermarket customers; age NR; NR% female. | | Exposure to a microwave popcorn product line on a supermarket shelf with a label placed adjacent to the price tag on the shelf containing the descriptor ‘Low calorie’.  Exposure to a microwave popcorn product line on a supermarket shelf with a label placed adjacent to the price tag on the shelf containing the descriptor ‘Low fat’. | | Exposure to a microwave popcorn product line on a supermarket shelf with no equivalent label placed adjacent to the price tag on the shelf.  Exposure to a microwave popcorn product line on a supermarket shelf with no equivalent label placed adjacent to the price tag on the shelf. | | *Selection:-*  Average (mean) quantity of microwave popcorn sold over a 4-week period (number of bags).  *Selection:-*  Average (mean) quantity of microwave popcorn sold over a 4-week period (number of bags). | | Positive association  Negative association |
| Liem, Toraman Aydin, & Zandstra, 2012 [37]. | Funding source (if any) is not reported. | NRS-NE  BA | Netherlands, Laboratory (‘Controlled laboratory-like environment’). | | 46 adults; age (M=44.2yrs, SD=13.8);  61% female. | | Exposure to a box containing one pouch of chicken 'Cup a Soup' and labelled with the text ‘Now with reduced salt’. | | Exposure to a box containing one pouch of chicken 'Cup a Soup' and no equivalent labelling. | | *Intention to consume:-*  Expected desire to consume - ‘‘How much do you want to have a taste of this soup?’’ (7-point scale ‘do not want at all’ to ‘want extremely’).  Actual perceived desire to consume - ‘‘How much do you want to have another taste of this soup?’' (7-point scale ‘do not want at all’ to ‘want extremely’).  *Belief associated with consumption:-*  ‘‘I think this product will be healthy’’ (7-point scale ‘not agree at all’ to ‘totally agree’).  *Appeal:-*  Expected salt intensity -‘‘How salty do you think this soup will taste?’’ (7-point scale ‘not salty at all’ to ‘extremely salty’).  Expected liking - ‘‘How much do you think you will like this soup?’’ (7-point scale ‘won’t like at all’ to ‘will like extremely’).  Actual perceived salt intensity - ‘‘How salty do you think this soup tastes?’’ (7-point scale ‘not salty at all’ to ‘extremely salty’).  Actual perceived liking - ‘‘How much do you like the taste of the soup?’’ (7-point scale ‘do not like at all’ to ‘like extremely’).  Credibility - ‘‘I think this package is credible’’ (7-point scale ‘not agree at all’ to ‘totally agree’).  Attitude towards package - ‘‘I hold a positive attitude towards this package’’ (7-point ‘not agree at all’ to ‘totally agree’). | | ND  ND  Negative association  Negative association  ND  ND  ND  ND  Unclear |
| Westcombe & Wardle, 1997 [44]. | Studentship grant from the Biotechnology and Biological Sciences Research Council, UK. | NRS-NE  NRCT-C | UK, Laboratory. | | 36 normal weight adults; age (M=28.0yrs, SD=8.1);  50% female. | | Exposure to foods labelled as ‘Lower fat’.  Exposure to foods labelled as ‘Lower fat’. | | Exposure to foods labelled as ‘Normal fat’.  Exposure to foods labelled as ‘Higher fat’. | | *Intention to select or purchase:-*  Likelihood of buying (7-point scale ‘not at all likely to buy’ to ‘extremely likely to buy’).  *Belief associated with consumption:-*  Healthiness (7-point scale ‘not at all healthy’ to ‘extremely healthy’).  Amount of fat (7-point scale ‘not at all fatty’ to ‘extremely fatty’).  *Appeal:-*  Pleasantness (9-point scale ‘extremely unpleasant’ to extremely pleasant’).  *Intention to select or purchase:-*  Likelihood of buying (7-point scale ‘not at all likely to buy’ to ‘extremely likely to buy’).  *Belief associated with consumption:-*  Healthiness (7-point scale ‘not at all healthy’ to ‘extremely healthy’).  Amount of fat (7-point scale ‘not at all fatty’ to ‘extremely fatty’).  *Appeal:-*  Pleasantness (9-point scale ‘extremely unpleasant’ to extremely pleasant’). | | (+)  (+)  –  (–)  (+)  (+)  (–)  (–) |
| *Studies of tobacco product labels* | | | | | | |  | |  | |  | |  |
| *Study* | *Funding source* | *Design* | *Country, setting* | | *Participants that completed study* | | *Intervention(s)* | | *Comparator(s)* | | *Outcomes assessed* | | *Result(s)* |
| Bansal-Travers, Hammond, Smith, & Cummings, 2011 [20]. | Developmental Research Grant, Transdisciplinary Tobacco Use Research Center, Roswell Park Cancer Institute, NCI grant P50 CA 111236; P01 CA138389 (Roswell Park Cancer Institute, Buffalo NY), US National Cancer Institute. | NRS-NE  XS | USA, Unclear (not described). | | 397 adults aged ≥18 years; age (M=28.6yrs, SD=11.6);  49% female; 50% low SES (education: completed high school or less); 50% smokers. | | Exposure to a Mayfair branded cigarette pack including the label descriptor ‘Light’. | | Exposure to a Mayfair branded cigarette pack including the label descriptor ‘Full flavor’. | | *Intention to select or purchase product:-*  ‘Which product would you purchase?’ (‘Light’ or ‘Full flavor’).  *Belief associated with consumption:-*  ‘Which product would you buy if you were trying to reduce the risks to your health?’ (‘Light’ or ‘Full flavor’).  *Appeal:-*  ‘Which product has the smoothest taste?’ (‘Light’ or ‘Full flavor’).  *Understanding of Label:-* ‘  Which product contains the most tar?’ (‘Light’ or ‘Full flavor’). | | 62% ‘Light’, 32% ‘Full flavor’.  94% ‘Light’, 4% ‘Full flavor’.  77% ‘Light’, 22% ‘Full flavor’.  92% ‘Light’, 4% ‘Full flavor’. |
| Cohen, Yang, & Donaldson, 2014 [23]. | National Cancer Institute grant (T32 CA009314) and the Center for a Livable Future Lerner Fellowship. | NRS-NE  CS | Canada, Real-world micro-environments (free-living conditions). | | 632 adult smokers; age (M= 43.0yrs, SD=14.9);  55% female; 48% low SES (education: high school degree or less); 100% smokers. | | Exposure to cigarette packages for sale that included packages containing the descriptors ‘Light’, ‘Mild’, ‘Ultra-light’ and ‘Ultra-mild’ (i.e. pre-nationwide ban). | | Exposure to cigarette packages for sale that did not include packages containing the descriptors ‘Light’, ‘Mild’, ‘Ultra-light’ and ‘Ultra-mild’ (i.e. post- nationwide ban). | | *Consumption:-*  Smoking maintenance rate among all smokers (%).  Quit rate in last six months among all smokers (%).  Consumption of light brands among all smokers (%).  Consumption of regular brands among all smokers (%).  Consumption of light replacement brands among all smokers (%). | | Unclear.  Unclear.  Unclear.  Unclear.  Unclear. |
| Hammond, Dockrell, Arnott, Lee, & McNeill, 2009 [28]. | British Heart Foundation and Canter Research UK. | NRS-NE  XS | UK, Laboratory (online study). | | 516 adult smokers§;  age (M= 38.5yrs, SD=13.6); 51% female; 47% low SES (occupation: social grade C2DE); 100% smokers. | | Exposure to a Richmond branded cigarette pack including the label descriptor ‘Light’. | | Exposure to a Richmond branded cigarette pack with a blank space on the label where 'Smooth' would usually appear (i.e. no equivalent labelling). | | *Belief associated with consumption:-*  ‘If you were to choose between them, which one would you buy if you were trying to reduce the risk to your health?’ (‘Light’, pack with no equivalent labelling, or indifferent).  ‘Which brand do you think would make it easier to quit smoking?’ (‘Light’, pack with no equivalent labelling, or neither).  *Appeal:-*  ‘Which brand do you think would have the smoothest taste if you were to smoke it?’ (‘Light’, pack with no equivalent labelling, or indifferent).  ‘Of these two brands, which is the most attractive?’ (Light’, pack with no equivalent labelling, or indifferent).  *Understanding of label:-*  ‘Which brand do you think would have less tar if you were to smoke it?’ (‘Light’, pack with no equivalent labelling, or indifferent). | | 57% ‘Light’, 3% pack with no equivalent labelling, 40% indifferent.  33% ‘Light’, 6% pack with no equivalent labelling, 61% indifferent.  52% ‘Light’, 9% pack with no equivalent labelling, 39% indifferent.  18% ‘Light’, 7% pack with no equivalent labelling, 75% indifferent.  69% ‘Light’, 3% pack with no equivalent labelling, 28% indifferent. |
| Hammond & Parkinson, 2009 [29]. | Centre for Behavioural Research and Program Evaluation, National Cancer Institute of Canada/Canadian Cancer Society. | NRS-NE  XS | Canada, Laboratory (mall intercept study). | | 603 adults aged ≥18 years;  age (M= 39.0yrs, SD=15.0); 44% female; 57% low SES (education: completed high school or less); 52% smokers. | | Exposure to a Kent branded cigarette pack including the label descriptor ‘Light’.  Exposure to a Kent branded cigarette pack including the label descriptor ‘Mild’.  Exposure to a Kent branded cigarette pack including the label descriptor ‘Ultra light’. | | Exposure to a Kent branded cigarette pack including the label descriptor ‘Full flavor’.  Exposure to a Kent branded cigarette pack including the label descriptor ‘Regular’.  Exposure to a Kent branded cigarette pack including the label descriptor ‘Light’. | | *Belief associated with consumption:-*  ‘If you were to choose between these two brands, which one would you buy if you were trying to reduce the risks to your health?’ (‘Light’, ‘Full flavor’ or indifferent).  *Appeal:-*  ‘Which brand would you expect to have the smoothest taste?’ (‘Light’, ‘Full flavor’ or indifferent).  *Understanding of label:-*  ‘Which brand would you expect to deliver less tar if you were to smoke it?’.(‘Light’, ‘Full flavor’ or indifferent).  *Belief associated with consumption:-*  ‘If you were to choose between these two brands, which one would you buy if you were trying to reduce the risks to your health?’ (‘Mild’, ‘Regular’ or indifferent).  *Appeal:-*  ‘Which brand would you expect to have the smoothest taste?’ (‘Mild’, ‘Regular’ or indifferent).  *Understanding of label:-*  ‘Which brand would you expect to deliver less tar if you were to smoke it?’.(‘Mild’, ‘Regular’ or indifferent).  *Belief associated with consumption:-*  ‘If you were to choose between these two brands, which one would you buy if you were trying to reduce the risks to your health?’ (‘Ultra light’, ‘Light’ or indifferent).  *Appeal:-*  ‘Which brand would you expect to have the smoothest taste?’ (‘Ultra light’, ‘Light’ or indifferent).  *Understanding of label:-*  ‘Which brand would you expect to deliver less tar if you were to smoke it?’.(‘Ultra light’, ‘Light’ or indifferent). | | 87% ‘Light’, 7% ‘Full flavor’, 6% indifferent.  74% ‘Light’, 23% ‘Full flavor’, 3% indifferent.  91% ‘Light’, 5% ‘Full flavor’, 4% indifferent.  86% ‘Mild’, 9% ‘Regular’, 5% indifferent.  77% ‘Mild’, 20% ‘Regular’, 3% indifferent.  91% ‘Mild’, 4% ‘Regular’, 3% indifferent.  76% ‘Ultra light’, 17% ‘Light’, 7% indifferent.  62% ‘Ultra light’, 35% ‘Light’, 3% indifferent.  89% ‘Ultra light’, 7% ‘Light’, 4% indifferent. |
| Siahpush et al., 2011 [39]. | Roswell Park Transdisciplinary Tobacco Use Research Center (P50 CA111236), National Cancer Institute of the United States (P01 CA138389), Canadian Institutes of Health Research (79551), Ontario Institute for Cancer Research, Thai Health Promotion Foundation, and the Malaysian Ministry of Health. | NRS-NE  CS | Thailand, Real-world micro-environments (free-living conditions). | | 2,352 adult smokers;  age (M= 44.0yrs, SD=NR); 7% female; 100% smokers. | | Exposure to cigarette packets for sale with labelling that contains the descriptor ‘Light’ (i.e. pre-nationwide ban). | | Exposure to cigarette packets for sale with no equivalent labelling (i.e. post-nationwide ban). | | *Consumption:-*  Proportion of smokers smoking 'light' cigarette brands (%).  Proportion of smokers smoking 'regular' cigarette brands (%).  Proportion of smokers smoking 'roll your own' cigarettes (%).  *Beliefs associated with product consumption:-*  Average (mean) level of agreement with the statement ‘Light cigarettes are less harmful than regular cigarettes’ (5-point scale ‘strongly disagree’ to ‘strongly agree’).  Proportion of smokers agreeing with the statement ‘Light cigarettes are less harmful than regular cigarettes’ (%).  Average (mean) level of agreement with the statement ‘Light cigarettes are smoother on your throat and chest than regular cigarettes’ (5-point scale ‘strongly disagree’ to ‘strongly agree’).  Proportion of smokers agreeing with the statement ‘Light cigarettes are smoother on your throat and chest than regular cigarettes’ (%). | | (–)  (–)  Unclear  (+)  (+)  (+)  (+) |
| Yong et al., 2011 [45]. | Roswell Park Transdisciplinary Tobacco Use Research Center (R01 CA100362 and P50 CA111236), Roswell Park Cancer Institute, (P01 CA138389), all funded by the National Cancer Institute of the United States, Robert Wood Johnson Foundation (045734), Canadian Institutes of Health Research (57897, 79551), National Health and Medical Research Council of Australia (265903, 450110), Cancer Research UK (C312/ A3726), Canadian Tobacco Control Research Initiative (014578) and Centre for Behavioural Research and Program Evaluation, National Cancer Institute of Canada/ Canadian Cancer Society. | NRS-NE  CS | Australia, Canada & UK, Real-world micro-environments (free-living conditions). | | 21,613 adult smokers§; aged ≥ 18 years (age NR); 55% female; 54% low SES (NR); 52% smokers. | | Exposure to cigarette packages for sale that include packages containing descriptors such as ‘Light’, ‘Mild’, and ‘Low in tar’ (i.e. pre-nationwide ban). | | Exposure to cigarette packages for sale with no equivalent labelling (i.e. post-nationwide ban). | | *Belief associated with consumption:-*  Average (mean) score on ‘light belief scale’ (computed as the mean of scaled scores from two questions eliciting levels of endorsement of incorrect beliefs about cigarettes promoted with terms such as light, mild or low in tar).  Average (mean) level of endorsement of belief that light taste means low tar (5-point scale ‘strongly agree’ to ‘strongly disagree’).  Average (mean) level of endorsement of belief that harsh smoke is more dangerous (5-point scale ‘strongly agree’ to ‘strongly disagree’).  Average (mean) level of endorsement of belief that tar numbers on packs are related to tar taken in (5-point scale ‘strongly agree’ to ‘strongly disagree’). | | (+) 1^st^ follow-up  ND 2^nd^ follow-up  (+) 1^st^ follow-up  ND 2^nd^ follow-up  (+) 1^st^ follow-up  ND 2^nd^ follow-up  (+) 1^st^ follow-up  ND 2^nd^ follow-up |

NRS-QE: Non-randomised study with a quasi-experimental design.

NRS-NE: Non-randomised study with a quasi-experimental design.

BA: Before-and-after comparison (uncontrolled).

CBA: Controlled before-and-after comparison.

CS: Case series study (uncontrolled longitudinal study).

DID: Difference in differences study.

N-RCT-C: Non-randomised controlled trial with a crossover (within-subjects) design.

RXS: Repeated cross-sectional study.

XS: Cross-sectional study.

NR: Not reported.
§ Data are not specific to eligible within-study comparison(s).

¶ Label placed adjacent to the product or its packaging rather than placed on, or incorporated into, the product or its packaging.

∱ Exposed participants tasted or consumed product as part of the study procedure.

[+ *Exposure to…*]: Description of concurrent intervention.

Positive association: Positive statistical association between exposure to ‘low’ label and outcome (i.e. exposure to low label associated with higher outcome values).

Negative association: Negative statistical association between exposure to ‘low’ label and outcome (i.e. exposure to low label associated with lower outcome values).

(+): Outcome values higher in the ‘low’ label condition.

(–): Outcome values lower in the ‘low’ label condition.

ND: No statistical association between exposure to ‘low’ label and outcome or no difference between comparison groups (conditions).
