# Supplementary material for: What do we know about the effects of exposure to ‘Low alcohol’ and equivalent product labelling on the amounts of alcohol, food and tobacco people select and consume? A systematic review
Source: BMC Public Health. 2017 Jan 12;17:29. doi: 10.1186/s12889-016-3956-2 (PMC5228109; doi:10.1186/s12889-016-3956-2)
Supplement: Additional file 6: — Study-level risk of bias table. (DOCX 187 kb) [file 12889_2016_3956_MOESM6_ESM.docx]

**Additional file 6 Study-level risk of bias tables**

Aaron, Mela, & Evans, 1994 [*Published data only*].

| **Bias** | **Authors' judgement** | **Support for judgement** |
| --- | --- | --- |
| Random sequence generation (selection bias) | Unclear | Comment: Method of sequence generation (order of exposures) is not described. Insufficient information about the sequence generation process to permit judgement of ‘Low risk’ or ‘High risk’. |
| Allocation concealment (selection bias) | Unclear | Comment: Insufficient information concerning method of concealment to permit judgement of ‘Low risk’ or ‘High risk’. |
| Selection: Blinding of participants and personnel (performance bias) | Low | Comment: No blinding or incomplete blinding of study participants but this is not judged likely to introduce risk of performance bias in the current context. Very unlikely that key study personnel were blinded, but the review authors judge that the outcome is not likely to be influenced by lack of blinding of key study personnel. |
| Consumption: Blinding of participants and personnel (performance bias) | Not measured |  |
| Intention to select or purchase: Blinding of participants and personnel (performance bias) | Not measured |  |
| Intention to consume: Blinding of participants and personnel (performance bias) | Not measured |  |
| Belief associated with consumption: Blinding of participants and personnel (performance bias) | Not measured |  |
| Appeal: Blinding of participants and personnel (performance bias) | Low | Quote: "The current study was advertised as a market research study involving a taste-rating task for a new type of M&M’s...While participants were not told that their intake was going to be measured, some participants may have anticipated this which may have in turn influenced their eating behaviors.". Comment: Blinding of study participants was not attempted with respect to this outcome but this is not judged likely to introduce risk of performance bias in the current context. Very unlikely that key study personnel were blinded, but the review authors judge that the outcome is not likely to be influenced by lack of blinding of key study personnel. |
| Understanding of label: Blinding of participants and personnel (performance bias) | Not measured |  |
| Selection: Blinding of outcome assessment (detection bias) | Low | Comment: No blinding of outcome assessment, but the review authors judge that the outcome measurement is not likely to be influenced by lack of blinding. |
| Consumption: Blinding of outcome assessment (detection bias) | Not measured |  |
| Intention to select or purchase: Blinding of outcome assessment (detection bias) | Not measured |  |
| Intention to consume: Blinding of outcome assessment (detection bias) | Not measured |  |
| Belief associated with consumption: Blinding of outcome assessment (detection bias) | Not measured |  |
| Appeal: Blinding of outcome assessment (detection bias) | Low | Comment: No blinding of outcome assessment, but the review authors judge that the outcome measurement is not likely to be influenced by lack of blinding. |
| Understanding of label: Blinding of outcome assessment (detection bias) | Not measured |  |
| Selection: Incomplete outcome data (attrition bias) | Low | Comment: No missing outcome data for selection outcome. |
| Consumption: Incomplete outcome data (attrition bias) | Not measured |  |
| Intention to select or purchase: Incomplete outcome data (attrition bias) | Not measured |  |
| Intention to consume: Incomplete outcome data (attrition bias) | Not measured |  |
| Belief associated with consumption: Incomplete outcome data (attrition bias) | Not measured |  |
| Appeal: Incomplete outcome data (attrition bias) | Low | Comment: No missing outcome data for product appeal outcome. |
| Understanding of label: Incomplete outcome data (attrition bias) | Not measured |  |
| Selective reporting (reporting bias) | Unclear | Comment: No evidence of selective outcome reporting based on study report. Search for record(s) containing details of study protocol conducted in ClinicalTrials.gov and the WHO International Clinical Trials Registry Platform (ICTRP) identified no record. Insufficient information to permit judgement of ‘Low risk’ or ‘High risk’. |
| Other bias | Low | Comment: Study is an individually randomised controlled trial with a crossover design. |
| Selection: Summary risk of bias | Unclear | Comment: Unclear risk of selection bias. |
| Consumption: Summary risk of bias | Not measured |  |
| Intention to select or purchase: Summary risk of bias | Not measured |  |
| Intention to consume: Summary risk of bias | Not measured |  |
| Belief associated with consumption: Summary risk of bias | Not measured |  |
| Appeal: Summary risk of bias | Unclear | Comment: Unclear risk of selection bias. |
| Understanding of label: Summary risk of bias | Not measured |  |

Crockett, Jebb, Hankins, & Marteau, 2014 [*Published data only*].

| **Bias** | **Authors' judgement** | **Support for judgement** |
| --- | --- | --- |
| Random sequence generation (selection bias) | Low | Quote: "On entering the auditorium a predetermined random number sequence was used to allocate participants, by unique study number, to study arm." |
| Allocation concealment (selection bias) | Low | Quote: "On arrival at the cinema participants were given a card with their unique study number printed on it. Study numbers were allocated sequentially according to the order in which participants arrived at the cinema. Participants were then directed through two sets of doors to the auditorium where the experiment took place. On entering the auditorium a predetermined random number sequence was used to allocate participants, by unique study number, to study arm." Comment: Sequences were not protected by concealment of the allocation sequence from those involved in the enrolment and assignment of participants, but the review authors judge - based on details of the randomisation procedure described in the study report - that lack of concealment did not increase risk of bias in intervention allocation. |
| Selection: Blinding of participants and personnel (performance bias) | Not measured |  |
| Consumption: Blinding of participants and personnel (performance bias) | Low | Quote: "As consumption is a behaviour that changes with the awareness of observation, participants were told that the study was concerned with the impact of taste on emotion and no mention was made that popcorn consumption would be assessed. At the end of the study participants were debriefed as to the nature of the study and were given the option of withdrawing from the study. No participant chose to do so… While we made every effort to exclude repeat attenders, it is possible that some are included in the dataset. Having read the debriefing sheet on their first attendance, they would have been aware of the purpose of the study. Additionally, some participants might have heard about the study, and its purpose, from those attending previous sessions. Those who knew the purpose of the study would know that all the popcorn had the same nutritional content regardless of label and thus their consumption would be unaffected by the label. However, any such participants would have been randomised across the groups so their knowledge would be likely to reduce rather than increase the effects found." Comment: No blinding or incomplete blinding of study participants but this is not judged likely to introduce risk of performance bias in the current context. Very unlikely that key study personnel were blinded, but the review authors judge that the outcome is not likely to be influenced by lack of blinding of key study personnel. |
| Intention to select or purchase: Blinding of participants and personnel (performance bias) | Not measured |  |
| Intention to consume: Blinding of participants and personnel (performance bias) | Not measured |  |
| Belief associated with consumption: Blinding of participants and personnel (performance bias) | Not measured |  |
| Appeal: Blinding of participants and personnel (performance bias) | Low | Quote: "As consumption is a behaviour that changes with the awareness of observation, participants were told that the study was concerned with the impact of taste on emotion and no mention was made that popcorn consumption would be assessed. At the end of the study participants were debriefed as to the nature of the study and were given the option of withdrawing from the study. No participant chose to do so… While we made every effort to exclude repeat attenders, it is possible that some are included in the dataset. Having read the debriefing sheet on their first attendance, they would have been aware of the purpose of the study. Additionally, some participants might have heard about the study, and its purpose, from those attending previous sessions. Those who knew the purpose of the study would know that all the popcorn had the same nutritional content regardless of label and thus their consumption would be unaffected by the label. However, any such participants would have been randomised across the groups so their knowledge would be likely to reduce rather than increase the effects found." Comment: Blinding of study participants attempted and it is possible that this blinding was broken in some cases - however, the review authors judge that performance bias, if present, would in this case lead to underestimation of the effect. Very unlikely that key study personnel were blinded, but the review authors judge that the outcome is not likely to be influenced by lack of blinding of key study personnel. |
| Understanding of label: Blinding of participants and personnel (performance bias) | Not measured |  |
| Selection: Blinding of outcome assessment (detection bias) | Not measured |  |
| Consumption: Blinding of outcome assessment (detection bias) | Low | Comment: No blinding of outcome assessment, but the review authors judge that the outcome measurement is not likely to be influenced by lack of blinding. |
| Intention to select or purchase: Blinding of outcome assessment (detection bias) | Not measured |  |
| Intention to consume: Blinding of outcome assessment (detection bias) | Not measured |  |
| Belief associated with consumption: Blinding of outcome assessment (detection bias) | Not measured |  |
| Appeal: Blinding of outcome assessment (detection bias) | Low | Comment: No blinding of outcome assessment, but the review authors judge that the outcome measurement is not likely to be influenced by lack of blinding. |
| Understanding of label: Blinding of outcome assessment (detection bias) | Not measured |  |
| Selection: Incomplete outcome data (attrition bias) | Not measured |  |
| Consumption: Incomplete outcome data (attrition bias) | Low | Quote: “Three hundred and twenty-five participants were recruited of whom 38 were excluded due to multiple attendances (n = 14), not leaving their popcorn bags (n = 13) or for failure to consume any popcorn (n = 11).” Comment: Reasons for excluding participants’ data from the analysis are provided in full. The review authors judge that the first two reasons provided for these exclusions by the study authors are reasonable from the perspective of minimizing bias. Exclusion of data collected from participants with ‘zero consumption’ is judged unreasonable, since this is likely to bias estimation of the intervention effect if these participants were unequally distributed between comparison groups. However, due to the small number of exclusions for this (11 participants, 3% of recruited study participants), the review authors judge that plausible effect size among missing outcomes is unlikely to be large enough to have an important impact on the observed effect size. |
| Intention to select or purchase: Incomplete outcome data (attrition bias) | Not measured |  |
| Intention to consume: Incomplete outcome data (attrition bias) | Not measured |  |
| Belief associated with consumption: Incomplete outcome data (attrition bias) | Not measured |  |
| Appeal: Incomplete outcome data (attrition bias) | Low | Quote: “Three hundred and twenty-five participants were recruited of whom 38 were excluded due to multiple attendances (n = 14), not leaving their popcorn bags (n = 13) or for failure to consume any popcorn (n = 11).” Comment: Reasons for excluding participants’ data from the analysis are provided in full. The review authors judge that the first two reasons provided for these exclusions by the study authors are reasonable from the perspective of minimizing bias. Exclusion of data collected from participants with ‘zero consumption’ is judged unreasonable, since this is likely to bias estimation of the intervention effect if these participants were unequally distributed between comparison groups. However, due to the small number of exclusions for this (11 participants, 3% of recruited study participants), the review authors judge that plausible effect size among missing outcomes is unlikely to be large enough to have an important impact on the observed effect size. |
| Understanding of label: Incomplete outcome data (attrition bias) | Not measured |  |
| Selective reporting (reporting bias) | Unclear | Quote: "Putative secondary endpoints (not reported here). These were completed at the beginning, midpoint and end of the experimental session to maintain the appearance that the purpose of the study was to assess the impact of emotion on taste: 1. Taste of the snack: five items assessed the extent to which the participant rated the popcorn as good tasting, strong tasting and unpleasant tasting. 2. Emotional state: six items assessed the extent to which the participant feels happy, relaxed, cheerful, tense, sad and upset.". Comment: No evidence of selective outcome reporting based on study report. Whilst the study authors measured, but did not reports results in relation to, product appeal (taste of the snack) and emotional state, these outcomes were measured for the sole purpose of maintaining the attempted blinding of participants to the purpose, hypotheses and primary outcome of the study. Search for record(s) containing details of study protocol conducted in ClinicalTrials.gov and the WHO International Clinical Trials Registry Platform (ICTRP) identified no record. Insufficient information to permit judgement of ‘Low risk’ or ‘High risk’. |
| Other bias | Unclear | Comment: Baseline comparability between comparison groups is not reported. Insufficient information to permit judgement of ‘Low risk’ or ‘High risk’. |
| Selection: Summary risk of bias | Not measured |  |
| Consumption: Summary risk of bias | Unclear | Comment: Unclear risk of other bias (baseline comparability between groups). |
| Intention to select or purchase: Summary risk of bias | Not measured |  |
| Intention to consume: Summary risk of bias | Not measured |  |
| Belief associated with consumption: Summary risk of bias | Not measured |  |
| Appeal: Summary risk of bias | Unclear | Comment: Unclear risk of other bias (baseline comparability between groups). |
| Understanding of label: Summary risk of bias | Not measured |  |

Ebneter, Latner, & Nigg, 2013 [*Published data only*].

| **Bias** | **Authors' judgement** | **Support for judgement** |
| --- | --- | --- |
| Random sequence generation (selection bias) | Unclear | Comment: Method of sequence generation is not described. Insufficient information about the sequence generation process to permit judgement of ‘Low risk’ or ‘High risk’. |
| Allocation concealment (selection bias) | Unclear | Comment: Insufficient information concerning method of concealment to permit judgement of ‘Low risk’ or ‘High risk’. |
| Selection: Blinding of participants and personnel (performance bias) | Not measured |  |
| Consumption: Blinding of participants and personnel (performance bias) | Low | Quote: "The current study was advertised as a market research study involving a taste-rating task for a new type of M&M’s...While participants were not told that their intake was going to be measured, some participants may have anticipated this which may have in turn influenced their eating behaviors.". Comment: No blinding or incomplete blinding of study participants but this is not judged likely to introduce risk of performance bias in the current context. Very unlikely that key study personnel were blinded, but the review authors judge that the outcome is not likely to be influenced by lack of blinding of key study personnel. |
| Intention to select or purchase: Blinding of participants and personnel (performance bias) | Not measured |  |
| Intention to consume: Blinding of participants and personnel (performance bias) | Not measured |  |
| Belief associated with consumption: Blinding of participants and personnel (performance bias) | Not measured |  |
| Appeal: Blinding of participants and personnel (performance bias) | Low | Quote: "The current study was advertised as a market research study involving a taste-rating task for a new type of M&M’s...While participants were not told that their intake was going to be measured, some participants may have anticipated this which may have in turn influenced their eating behaviors.". Comment: Blinding of study participants was not attempted with respect to this outcome but this is not judged likely to introduce risk of performance bias in the current context. Participants were probed for awareness of the manipulation after completing the study and only those participants who were aware of the manipulation were included in the analysis. (Quote: "Results of the manipulation check revealed that 175 out of 224 participants accurately identified whether they were presented with low-fat- versus regular-labeled M&M’s and whether they had caloric information available or not. Therefore, only results for the 175 participants who accurately recalled both of their experimental conditions are presented."). Very unlikely that key study personnel were blinded, but the review authors judge that the outcome is not likely to be influenced by lack of blinding of key study personnel. |
| Understanding of label: Blinding of participants and personnel (performance bias) | Low | Quote: "The current study was advertised as a market research study involving a taste-rating task for a new type of M&M’s...While participants were not told that their intake was going to be measured, some participants may have anticipated this which may have in turn influenced their eating behaviors.". Comment: Blinding of study participants was not attempted with respect to this outcome but this is not judged likely to introduce risk of performance bias in the current context. Participants were probed for awareness of the manipulation after completing the study and only those participants who were aware of the manipulation were included in the analysis. (Quote: "Results of the manipulation check revealed that 175 out of 224 participants accurately identified whether they were presented with low-fat- versus regular-labeled M&M’s and whether they had caloric information available or not. Therefore, only results for the 175 participants who accurately recalled both of their experimental conditions are presented."). Very unlikely that key study personnel were blinded, but the review authors judge that the outcome is not likely to be influenced by lack of blinding of key study personnel. |
| Selection: Blinding of outcome assessment (detection bias) | Not measured |  |
| Consumption: Blinding of outcome assessment (detection bias) | Low | Comment: No blinding of outcome assessment, but the review authors judge that the outcome measurement is not likely to be influenced by lack of blinding. |
| Intention to select or purchase: Blinding of outcome assessment (detection bias) | Not measured |  |
| Intention to consume: Blinding of outcome assessment (detection bias) | Not measured |  |
| Belief associated with consumption: Blinding of outcome assessment (detection bias) | Not measured |  |
| Appeal: Blinding of outcome assessment (detection bias) | Low | Comment: No blinding of outcome assessment, but the review authors judge that the outcome measurement is not likely to be influenced by lack of blinding. |
| Understanding of label: Blinding of outcome assessment (detection bias) | Low | Comment: No blinding of outcome assessment, but the review authors judge that the outcome measurement is not likely to be influenced by lack of blinding. |
| Selection: Incomplete outcome data (attrition bias) | Not measured |  |
| Consumption: Incomplete outcome data (attrition bias) | Low | Quote: "Results of the manipulation check revealed that 175 out of 224 participants accurately identified whether they were presented with low-fat- versus regular-labeled M&M’s and whether they had caloric information available or not. Therefore, only results for the 175 participants who accurately recalled both of their experimental conditions are presented." Comment: Reasons for excluding participants’ data from the analysis are provided in full. The review authors judge that the reason provided for these exclusions by the study authors is reasonable from the perspective of minimizing bias. |
| Intention to select or purchase: Incomplete outcome data (attrition bias) | Not measured |  |
| Intention to consume: Incomplete outcome data (attrition bias) | Not measured |  |
| Belief associated with consumption: Incomplete outcome data (attrition bias) | Not measured |  |
| Appeal: Incomplete outcome data (attrition bias) | Low | Quote: "Results of the manipulation check revealed that 175 out of 224 participants accurately identified whether they were presented with low-fat- versus regular-labeled M&M’s and whether they had caloric information available or not. Therefore, only results for the 175 participants who accurately recalled both of their experimental conditions are presented." Comment: Reasons for excluding participants’ data from the analysis are provided in full. The review authors judge that the reason provided for these exclusions by the study authors is reasonable from the perspective of minimizing bias. |
| Understanding of label: Incomplete outcome data (attrition bias) | Low | Quote: "Results of the manipulation check revealed that 175 out of 224 participants accurately identified whether they were presented with low-fat- versus regular-labeled M&M’s and whether they had caloric information available or not. Therefore, only results for the 175 participants who accurately recalled both of their experimental conditions are presented." Comment: Reasons for excluding participants’ data from the analysis are provided in full. The review authors judge that the reason provided for these exclusions by the study authors is reasonable from the perspective of minimizing bias. |
| Selective reporting (reporting bias) | Unclear | Comment: No evidence of selective outcome reporting based on study report. Search for record(s) containing details of study protocol conducted in ClinicalTrials.gov and the WHO International Clinical Trials Registry Platform (ICTRP) identified no record. Insufficient information to permit judgement of ‘Low risk’ or ‘High risk’. |
| Other bias | Low | Quote: “ANOVAs were performed to assess whether the four conditions were successfully randomized by age, BMI, ratings of hunger, and mean scores on the EAT-26 and DEBQ-R. No significant differences were observed between experimental groups (all ps > .1).” Comment: Evidence of no baseline imbalances between the relevant comparison groups on tested trait and state characteristics. |
| Selection: Summary risk of bias | Not measured |  |
| Consumption: Summary risk of bias | Unclear | Comment: Unclear risk of selection bias. |
| Intention to select or purchase: Summary risk of bias | Not measured |  |
| Intention to consume: Summary risk of bias | Not measured |  |
| Belief associated with consumption: Summary risk of bias | Not measured |  |
| Appeal: Summary risk of bias | Unclear | Comment: Unclear risk of selection bias. |
| Understanding of label: Summary risk of bias | Unclear | Comment: Unclear risk of selection bias. |

French et al., 2001 [*Published data only*].

| **Bias** | **Authors' judgement** | **Support for judgement** |
| --- | --- | --- |
| Random sequence generation (selection bias) | Low | Quote: “Four levels of pricing and 3 levels of promotion were examined in a Latin square design (Figure 1). The 4 levels of pricing were (1) equal price, (2) 10% price reduction for low-fat snacks, (3) 25% price reduction, and (4) 50% price reduction. The 3 levels of promotion were (1) no signs, (2) signs labeling low-fat snacks, and (3) signs labeling low-fat snacks combined with signs placed on vending machines encouraging a low-fat snack choice. The overall design was a 2 (setting: workplace or school) × 4 (pricing: equal, 10% reduction, 25% reduction, 50% reduction) × 3 (promotion: none, label only, label plus sign) factorial. Each of the 12 treatment conditions shown in Figure 1 was implemented at each of the 24 sites in a randomly assigned sequence in such a way that period effects (if any) were balanced over experimental conditions during each month.” Comment: Method of sequence generation (order of exposures) is not fully described, but the review authors judge that the random sequence was implemented in order to safeguard against risk of bias due to period effects, and is unlikely to have resulted in systematic differences between baseline characteristics of the groups that are compared. |
| Allocation concealment (selection bias) | 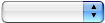 Low | Quote: “Four levels of pricing and 3 levels of promotion were examined in a Latin square design (Figure 1). The 4 levels of pricing were (1) equal price, (2) 10% price reduction for low-fat snacks, (3) 25% price reduction, and (4) 50% price reduction. The 3 levels of promotion were (1) no signs, (2) signs labeling low-fat snacks, and (3) signs labeling low-fat snacks combined with signs placed on vending machines encouraging a low-fat snack choice. The overall design was a 2 (setting: workplace or school) × 4 (pricing: equal, 10% reduction, 25% reduction, 50% reduction) × 3 (promotion: none, label only, label plus sign) factorial. Each of the 12 treatment conditions shown in Figure 1 was implemented at each of the 24 sites in a randomly assigned sequence in such a way that period effects (if any) were balanced over experimental conditions during each month.” Comment: The allocation sequence (order of exposures) was not concealed, but the review authors judge that the random sequence was implemented in order to safeguard against risk of bias due to period effects, and is unlikely to have resulted in systematic differences between baseline characteristics of the groups that are compared. |
| Selection: Blinding of participants and personnel (performance bias) | Low | Comment: No blinding or incomplete blinding of study participants but this is not judged likely to introduce risk of performance bias in the current context. Very unlikely that key study personnel were blinded, but the review authors judge that the outcome is not likely to be influenced by lack of blinding of key study personnel. |
| Consumption: Blinding of participants and personnel (performance bias) | Not measured |  |
| Intention to select or purchase: Blinding of participants and personnel (performance bias) | Not measured |  |
| Intention to consume: Blinding of participants and personnel (performance bias) | Not measured |  |
| Belief associated with consumption: Blinding of participants and personnel (performance bias) | Not measured |  |
| Appeal: Blinding of participants and personnel (performance bias) | Not measured |  |
| Understanding of label: Blinding of participants and personnel (performance bias) | Not measured |  |
| Selection: Blinding of outcome assessment (detection bias) | Low | Comment: No blinding of outcome assessment, but the review authors judge that the outcome measurement is not likely to be influenced by lack of blinding. |
| Consumption: Blinding of outcome assessment (detection bias) | Not measured |  |
| Intention to select or purchase: Blinding of outcome assessment (detection bias) | Not measured |  |
| Intention to consume: Blinding of outcome assessment (detection bias) | Not measured |  |
| Belief associated with consumption: Blinding of outcome assessment (detection bias) | Not measured |  |
| Appeal: Blinding of outcome assessment (detection bias) |  |  |
| Understanding of label: Blinding of outcome assessment (detection bias) | Not measured |  |
| Selection: Incomplete outcome data (attrition bias) | Low | Comment: No missing outcome data for selection outcome. |
| Consumption: Incomplete outcome data (attrition bias) | Not measured |  |
| Intention to select or purchase: Incomplete outcome data (attrition bias) | Not measured |  |
| Intention to consume: Incomplete outcome data (attrition bias) | Not measured |  |
| Belief associated with consumption: Incomplete outcome data (attrition bias) | Not measured |  |
| Appeal: Incomplete outcome data (attrition bias) | Not measured |  |
| Understanding of label: Incomplete outcome data (attrition bias) | Not measured |  |
| Selective reporting (reporting bias) | Unclear | Comment: No evidence of selective outcome reporting based on study report. Search for record(s) containing details of study protocol conducted in ClinicalTrials.gov and the WHO International Clinical Trials Registry Platform (ICTRP) identified no record. Insufficient information to permit judgement of ‘Low risk’ or ‘High risk’. |
| Other bias | Low | Comment: Study is a cluster randomised controlled trial with a crossover design. |
| Selection: Summary risk of bias | Low | Comment: Judged at low risk of selection bias, attrition bias and other bias. |
| Consumption: Summary risk of bias | Not measured |  |
| Intention to select or purchase: Summary risk of bias | Not measured |  |
| Intention to consume: Summary risk of bias | Not measured |  |
| Belief associated with consumption: Summary risk of bias | Not measured |  |
| Appeal: Summary risk of bias | Not measured |  |
| Understanding of label: Summary risk of bias | Not measured |  |

Kähkönen, Tuorila, & Lawless, 1997 [*Published data only*].

| **Bias** | **Authors' judgement** | **Support for judgement** |
| --- | --- | --- |
| Random sequence generation (selection bias) | Unclear | Comment: Insufficient information about the sequence generation process to permit judgement of ‘Low risk’ or ‘High risk’. |
| Allocation concealment (selection bias) | Unclear | Comment: Insufficient information concerning method of concealment to permit judgement of ‘Low risk’ or ‘High risk’. |
| Selection: Blinding of participants and personnel (performance bias) | Not measured |  |
| Consumption: Blinding of participants and personnel (performance bias) | Not measured |  |
| Intention to select or purchase: Blinding of participants and personnel (performance bias) | Not measured |  |
| Intention to consume: Blinding of participants and personnel (performance bias) | Not measured |  |
| Belief associated with consumption: Blinding of participants and personnel (performance bias) | Not measured |  |
| Appeal: Blinding of participants and personnel (performance bias) | Low | Quote: "The three information conditions were conducted in the same testing area at the same time but in such a way that the subjects could see only their own information." Comment: Blinding of study participants attempted and unlikely to have been broken - the review authors judge that the outcome is not likely to be influenced by lack of blinding of study participants in this case. Very unlikely that key study personnel were blinded, but the review authors judge that the outcome is not likely to be influenced by lack of blinding of key study personnel. |
| Understanding of label: Blinding of participants and personnel (performance bias) | Not measured |  |
| Selection: Blinding of outcome assessment (detection bias) | Not measured |  |
| Consumption: Blinding of outcome assessment (detection bias) | Not measured |  |
| Intention to select or purchase: Blinding of outcome assessment (detection bias) | Not measured |  |
| Intention to consume: Blinding of outcome assessment (detection bias) | Not measured |  |
| Belief associated with consumption: Blinding of outcome assessment (detection bias) | Not measured |  |
| Appeal: Blinding of outcome assessment (detection bias) | Low | Comment: No blinding of outcome assessment, but the review authors judge that the outcome measurement is not likely to be influenced by lack of blinding. |
| Understanding of label: Blinding of outcome assessment (detection bias) | Not measured |  |
| Selection: Incomplete outcome data (attrition bias) | Not measured |  |
| Consumption: Incomplete outcome data (attrition bias) | Not measured |  |
| Intention to select or purchase: Incomplete outcome data (attrition bias) | Not measured |  |
| Intention to consume: Incomplete outcome data (attrition bias) | Not measured |  |
| Belief associated with consumption: Incomplete outcome data (attrition bias) | Not measured |  |
| Appeal: Incomplete outcome data (attrition bias) | Low | Comment: No missing outcome data for product appeal outcome. |
| Understanding of label: Incomplete outcome data (attrition bias) | Not measured |  |
| Selective reporting (reporting bias) | Unclear | Comment: No evidence of selective outcome reporting based on study report. Search for record(s) containing details of study protocol conducted in ClinicalTrials.gov and the WHO International Clinical Trials Registry Platform (ICTRP) identified no record. Insufficient information to permit judgement of ‘Low risk’ or ‘High risk’. |
| Other bias | Unclear | Comment: Baseline comparability between comparison groups is not reported. Insufficient information to permit judgement of ‘Low risk’ or ‘High risk’. |
| Selection: Summary risk of bias | Not measured |  |
| Consumption: Summary risk of bias | Not measured |  |
| Intention to select or purchase: Summary risk of bias | Not measured |  |
| Intention to consume: Summary risk of bias | Not measured |  |
| Belief associated with consumption: Summary risk of bias | Not measured |  |
| Appeal: Summary risk of bias | Other bias | Comment: Unclear risk of selection and unclear risk of other bias (baseline comparability between groups). |
| Understanding of label: Summary risk of bias | Not measured |  |

Kähkönen, Hakanpää, & Tuorila, 1999a [*Published data only*].

Comparison 1: Chocolate.

| **Bias** | **Authors' judgement** | **Support for judgement** |
| --- | --- | --- |
| Random sequence generation (selection bias) | Unclear | Comment: Insufficient information about the sequence generation process to permit judgement of ‘Low risk’ or ‘High risk’. |
| Allocation concealment (selection bias) | Unclear | Comment: Insufficient information concerning method of concealment to permit judgement of ‘Low risk’ or ‘High risk’. |
| Selection: Blinding of participants and personnel (performance bias) | Not measured |  |
| Consumption: Blinding of participants and personnel (performance bias) | Not measured |  |
| Intention to select or purchase: Blinding of participants and personnel (performance bias) | Not measured |  |
| Intention to consume: Blinding of participants and personnel (performance bias) | Not measured |  |
| Belief associated with consumption: Blinding of participants and personnel (performance bias) | Not measured |  |
| Appeal: Blinding of participants and personnel (performance bias) | Low | Quote: "Seven other products were also rated in a blind rating (two regular-fat chocolate bars, a regular-fat frankfurter, two types of cheese and two types of juice). The purpose of these additional samples was to minimize the subjects’ possibilities to make associations between different sessions during the study. The results of these samples were not included in data analysis...All the ratings were done in individual tasting booths." Comment: Blinding of study participants attempted and unlikely to have been broken - the review authors judge that the outcome is not likely to be influenced by lack of blinding of study participants in this case. Very unlikely that key study personnel were blinded, but the review authors judge that the outcome is not likely to be influenced by lack of blinding of key study personnel. |
| Understanding of label: Blinding of participants and personnel (performance bias) | Not measured |  |
| Selection: Blinding of outcome assessment (detection bias) | Not measured |  |
| Consumption: Blinding of outcome assessment (detection bias) | Not measured |  |
| Intention to select or purchase: Blinding of outcome assessment (detection bias) | Not measured |  |
| Intention to consume: Blinding of outcome assessment (detection bias) | Not measured |  |
| Belief associated with consumption: Blinding of outcome assessment (detection bias) | Not measured |  |
| Appeal: Blinding of outcome assessment (detection bias) | Low | Comment: No blinding of outcome assessment, but the review authors judge that the outcome measurement is not likely to be influenced by lack of blinding. |
| Understanding of label: Blinding of outcome assessment (detection bias) | Not measured |  |
| Selection: Incomplete outcome data (attrition bias) | Not measured |  |
| Consumption: Incomplete outcome data (attrition bias) | Not measured |  |
| Intention to select or purchase: Incomplete outcome data (attrition bias) | Not measured |  |
| Intention to consume: Incomplete outcome data (attrition bias) | Not measured |  |
| Belief associated with consumption: Incomplete outcome data (attrition bias) | Not measured |  |
| Appeal: Incomplete outcome data (attrition bias) | Low | Comment: No missing outcome data for product appeal outcome. |
| Understanding of label: Incomplete outcome data (attrition bias) | Not measured |  |
| Selective reporting (reporting bias) | Unclear | Comment: No evidence of selective outcome reporting based on study report. Search for record(s) containing details of study protocol conducted in ClinicalTrials.gov and the WHO International Clinical Trials Registry Platform (ICTRP) identified no record. Insufficient information to permit judgement of ‘Low risk’ or ‘High risk’. |
| Other bias | Unclear | Comment: Baseline comparability between comparison groups is not reported. Insufficient information to permit judgement of ‘Low risk’ or ‘High risk’. |
| Selection: Summary risk of bias | Not measured |  |
| Consumption: Summary risk of bias | Not measured |  |
| Intention to select or purchase: Summary risk of bias | Not measured |  |
| Intention to consume: Summary risk of bias | Not measured |  |
| Belief associated with consumption: Summary risk of bias | Not measured |  |
| Appeal: Summary risk of bias | Unclear | Comment: Unclear risk of selection bias and other bias (baseline comparability between groups). |
| Understanding of label: Summary risk of bias | Not measured |  |

Kähkönen, Hakanpää, & Tuorila, 1999a [*Published data only*].

Comparison 1: Sausage.

| **Bias** | **Authors' judgement** | **Support for judgement** |
| --- | --- | --- |
| Random sequence generation (selection bias) | Unclear | Comment: Insufficient information about the sequence generation process to permit judgement of ‘Low risk’ or ‘High risk’. |
| Allocation concealment (selection bias) | Unclear | Comment: Insufficient information concerning method of concealment to permit judgement of ‘Low risk’ or ‘High risk’. |
| Selection: Blinding of participants and personnel (performance bias) | Not measured |  |
| Consumption: Blinding of participants and personnel (performance bias) | Not measured |  |
| Intention to select or purchase: Blinding of participants and personnel (performance bias) | Not measured |  |
| Intention to consume: Blinding of participants and personnel (performance bias) | Not measured |  |
| Belief associated with consumption: Blinding of participants and personnel (performance bias) | Not measured |  |
| Appeal: Blinding of participants and personnel (performance bias) | Low | Quote: "The three information conditions were conducted in the same testing area at the same time but in such a way that the subjects could see only their own information." Comment: Blinding of study participants attempted and unlikely to have been broken - the review authors judge that the outcome is not likely to be influenced by lack of blinding of study participants in this case. Very unlikely that key study personnel were blinded, but the review authors judge that the outcome is not likely to be influenced by lack of blinding of key study personnel. |
| Understanding of label: Blinding of participants and personnel (performance bias) | Not measured |  |
| Selection: Blinding of outcome assessment (detection bias) | Not measured |  |
| Consumption: Blinding of outcome assessment (detection bias) | Not measured |  |
| Intention to select or purchase: Blinding of outcome assessment (detection bias) | Not measured |  |
| Intention to consume: Blinding of outcome assessment (detection bias) | Not measured |  |
| Belief associated with consumption: Blinding of outcome assessment (detection bias) | Not measured |  |
| Appeal: Blinding of outcome assessment (detection bias) | Low | Comment: No blinding of outcome assessment, but the review authors judge that the outcome measurement is not likely to be influenced by lack of blinding. |
| Understanding of label: Blinding of outcome assessment (detection bias) | Not measured |  |
| Selection: Incomplete outcome data (attrition bias) | Not measured |  |
| Consumption: Incomplete outcome data (attrition bias) | Not measured |  |
| Intention to select or purchase: Incomplete outcome data (attrition bias) | Not measured |  |
| Intention to consume: Incomplete outcome data (attrition bias) | Not measured |  |
| Belief associated with consumption: Incomplete outcome data (attrition bias) | Not measured |  |
| Appeal: Incomplete outcome data (attrition bias) | Low | Comment: No missing outcome data for product appeal outcome. |
| Understanding of label: Incomplete outcome data (attrition bias) | Not measured |  |
| Selective reporting (reporting bias) | Unclear | Comment: No evidence of selective outcome reporting based on study report. Search for record(s) containing details of study protocol conducted in ClinicalTrials.gov and the WHO International Clinical Trials Registry Platform (ICTRP) identified no record. Insufficient information to permit judgement of ‘Low risk’ or ‘High risk’. |
| Other bias | Unclear | Comment: Baseline comparability between comparison groups is not reported. Insufficient information to permit judgement of ‘Low risk’ or ‘High risk’. |
| Selection: Summary risk of bias | Not measured |  |
| Consumption: Summary risk of bias | Not measured |  |
| Intention to select or purchase: Summary risk of bias | Not measured |  |
| Intention to consume: Summary risk of bias | Not measured |  |
| Belief associated with consumption: Summary risk of bias | Not measured |  |
| Appeal: Summary risk of bias | Other bias | Comment: Unclear risk of selection and unclear risk of other bias (baseline comparability between groups). |
| Understanding of label: Summary risk of bias | Not measured |  |

Kruja, 2014 [*Published data only*].

| **Bias** | **Authors' judgement** | **Support for judgement** |
| --- | --- | --- |
| Random sequence generation (selection bias) | Unclear | Comment: Insufficient information about the sequence generation process (order of exposures) to permit judgement of ‘Low risk’ or ‘High risk’. |
| Allocation concealment (selection bias) | Unclear | Comment: Insufficient information concerning method of concealment to permit judgement of ‘Low risk’ or ‘High risk’. |
| Selection: Blinding of participants and personnel (performance bias) | Not measured |  |
| Consumption: Blinding of participants and personnel (performance bias) | Low | Comment: No blinding of study participants attempted but this is not judged likely to introduce risk of performance bias in the current context. Very unlikely that key study personnel were blinded, but the review authors judge that the outcome is not likely to be influenced by lack of blinding of key study personnel. |
| Intention to select or purchase: Blinding of participants and personnel (performance bias) | Not measured |  |
| Intention to consume: Blinding of participants and personnel (performance bias) | Not measured |  |
| Belief associated with consumption: Blinding of participants and personnel (performance bias) | Not measured |  |
| Appeal: Blinding of participants and personnel (performance bias) | Low | Comment: Blinding of study participants was not attempted with respect to this outcome but this is not judged likely to introduce risk of performance bias in the current context. Very unlikely that key study personnel were blinded, but the review authors judge that the outcome is not likely to be influenced by lack of blinding of key study personnel. |
| Understanding of label: Blinding of participants and personnel (performance bias) | Low | Comment: Blinding of study participants was not attempted with respect to this outcome but this is not judged likely to introduce risk of performance bias in the current context. Very unlikely that key study personnel were blinded, but the review authors judge that the outcome is not likely to be influenced by lack of blinding of key study personnel. |
| Selection: Blinding of outcome assessment (detection bias) | Not measured |  |
| Consumption: Blinding of outcome assessment (detection bias) | Low | Comment: No blinding of outcome assessment, but the review authors judge that the outcome measurement is not likely to be influenced by lack of blinding. |
| Intention to select or purchase: Blinding of outcome assessment (detection bias) | Not measured |  |
| Intention to consume: Blinding of outcome assessment (detection bias) | Not measured |  |
| Belief associated with consumption: Blinding of outcome assessment (detection bias) | Not measured |  |
| Appeal: Blinding of outcome assessment (detection bias) | Low | Comment: No blinding of outcome assessment, but the review authors judge that the outcome measurement is not likely to be influenced by lack of blinding. |
| Understanding of label: Blinding of outcome assessment (detection bias) | Low | Comment: No blinding of outcome assessment, but the review authors judge that the outcome measurement is not likely to be influenced by lack of blinding. |
| Selection: Incomplete outcome data (attrition bias) | Not measured |  |
| Consumption: Incomplete outcome data (attrition bias) | Low | Quote: "Twenty-seven participants were excluded from the analyses because they were outside the age range of a typical college student (n = 3), did not understand or comply with the instructions of the study (n = 6), were lactose intolerant (n = 1), were obese (n = 5), smoked more than 20 cigarettes per week (n = 3), or were aware that the two drinks provided were identical (n = 9)." Comment: Reasons for excluding participants’ data from the analysis are provided in full. The review authors judge that the majority of exclusions of participants from the analysis by study authors are in line with pre-specified study eligibility. In addition, nine participants were excluded due to their awareness that the drinks had been identical between conditions, which the review authors judge is reasonable from the perspective of minimizing bias. |
| Intention to select or purchase: Incomplete outcome data (attrition bias) | Not measured |  |
| Intention to consume: Incomplete outcome data (attrition bias) | Not measured |  |
| Belief associated with consumption: Incomplete outcome data (attrition bias) | Not measured |  |
| Appeal: Incomplete outcome data (attrition bias) | Low | Quote: "Twenty-seven participants were excluded from the analyses because they were outside the age range of a typical college student (n = 3), did not understand or comply with the instructions of the study (n = 6), were lactose intolerant (n = 1), were obese (n = 5), smoked more than 20 cigarettes per week (n = 3), or were aware that the two drinks provided were identical (n = 9)." Comment: Reasons for excluding participants’ data from the analysis are provided in full. The review authors judge that the majority of exclusions of participants from the analysis by study authors are in line with pre-specified study eligibility. In addition, nine participants were excluded due to their awareness that the drinks had been identical between conditions, which the review authors judge is reasonable from the perspective of minimizing bias. |
| Understanding of label: Incomplete outcome data (attrition bias) | Low | Quote: "Twenty-seven participants were excluded from the analyses because they were outside the age range of a typical college student (n = 3), did not understand or comply with the instructions of the study (n = 6), were lactose intolerant (n = 1), were obese (n = 5), smoked more than 20 cigarettes per week (n = 3), or were aware that the two drinks provided were identical (n = 9)." Comment: Reasons for excluding participants’ data from the analysis are provided in full. The review authors judge that the majority of exclusions of participants from the analysis by study authors are in line with pre-specified study eligibility. In addition, nine participants were excluded due to their awareness that the drinks had been identical between conditions, which the review authors judge is reasonable from the perspective of minimizing bias. |
| Selective reporting (reporting bias) | Unclear | Comment: No evidence of selective outcome reporting based on study report. Search for record(s) containing details of study protocol conducted in ClinicalTrials.gov and the WHO International Clinical Trials Registry Platform (ICTRP) identified no record. Insufficient information to permit judgement of ‘Low risk’ or ‘High risk’. |
| Other bias | Low | Comment: Study is an individually randomised controlled trial with a crossover design. |
| Selection: Summary risk of bias | Not measured |  |
| Consumption: Summary risk of bias | Unclear | Comment: Unclear risk of selection bias. |
| Intention to select or purchase: Summary risk of bias | Not measured |  |
| Intention to consume: Summary risk of bias | Not measured |  |
| Belief associated with consumption: Summary risk of bias | Not measured |  |
| Appeal: Summary risk of bias | Unclear | Comment: Unclear risk of selection bias. |
| Understanding of label: Summary risk of bias | Unclear | Comment: Unclear risk of selection bias. |

Norton, Fryer, & Parkinson, 2013 [*Published data only*].

| **Bias** | **Authors' judgement** | **Support for judgement** |
| --- | --- | --- |
| Random sequence generation (selection bias) | Unclear | Comment: Insufficient information about the sequence generation process (order of exposures) to permit judgement of ‘Low risk’ or ‘High risk’. |
| Allocation concealment (selection bias) | Unclear | Comment: Insufficient information concerning method of concealment to permit judgement of ‘Low risk’ or ‘High risk’. |
| Selection: Blinding of participants and personnel (performance bias) | Not measured |  |
| Consumption: Blinding of participants and personnel (performance bias) | Not measured |  |
| Intention to select or purchase: Blinding of participants and personnel (performance bias) | Not measured |  |
| Intention to consume: Blinding of participants and personnel (performance bias) | Not measured |  |
| Belief associated with consumption: Blinding of participants and personnel (performance bias) | Not measured |  |
| Appeal: Blinding of participants and personnel (performance bias) | Low | Quote: "The order in which the samples were tasted was counterbalanced for participants between the first and second session. Significant effects of session order (p-values <.05) were not observed for expected liking [F (1, 85) = .508, p = .478] or actual liking [F (1, 85) = .802, p = .373], suggesting that any carryover effects were minimal." Comment: Blinding of study participants was not attempted with respect to this outcome. The study authors report testing for order effects and finding none. Non-blinding of participants is not judged likely to introduce risk of performance bias in the current context. Very unlikely that key study personnel were blinded, but the review authors judge that the outcome is not likely to be influenced by lack of blinding of key study personnel. |
| Understanding of label: Blinding of participants and personnel (performance bias) | Not measured |  |
| Selection: Blinding of outcome assessment (detection bias) | Not measured |  |
| Consumption: Blinding of outcome assessment (detection bias) | Not measured |  |
| Intention to select or purchase: Blinding of outcome assessment (detection bias) | Not measured |  |
| Intention to consume: Blinding of outcome assessment (detection bias) | Not measured |  |
| Belief associated with consumption: Blinding of outcome assessment (detection bias) | Not measured |  |
| Appeal: Blinding of outcome assessment (detection bias) | Low | Comment: No blinding of outcome assessment, but the review authors judge that the outcome measurement is not likely to be influenced by lack of blinding. |
| Understanding of label: Blinding of outcome assessment (detection bias) | Not measured |  |
| Selection: Incomplete outcome data (attrition bias) | Not measured |  |
| Consumption: Incomplete outcome data (attrition bias) | Not measured |  |
| Intention to select or purchase: Incomplete outcome data (attrition bias) | Not measured |  |
| Intention to consume: Incomplete outcome data (attrition bias) | Not measured |  |
| Belief associated with consumption: Incomplete outcome data (attrition bias) | Not measured |  |
| Appeal: Incomplete outcome data (attrition bias) | Low | Quote: "Undergraduate and postgraduate students and staff from The University of Birmingham were recruited via a flyer and poster advertising campaign. One hundred signed up, 91 attended the first session, and 87 completed both sessions." Comment: Outcome data from 13 recruited participants who failed to attend one or both study sessions were excluded from the analysis. This study has a crossover design, so risk of bias due to any difference in the proportion of incomplete outcome data (or in distributions of different reasons for missing data) between comparison groups is not applicable. |
| Understanding of label: Incomplete outcome data (attrition bias) | Not measured |  |
| Selective reporting (reporting bias) | Unclear | Comment: No evidence of selective outcome reporting based on study report. Search for record(s) containing details of study protocol conducted in ClinicalTrials.gov and the WHO International Clinical Trials Registry Platform (ICTRP) identified no record. Insufficient information to permit judgement of ‘Low risk’ or ‘High risk’. |
| Other bias | Low | Comment: Study is an individually randomised controlled trial with a crossover design. |
| Selection: Summary risk of bias | Not measured |  |
| Consumption: Summary risk of bias | Not measured |  |
| Intention to select or purchase: Summary risk of bias | Not measured |  |
| Intention to consume: Summary risk of bias | Not measured |  |
| Belief associated with consumption: Summary risk of bias | Not measured |  |
| Appeal: Summary risk of bias | Unclear | Comment: Unclear risk of selection bias. |
| Understanding of label: Summary risk of bias | Not measured |  |

Stubenitsky, Aaron, Catt, & Mela, 1999 [*Published data only*].

Comparison 1: Chocolate.

| **Bias** | **Authors' judgement** | **Support for judgement** |
| --- | --- | --- |
| Random sequence generation (selection bias) | Unclear | Comment: Insufficient information about the sequence generation process to permit judgement of ‘Low risk’ or ‘High risk’. |
| Allocation concealment (selection bias) | Unclear | Comment: Insufficient information concerning method of concealment to permit judgement of ‘Low risk’ or ‘High risk’. |
| Selection: Blinding of participants and personnel (performance bias) | Not measured |  |
| Consumption: Blinding of participants and personnel (performance bias) | Not measured |  |
| Intention to select or purchase: Blinding of participants and personnel (performance bias) | Not measured |  |
| Intention to consume: Blinding of participants and personnel (performance bias) | Not measured |  |
| Belief associated with consumption: Blinding of participants and personnel (performance bias) | Not measured |  |
| Appeal: Blinding of participants and personnel (performance bias) | Low | Comment: Blinding of study participants attempted and unlikely to have been broken - the review authors judge that the outcome is not likely to be influenced by lack of blinding of study participants in this case. Very unlikely that key study personnel were blinded, but the review authors judge that the outcome is not likely to be influenced by lack of blinding of key study personnel. |
| Understanding of label: Blinding of participants and personnel (performance bias) | Not measured |  |
| Selection: Blinding of outcome assessment (detection bias) | Not measured |  |
| Consumption: Blinding of outcome assessment (detection bias) | Not measured |  |
| Intention to select or purchase: Blinding of outcome assessment (detection bias) | Not measured |  |
| Intention to consume: Blinding of outcome assessment (detection bias) | Not measured |  |
| Belief associated with consumption: Blinding of outcome assessment (detection bias) | Not measured |  |
| Appeal: Blinding of outcome assessment (detection bias) | Low | Comment: No blinding of outcome assessment, but the review authors judge that the outcome measurement is not likely to be influenced by lack of blinding. |
| Understanding of label: Blinding of outcome assessment (detection bias) | Not measured |  |
| Selection: Incomplete outcome data (attrition bias) | Not measured |  |
| Consumption: Incomplete outcome data (attrition bias) | Not measured |  |
| Intention to select or purchase: Incomplete outcome data (attrition bias) | Not measured |  |
| Intention to consume: Incomplete outcome data (attrition bias) | Not measured |  |
| Belief associated with consumption: Incomplete outcome data (attrition bias) | Not measured |  |
| Appeal: Incomplete outcome data (attrition bias) | Low | Comment: No missing outcome data for product appeal outcome. |
| Understanding of label: Incomplete outcome data (attrition bias) | Not measured |  |
| Selective reporting (reporting bias) | High | Comment: No results appear to have been reported for one of the in-home rating measures: 'fillingness'. Search for record(s) containing details of study protocol conducted in ClinicalTrials.gov and the WHO International Clinical Trials Registry Platform (ICTRP) identified no record. Judgement based on Cochrane Handbook guidance is ‘High risk’. |
| Other bias | Low | Quote: "There were no significant group differences in age or BMI (pre- or post-study). There was a substantial higher proportion of females than males (about 75 vs 25%), but this did not differ between groups." Comment: Evidence of no baseline imbalances between the relevant comparison groups on tested trait and state characteristics. |
| Selection: Summary risk of bias | Not measured |  |
| Consumption: Summary risk of bias | Not measured |  |
| Intention to select or purchase: Summary risk of bias | Not measured |  |
| Intention to consume: Summary risk of bias | Not measured |  |
| Belief associated with consumption: Summary risk of bias | Not measured |  |
| Appeal: Summary risk of bias | Unclear | Comment: Unclear risk of selection bias. |
| Understanding of label: Summary risk of bias | Not measured |  |

Stubenitsky, Aaron, Catt, & Mela, 1999 [*Published data only*].

Comparison 2: Sausage.

| **Bias** | **Authors' judgement** | **Support for judgement** |
| --- | --- | --- |
| Random sequence generation (selection bias) | Unclear | Comment: Insufficient information about the sequence generation process to permit judgement of ‘Low risk’ or ‘High risk’. |
| Allocation concealment (selection bias) | Unclear | Comment: Insufficient information concerning method of concealment to permit judgement of ‘Low risk’ or ‘High risk’. |
| Selection: Blinding of participants and personnel (performance bias) | Not measured |  |
| Consumption: Blinding of participants and personnel (performance bias) | Not measured |  |
| Intention to select or purchase: Blinding of participants and personnel (performance bias) | Not measured |  |
| Intention to consume: Blinding of participants and personnel (performance bias) | Not measured |  |
| Belief associated with consumption: Blinding of participants and personnel (performance bias) | Not measured |  |
| Appeal: Blinding of participants and personnel (performance bias) | Low | Comment: Blinding of study participants attempted and unlikely to have been broken - the review authors judge that the outcome is not likely to be influenced by lack of blinding of study participants in this case. Very unlikely that key study personnel were blinded, but the review authors judge that the outcome is not likely to be influenced by lack of blinding of key study personnel. |
| Understanding of label: Blinding of participants and personnel (performance bias) | Not measured |  |
| Selection: Blinding of outcome assessment (detection bias) | Not measured |  |
| Consumption: Blinding of outcome assessment (detection bias) | Not measured |  |
| Intention to select or purchase: Blinding of outcome assessment (detection bias) | Not measured |  |
| Intention to consume: Blinding of outcome assessment (detection bias) | Not measured |  |
| Belief associated with consumption: Blinding of outcome assessment (detection bias) | Not measured |  |
| Appeal: Blinding of outcome assessment (detection bias) | Low | Comment: No blinding of outcome assessment, but the review authors judge that the outcome measurement is not likely to be influenced by lack of blinding. |
| Understanding of label: Blinding of outcome assessment (detection bias) | Not measured |  |
| Selection: Incomplete outcome data (attrition bias) | Not measured |  |
| Consumption: Incomplete outcome data (attrition bias) | Not measured |  |
| Intention to select or purchase: Incomplete outcome data (attrition bias) | Not measured |  |
| Intention to consume: Incomplete outcome data (attrition bias) | Not measured |  |
| Belief associated with consumption: Incomplete outcome data (attrition bias) | Not measured |  |
| Appeal: Incomplete outcome data (attrition bias) | Low | Comment: No missing outcome data for product appeal outcome. |
| Understanding of label: Incomplete outcome data (attrition bias) | Not measured |  |
| Selective reporting (reporting bias) | High | Comment: No results appear to have been reported for one of the in-home rating measures: 'fillingness'. Search for record(s) containing details of study protocol conducted in ClinicalTrials.gov and the WHO International Clinical Trials Registry Platform (ICTRP) identified no record. Judgement based on Cochrane Handbook guidance is ‘High risk’. |
| Other bias | Low | Quote: "There were no significant group differences in age or BMI (pre- or post-study). There was a substantial higher proportion of females than males (about 75 vs 25%), but this did not differ between groups." Comment: Evidence of no baseline imbalances between the relevant comparison groups on tested trait and state characteristics. |
| Selection: Summary risk of bias | Not measured |  |
| Consumption: Summary risk of bias | Not measured |  |
| Intention to select or purchase: Summary risk of bias | Not measured |  |
| Intention to consume: Summary risk of bias | Not measured |  |
| Belief associated with consumption: Summary risk of bias | Not measured |  |
| Appeal: Summary risk of bias | Unclear | Comment: Unclear risk of selection bias. |
| Understanding of label: Summary risk of bias | Not measured |  |

Wansink & Chandon 2006 (S2) [*Published data only*].
Comparison 1: Chocolate.

| **Bias** | **Authors' judgement** | **Support for judgement** |
| --- | --- | --- |
| Random sequence generation (selection bias) | Unclear | Comment: Insufficient information about the sequence generation process to permit judgement of ‘Low risk’ or ‘High risk’. |
| Allocation concealment (selection bias) | Unclear | Comment: Insufficient information concerning method of concealment to permit judgement of ‘Low risk’ or ‘High risk’. |
| Selection: Blinding of participants and personnel (performance bias) | Not measured |  |
| Consumption: Blinding of participants and personnel (performance bias) | Not measured |  |
| Intention to select or purchase: Blinding of participants and personnel (performance bias) | Not measured |  |
| Intention to consume: Blinding of participants and personnel (performance bias) | Not measured |  |
| Belief associated with consumption: Blinding of participants and personnel (performance bias) | Low | Comment: Blinding of study participants attempted and unlikely to have been broken - the review authors judge that the outcome is not likely to be influenced by lack of blinding of study participants in this case. Very unlikely that key study personnel were blinded, but the review authors judge that the outcome is not likely to be influenced by lack of blinding of key study personnel. |
| Appeal: Blinding of participants and personnel (performance bias) | Not measured |  |
| Understanding of label: Blinding of participants and personnel (performance bias) | Low | Comment: Blinding of study participants attempted and unlikely to have been broken - the review authors judge that the outcome is not likely to be influenced by lack of blinding of study participants in this case. Very unlikely that key study personnel were blinded, but the review authors judge that the outcome is not likely to be influenced by lack of blinding of key study personnel. |
| Selection: Blinding of outcome assessment (detection bias) | Not measured |  |
| Consumption: Blinding of outcome assessment (detection bias) | Not measured |  |
| Intention to select or purchase: Blinding of outcome assessment (detection bias) | Not measured |  |
| Intention to consume: Blinding of outcome assessment (detection bias) | Not measured |  |
| Belief associated with consumption: Blinding of outcome assessment (detection bias) | Low | Comment: No blinding of outcome assessment, but the review authors judge that the outcome measurement is not likely to be influenced by lack of blinding. |
| Appeal: Blinding of outcome assessment (detection bias) | Not measured |  |
| Understanding of label: Blinding of outcome assessment (detection bias) | Low | Comment: No blinding of outcome assessment, but the review authors judge that the outcome measurement is not likely to be influenced by lack of blinding. |
| Selection: Incomplete outcome data (attrition bias) | Not measured |  |
| Consumption: Incomplete outcome data (attrition bias) | Not measured |  |
| Intention to select or purchase: Incomplete outcome data (attrition bias) | Not measured |  |
| Intention to consume: Incomplete outcome data (attrition bias) | Not measured |  |
| Belief associated with consumption: Incomplete outcome data (attrition bias) | Unclear | Comment: Reasons for excluding participants’ data from the analysis are not provided. |
| Appeal: Incomplete outcome data (attrition bias) | Not measured |  |
| Understanding of label: Incomplete outcome data (attrition bias) | Unclear | Comment: Reasons for excluding participants’ data from the analysis are not provided. |
| Selective reporting (reporting bias) | Unclear | Comment: No evidence of selective outcome reporting based on study report. Search for record(s) containing details of study protocol conducted in ClinicalTrials.gov and the WHO International Clinical Trials Registry Platform (ICTRP) identified no record. Insufficient information to permit judgement of ‘Low risk’ or ‘High risk’. |
| Other bias | Unclear | Comment: Baseline comparability between comparison groups is not reported. Insufficient information to permit judgement of ‘Low risk’ or ‘High risk’. |
| Selection: Summary risk of bias | Not measured |  |
| Consumption: Summary risk of bias | Not measured |  |
| Intention to select or purchase: Summary risk of bias | Not measured |  |
| Intention to consume: Summary risk of bias | Not measured |  |
| Belief associated with consumption: Summary risk of bias | Unclear | Comment: Unclear risk of selection bias, attrition bias and other bias (baseline comparability between groups). |
| Appeal: Summary risk of bias | Not measured |  |
| Understanding of label: Summary risk of bias | Unclear | Comment: Unclear risk of selection bias, attrition bias and other bias (baseline comparability between groups). |

Wansink & Chandon 2006 (S2) [*Published data only*].
Comparison 2: Granola.

| **Bias** | **Authors' judgement** | **Support for judgement** |
| --- | --- | --- |
| Random sequence generation (selection bias) | Unclear | Comment: Insufficient information about the sequence generation process to permit judgement of ‘Low risk’ or ‘High risk’. |
| Allocation concealment (selection bias) | Unclear | Comment: Insufficient information concerning method of concealment to permit judgement of ‘Low risk’ or ‘High risk’. |
| Selection: Blinding of participants and personnel (performance bias) | Not measured |  |
| Consumption: Blinding of participants and personnel (performance bias) | Not measured |  |
| Intention to select or purchase: Blinding of participants and personnel (performance bias) | Not measured |  |
| Intention to consume: Blinding of participants and personnel (performance bias) | Not measured |  |
| Belief associated with consumption: Blinding of participants and personnel (performance bias) | Low | Comment: Blinding of study participants attempted and unlikely to have been broken - the review authors judge that the outcome is not likely to be influenced by lack of blinding of study participants in this case. Very unlikely that key study personnel were blinded, but the review authors judge that the outcome is not likely to be influenced by lack of blinding of key study personnel. |
| Appeal: Blinding of participants and personnel (performance bias) | Not measured |  |
| Understanding of label: Blinding of participants and personnel (performance bias) | Low | Comment: Blinding of study participants attempted and unlikely to have been broken - the review authors judge that the outcome is not likely to be influenced by lack of blinding of study participants in this case. Very unlikely that key study personnel were blinded, but the review authors judge that the outcome is not likely to be influenced by lack of blinding of key study personnel. |
| Selection: Blinding of outcome assessment (detection bias) | Not measured |  |
| Consumption: Blinding of outcome assessment (detection bias) | Not measured |  |
| Intention to select or purchase: Blinding of outcome assessment (detection bias) | Not measured |  |
| Intention to consume: Blinding of outcome assessment (detection bias) | Not measured |  |
| Belief associated with consumption: Blinding of outcome assessment (detection bias) | Low | Comment: No blinding of outcome assessment, but the review authors judge that the outcome measurement is not likely to be influenced by lack of blinding. |
| Appeal: Blinding of outcome assessment (detection bias) | Not measured |  |
| Understanding of label: Blinding of outcome assessment (detection bias) | Low | Comment: No blinding of outcome assessment, but the review authors judge that the outcome measurement is not likely to be influenced by lack of blinding. |
| Selection: Incomplete outcome data (attrition bias) | Not measured |  |
| Consumption: Incomplete outcome data (attrition bias) | Not measured |  |
| Intention to select or purchase: Incomplete outcome data (attrition bias) | Not measured |  |
| Intention to consume: Incomplete outcome data (attrition bias) | Not measured |  |
| Belief associated with consumption: Incomplete outcome data (attrition bias) | Unclear | Comment: Reasons for excluding participants’ data from the analysis are not provided. |
| Appeal: Incomplete outcome data (attrition bias) | Not measured |  |
| Understanding of label: Incomplete outcome data (attrition bias) | Unclear | Comment: Reasons for excluding participants’ data from the analysis are not provided. |
| Selective reporting (reporting bias) | Unclear | Comment: No evidence of selective outcome reporting based on study report. Search for record(s) containing details of study protocol conducted in ClinicalTrials.gov and the WHO International Clinical Trials Registry Platform (ICTRP) identified no record. Insufficient information to permit judgement of ‘Low risk’ or ‘High risk’. |
| Other bias | Unclear | Comment: Baseline comparability between comparison groups is not reported. Insufficient information to permit judgement of ‘Low risk’ or ‘High risk’. |
| Selection: Summary risk of bias | Not measured |  |
| Consumption: Summary risk of bias | Not measured |  |
| Intention to select or purchase: Summary risk of bias | Not measured |  |
| Intention to consume: Summary risk of bias | Not measured |  |
| Belief associated with consumption: Summary risk of bias | Unclear | Comment: Unclear risk of selection bias, attrition bias and other bias (baseline comparability between groups). |
| Appeal: Summary risk of bias | Not measured |  |
| Understanding of label: Summary risk of bias | Unclear | Comment: Unclear risk of selection bias, attrition bias and other bias (baseline comparability between groups). |

Wansink & Chandon 2006 (S3) [*Published data only*].

| **Bias** | **Authors' judgement** | **Support for judgement** |
| --- | --- | --- |
| Random sequence generation (selection bias) | Unclear | Comment: Insufficient information about the sequence generation process to permit judgement of ‘Low risk’ or ‘High risk’. |
| Allocation concealment (selection bias) | Unclear | Comment: Insufficient information concerning method of concealment to permit judgement of ‘Low risk’ or ‘High risk’. |
| Selection: Blinding of participants and personnel (performance bias) | Not measured |  |
| Consumption: Blinding of participants and personnel (performance bias) | Unclear | Quote: "The study was conducted over ten sessions that lasted from 3:30 to 5:00 P.M. on each of ten days (Tuesdays and Thursdays for five non-consecutive weeks). On arriving at the dimly lit theater, participants were seated in every other seat and asked to watch and rate a series of made-for-television movie previews and a 60-minute pilot show called “Hazard County.” They were also told that because it was late in the afternoon, they would be given a cold 24-ounce bottle of water and a bag of granola from a respected campus restaurant called The Spice Box. They were told to enjoy as much or as little of it as they wanted." Comment: No blinding or incomplete blinding of study participants and it is judged possible that awareness of the experimental condition may have influenced measurement of the outcome in this case. Very unlikely that key study personnel were blinded, but the review authors judge that the outcome is not likely to be influenced by lack of blinding of key study personnel. |
| Intention to select or purchase: Blinding of participants and personnel (performance bias) | Not measured |  |
| Intention to consume: Blinding of participants and personnel (performance bias) | Not measured |  |
| Belief associated with consumption: Blinding of participants and personnel (performance bias) | Not measured |  |
| Appeal: Blinding of participants and personnel (performance bias) | Not measured |  |
| Understanding of label: Blinding of participants and personnel (performance bias) | Unclear | Quote: "The study was conducted over ten sessions that lasted from 3:30 to 5:00 P.M. on each of ten days (Tuesdays and Thursdays for five non-consecutive weeks). On arriving at the dimly lit theater, participants were seated in every other seat and asked to watch and rate a series of made-for-television movie previews and a 60-minute pilot show called “Hazard County.” They were also told that because it was late in the afternoon, they would be given a cold 24-ounce bottle of water and a bag of granola from a respected campus restaurant called The Spice Box. They were told to enjoy as much or as little of it as they wanted." Comment: No blinding or incomplete blinding of study participants and it is judged possible that awareness of the experimental condition may have influenced measurement of the outcome in this case. Very unlikely that key study personnel were blinded, but the review authors judge that the outcome is not likely to be influenced by lack of blinding of key study personnel. |
| Selection: Blinding of outcome assessment (detection bias) | Not measured |  |
| Consumption: Blinding of outcome assessment (detection bias) | Low | Comment: No blinding of outcome assessment, but the review authors judge that the outcome measurement is not likely to be influenced by lack of blinding. |
| Intention to select or purchase: Blinding of outcome assessment (detection bias) | Not measured |  |
| Intention to consume: Blinding of outcome assessment (detection bias) | Not measured |  |
| Belief associated with consumption: Blinding of outcome assessment (detection bias) | Not measured |  |
| Appeal: Blinding of outcome assessment (detection bias) | Not measured |  |
| Understanding of label: Blinding of outcome assessment (detection bias) | Low | Comment: No blinding of outcome assessment, but the review authors judge that the outcome measurement is not likely to be influenced by lack of blinding. |
| Selection: Incomplete outcome data (attrition bias) | Not measured |  |
| Consumption: Incomplete outcome data (attrition bias) | Low | Quote: "We eliminated some participants from analysis for the following reasons: not staying until the end of the show (n = 7), refusing to eat granola because of dietary restrictions or political principles (n = 4), spilling their granola on the floor (n = 3), emptying their granola bags into their pockets (n = 3), and failing to provide height and weight information (n = 14). No participant consumed all the granola in the bag while watching the show." Comment: Reasons for excluding participants’ data from the analysis are provided in full. The review authors judge that the reason provided for these exclusions by the study authors is reasonable from the perspective of minimizing bias. |
| Intention to select or purchase: Incomplete outcome data (attrition bias) | Not measured |  |
| Intention to consume: Incomplete outcome data (attrition bias) | Not measured |  |
| Belief associated with consumption: Incomplete outcome data (attrition bias) | Not measured |  |
| Appeal: Incomplete outcome data (attrition bias) | Not measured |  |
| Understanding of label: Incomplete outcome data (attrition bias) | Low | Quote: "We eliminated some participants from analysis for the following reasons: not staying until the end of the show (n = 7), refusing to eat granola because of dietary restrictions or political principles (n = 4), spilling their granola on the floor (n = 3), emptying their granola bags into their pockets (n = 3), and failing to provide height and weight information (n = 14). No participant consumed all the granola in the bag while watching the show." Comment: Reasons for excluding participants’ data from the analysis are provided in full. The review authors judge that the reason provided for these exclusions by the study authors is reasonable from the perspective of minimizing bias. |
| Selective reporting (reporting bias) | Unclear | Comment: No evidence of selective outcome reporting based on study report. Search for record(s) containing details of study protocol conducted in ClinicalTrials.gov and the WHO International Clinical Trials Registry Platform (ICTRP) identified no record. Insufficient information to permit judgement of ‘Low risk’ or ‘High risk’. |
| Other bias | Unclear | Comment: Baseline comparability between comparison groups is not reported. Insufficient information to permit judgement of ‘Low risk’ or ‘High risk’. |
| Selection: Summary risk of bias | Not measured |  |
| Consumption: Summary risk of bias | Unclear | Comment: Unclear risk of selection bias and other bias (baseline comparability between subgroups). |
| Intention to select or purchase: Summary risk of bias | Not measured |  |
| Intention to consume: Summary risk of bias | Not measured |  |
| Belief associated with consumption: Summary risk of bias | Not measured |  |
| Appeal: Summary risk of bias | Not measured |  |
| Understanding of label: Summary risk of bias | Unclear | Comment: Unclear risk of selection bias and other bias (baseline comparability between subgroups). |

Wardle & Solomons, 1994 [*Published data only*].

| **Bias** | **Authors' judgement** | **Support for judgement** |
| --- | --- | --- |
| Random sequence generation (selection bias) | Unclear | Comment: Insufficient information about the sequence generation process to permit judgement of ‘Low risk’ or ‘High risk’. |
| Allocation concealment (selection bias) | Unclear | Comment: Insufficient information concerning method of concealment to permit judgement of ‘Low risk’ or ‘High risk’. |
| Selection: Blinding of participants and personnel (performance bias) | Not measured |  |
| Consumption: Blinding of participants and personnel (performance bias) | Low | Comment: No blinding of study participants attempted but this is not judged likely to introduce risk of performance bias in the current context. Very unlikely that key study personnel were blinded, but the review authors judge that the outcome is not likely to be influenced by lack of blinding of key study personnel. |
| Intention to select or purchase: Blinding of participants and personnel (performance bias) | Not measured |  |
| Intention to consume: Blinding of participants and personnel (performance bias) | Not measured |  |
| Belief associated with consumption: Blinding of participants and personnel (performance bias) | Not measured |  |
| Appeal: Blinding of participants and personnel (performance bias) | Low | Comment: No blinding of study participants attempted but this is not judged likely to introduce risk of performance bias in the current context. Very unlikely that key study personnel were blinded, but the review authors judge that the outcome is not likely to be influenced by lack of blinding of key study personnel. |
| Understanding of label: Blinding of participants and personnel (performance bias) | Not measured |  |
| Selection: Blinding of outcome assessment (detection bias) | Not measured |  |
| Consumption: Blinding of outcome assessment (detection bias) | Low | Comment: No blinding of outcome assessment, but the review authors judge that the outcome measurement is not likely to be influenced by lack of blinding. |
| Intention to select or purchase: Blinding of outcome assessment (detection bias) | Not measured |  |
| Intention to consume: Blinding of outcome assessment (detection bias) | Not measured |  |
| Belief associated with consumption: Blinding of outcome assessment (detection bias) | Not measured |  |
| Appeal: Blinding of outcome assessment (detection bias) | Low | Comment: No blinding of outcome assessment, but the review authors judge that the outcome measurement is not likely to be influenced by lack of blinding. |
| Understanding of label: Blinding of outcome assessment (detection bias) | Not measured |  |
| Selection: Incomplete outcome data (attrition bias) | Not measured |  |
| Consumption: Incomplete outcome data (attrition bias) | Low | Comment: No missing outcome data for consumption outcome. |
| Intention to select or purchase: Incomplete outcome data (attrition bias) | Not measured |  |
| Intention to consume: Incomplete outcome data (attrition bias) | Not measured |  |
| Belief associated with consumption: Incomplete outcome data (attrition bias) | Not measured |  |
| Appeal: Incomplete outcome data (attrition bias) | Low | Comment: No missing outcome data for product appeal outcome. |
| Understanding of label: Incomplete outcome data (attrition bias) | Not measured |  |
| Selective reporting (reporting bias) | Unclear | Comment: No evidence of selective outcome reporting based on study report. Search for record(s) containing details of study protocol conducted in ClinicalTrials.gov and the WHO International Clinical Trials Registry Platform (ICTRP) identified no record. Insufficient information to permit judgement of ‘Low risk’ or ‘High risk’. |
| Other bias | Low | Comment: Study is an individually randomised controlled trial with a crossover design. |
| Selection: Summary risk of bias | Not measured |  |
| Consumption: Summary risk of bias | Unclear | Comment: Unclear risk of selection bias. |
| Intention to select or purchase: Summary risk of bias | Not measured |  |
| Intention to consume: Summary risk of bias | Not measured |  |
| Belief associated with consumption: Summary risk of bias | Not measured |  |
| Appeal: Summary risk of bias | Unclear | Comment: Unclear risk of selection bias. |
| Understanding of label: Summary risk of bias | Not measured |  |
